# Artesunate Suppresses Choroidal Melanoma Vasculogenic Mimicry Formation and Angiogenesis *via* the Wnt/CaMKII Signaling Axis

**DOI:** 10.3389/fonc.2021.714646

**Published:** 2021-08-12

**Authors:** Bochao Geng, Yuanzhang Zhu, Yingying Yuan, Jingyi Bai, Zhizhi Dou, Aihua Sui, Wenjuan Luo

**Affiliations:** ^1^ Department of Ophthalmology, The Affiliated Hospital of Qingdao University, Qingdao, China; ^2^ Department of Physiology and Pathophysiology, School of Basic Medicine, Qingdao University, Qingdao, China; ^3^ Central Laboratory, The Affiliated Hospital of Qingdao University, Qingdao, China

**Keywords:** artesunate, choroidal melanoma, vasculogenesis mimicry, angiogenesis, VE-cadherin

## Abstract

Angiogenesis and vasculogenic mimicry (VM) are considered to be the main processes to ensure tumor blood supply during the proliferation and metastasis of choroidal melanoma (CM). The traditional antimalarial drug artesunate (ART) has some potential anti-CM effects; however, the underlying mechanisms remain unclarified. Recent studies have shown that the Wnt5a/calmodulin-dependent kinase II (CaMKII) signaling pathway has a close correlation with angiogenesis and VM formation. This study demonstrated that ART eliminated VM formation by inhibiting the aforementioned signaling pathway in CM cells. The microvessel sprouting of the mouse aortic rings and the microvessel density of chicken chorioallantoic membrane (CAM) decreased significantly after ART treatment. VM formation assay and periodic acid schiff (PAS) staining revealed that ART inhibited VM formation in CM. Moreover, ART downregulated the expression levels of the angiogenesis-related proteins vascular endothelial growth factor receptor (VEGFR) 2, platelet-derived growth factor receptor (PDGFR) and vascular endothelial growth factor (VEGF) A, and VM-related proteins ephrin type-A receptor (EphA) 2 and vascular endothelial (VE)-cadherin. The expression of hypoxia-inducible factor (HIF)-1α, Wnt5a, and phosphorylated CaMKII was also downregulated after ART treatment. In addition, we further demonstrated that ART inhibited the proliferation, migration, and invasion of OCM-1 and C918 cells. Collectively, our results suggested that ART inhibited angiogenesis and VM formation of choroidal melanoma likely by regulating the Wnt5a/CaMKII signaling pathway. These findings further supported the feasibility of ART for cancer therapy.

## Introduction

Choroidal melanoma (CM) is a common intraocular malignancy; up to 50% of patients with CM die from metastases (1). The comprehensive strategy for the clinical treatment of CM mainly involves radiotherapy and surgical resection (2). Radiotherapy is the most common globe-conserving therapy for CM, but the irradiated tumor often causes exudation and overproduction of angiogenic factors, ultimately leading to optic disk neovascularization and neovascular glaucoma (3). The main mechanism of neovascularization in CM is angiogenesis, which involves the sprouting of existing endothelial cells to form new neoplastic capillaries. Besides, some invasive tumor cells usually reside far away from the area where the primary tumor is located, and cannot be completely eliminated by surgery. These residual CM cells can induce VM formation, leading to CM recurrence. We recently reported that VM is another important neovascularization pathway in CM (4). VM is an extravascular matrix-directed circulation, which is distinct from classical angiogenesis and depends on nonendothelial cells (5). During the formation of VM, self-deformation of the highly invasive melanoma cells and extracellular matrix remodeling occur to form a vascular-like structure. Sometimes, even vessels are damaged to allow the red blood cells to enter the adjacent matrix to maintain blood supply to tumor cells (6). Patients with VM-positive tumors have poor tumor differentiation, lymph node involvement, distant metastasis, and tumor-node-metastasis (TNM) stage (7). Based on the close relationship between VM and tumor microenvironment, targeting VM inhibitors combined with antiangiogenic therapies in CM seems to be an attractive and potentially effective strategy.

The mechanisms underlying tumor angiogenesis and VM formation are extremely complex, which involve hypoxia, epithelial-mesenchymal transition (EMT), and activation of tumor-associated fibroblasts and tumor-associated macrophages. Many molecules participate in one or more of these processes that regulate tumor angiogenesis, such as VEGF, matrix metalloproteinases (MMPs), VE-cadherin, and recently emerged non-coding RNAs (8). Unlike normal tissues, most malignancies are tolerant to hypoxia. The hypoxic tumor microenvironment promotes tumor cell growth by promoting angiogenesis and immune escape. Under hypoxic conditions, HIF-1α accumulates in the nucleus, binds to the VEGF promotor, and accelerates endothelial cell growth. VEGF binds to VEGFR in epithelial cells; consequently, these cells lose their epithelial phenotype (E-cadherin and zonula occludins-1) and acquire a mesenchymal phenotype (VE-cadherin and vimentin) with MMP2 upregulation. This process is called EMT, which plays an important role in VM-forming tumor cells and promotes tumor invasion and metastasis. We reported that VEGF was also involved in VM in the development of CM (4). The Wnt signaling pathway is the downstream of VEGF. Wnt5a was overexpressed in tumor samples and associated with EMT and VM in epithelial ovarian cancer (9) and non-small-cell lung cancer (NSCLC) (10). Further, Wnt5a activated the CaMKII involved in endothelial cell biology through regulating endothelial cell [Ca^2+^] (11, 12). This has become the central interest for antiangiogenesis-based therapies. However, whether the Wnt5a/CaMKII pathway is related to angiogenesis and VM still needs to be determined.

Artemisinin is a sesquiterpene lactone extracted from *Artemisia annua* Linn. a Chinese herbal medicine used to treat malaria. ART is a water-soluble derivative of artemisinin. Recently, increasing studies have focused on the inhibitory effects of ART on angiogenesis (13). A study on chronic myeloid leukemia K562 cells showed that ART inhibited aortic sprouting and new microvessel formation in a time-dependent and dose-dependent manner, which was related to the downregulated VEGF expression and inhibited angiopoietin 1 secretion (14). However, the effect of ART on VM formation of CM and its possible molecular mechanisms are not yet known.

In this study, we confirmed that ART inhibited angiogenesis and VM formation in CM. Then, we analyzed the effects of ART on the expression of HIF-1α, VEGFR2, PDGFR, VEGFA, VE-cadherin, and EphA2 in CM cells. Finally, we analyzed whether the inhibitory effects of ART on angiogenesis and VM formation were due to its suppression of the Wnt5a/CaMKII signaling pathway. Our research revealed the potential combined blockade of angiogenesis and VM formation of ART and its possible molecular mechanisms.

## Materials and Methods

### Drug

ART powder was purchased from Sigma company (St Louis, Mo, USA), and was dissolved in dimethylsulfoxide (DMSO) at the liquor concentration of 200 x 10^3^ μM stored at -80°C. Then it was diluted with cell culture medium to the final concentration required for the experiment. The culture medium containing 0.1% DMSO was only used as a negative control.

### Cell Culture and Treatment

The human CM cell lines C918 with highly aggressive ability were obtained from Wuhan Procell Life Science&Technology Co.,Ltd. The low-aggressive human CM cell lines OCM-1 and the human retinal pigmented epithelium cell lines (ARPE-19) were purchased from Beijing BeNa Culture Collection. The ARPE-19 cell lines were incubated in DMEM medium, C918 and OCM-1 cell lines were cultured in RPMI-1640. All the culture medium was added 10% fetal bovine serum (FBS) and 1% penicillin-streptomycin. The cells cultured under normoxia were placed in the cell incubator with 5% CO_2_ and 21% O_2_; hypoxia model was constructed in a hypoxic incubator containing 94% N_2_, 5% CO_2_ and 1% O_2_. All the cell culture medium was replaced with fresh medium after 48 hours of continuous culture. When the fusion degree of cells reached approximately 80%, the cells were digested and subcultured.

### Quantitative Real Time Polymerase Chain Reaction (qPCR)

According to the manufacturer’s instruction, total mRNA in the cells was extracted using Trizol reagent. mRNA then was reverse transcribed into cDNA by the PrimeScriptTM RT Master Mix kit (Takara, Shiga, Japan) and finally used for qPCR amplification analysis with the SYBR Premix Ex Taq II (Takara, Shiga, Japan). Relative mRNA expression of VEGFA, PDGFB, CXCL1, TGFβ, SCF, IGF1 and HGF were estimated by the method of comparative amplification cycles using GAPDH as the internal reference. The 2^-∆∆Ct^ method was used for data analysis, and three independent qPCR experiments were performed. **Supplementary Table 1** shows the human-specific primer sequences used in the qPCR reaction.

### Enzyme-Linked Immunosorbent Assay (ELISA)

ELISA kit (Human VEGFA ELISA Immunoassay Kit; Mlbio, Shanghai, China) was used to analyze the concentration of VEGFA protein secreted in the tumor cell culture supernatant. And the level of VEGFA protein secretion (pg/mL) was measured using the standard curve.

### Cell Counting Kit (CCK)-8 Assay

A total number of 5x10^3^ cells in each well were seeded into 96-well plates. After the cells adhere to the wall overnight, different concentrations of ART were added to the wells. After 48 h of incubation, the original medium in the wells was replaced with 90 μL medium and 10 μL CCK-8 (Beyotime, Shanghai, China), and then the 96-well plates were returned to the incubator for further incubation for 0.5 h. Finally, a microreader was used to measure the OD value of the liquid in each well at the wavelength of 450 nm. The number of cells was directly proportional to the OD value.

### Clonogenic Assay

OCM-1 and C918 cells were pretreated with different concentrations of ART. Then a density of 200 cells/well were plated into six-well plates and cultured for 10-14 days. 4% paraformaldehyde and crystal violet (0.01% w/v) were used to fix and stain the colonies. The colonies that reached the number of more than 50 cells were counted.

### Wound-Healing Assay

The cells suspension with a density of approximately 3x10^5^ cells/mL was uniformly plated into six-well plates. After the cells confluence rate reached 90%, a straight-line scratch with the same width was made by a 10μL pipette tip on the bottom of the culture plates. The shed cells were washed away with PBS, and then the scratched cells were cultured in RPMI 1640 medium (without FBS) for 24 h, and different concentrations of ART were added. To more intuitively judge the migration distance of cells and evaluate the cells migration ability, 4% formaldehyde and 0.1% crystal violet were used to fix and stain cells after 24 h of incubation. The scratch area at 0, 12, and 24h was measured with Image J respectively to calculate the cell migration ability.

### Cell Migration and Invasion Assays

OCM-1 and C918 cells treated with different concentrations of ART for 24 h, and then FBS-free medium was used for the preparation of cell suspension. 1x10^5^ cells were added to the upper chamber for migration assays (24-well insert; pore size 8μM; Corning, New York, USA). The cell invasion assays required to have the pre-coating membrane with Matrigel, 2x10^5^ cells were added in the upper chamber after the gel had been polymerized. Medium containing 10% FBS was added to the lower chamber as a chemo-attractant. After 24 h of incubation, 4% formaldehyde and 0.1% crystal violet were used to fix and stain cells that invaded or migrated to the lower surface of the membrane. Finally, 8 fields of view were randomly selected under an inverted microscope to count the migrated and invasive cells and calculated the average number of cells in each field. All experiments were repeated three times.

### VM Formation Assay and PAS-Staining

50μL/well Matrigel was added to 96-well plates, incubated for 30 min to allow the gel to completely been polymerized; then 2×10^4^cells/well were resuspended in a medium containing different concentrations of ART and added to the surface of the Matrigel gel. After 24 h of incubation, three fields of view were randomly selected to take pictures under the inverted microscope and the tube formation rate was calculated. To identify whether the three-dimensional vascular networks were surrounded by tumor cells, the three-dimensional vascular loops were stained with PAS and photographed with the inverted microscope.

### Mouse Aortic Ring Assay

To study the effect of ART on angiogenesis *ex vivo*, the previous protocol of the mouse aortic ring assay was used for reference and slightly modified (15). C57BL/6 mice aged 8-12 weeks were selected for dissection of the thoracic aorta, and the lumen was washed with Opti-MEM medium and cut into rings with the thickness of 1mm. These rings were then placed in 96-well plates, embedded in 50 μL Matrigel (Corning Corp., Bedford, MA, USA), and incubated at 37°C for 1 h to fully polymerize the gel. Different concentrations of ART were added to the rings, after 8 days of incubation, the 96-well plates were taken out to observe the germination of microvessels under the microscope and capture photographs.

### Chicken Chorioallantoic Membrane (CAM) Assay

The previous CAM-assay protocol was slightly modified to evaluate the effect of ART on angiogenesis *in vivo* (16). In brief, fertilized eggs were incubated at 37°C (humidity 65-70%) for 3 days, the eggshell was removed to make a window of 1 cm^2^, and then the window was sealed with adhesive tape. The eggs were returned to the incubator and stabilized for another 3 days. On day 7, 100µL ART (15nmol) or 100µL saline (negative control) was added to the CAM and incubated for 24 h, Finally, the window was enlarged and photographs were taken.

### Wnt5a siRNA Transfection

To further assess the role of Wnt5a in CM cells, we used an siRNA-based technique to specifically silence Wnt5a expression in OCM-1 and C918 cells. Wnt5a small interfering RNA (siRNA-Wnt5a) and negative control small interfering RNA (siRNA-NC) were purchased from GenePharma Company (Shanghai, China). Transfection was performed using Lipofectamine 3000 transfection reagent (Invitrogen, Carlsbad, CA, USA) in Opti-MEM medium (Gibco, Rockville, MD, USA) following the manufacturer’s instructions. Cells were exposed to ART 24 h after transfection. Subsequently, we harvested the transfected cells for further experiments. The siRNAs target sequences were as follows: si-Wnt5a-#1, sense, 5’-GCUACGUCAAGUGCAAGAATT-3’, antisense, 5’-UUCUUGCACUUGACGUAGCTT-3’; si-Wnt5a-#2, sense, 5’-GAAGUCCAUUGGAAUAUUATT-3’, antisense, 5’-UAAUAUUCCAAUGGACUUCTT-3’.

### Western Blotting

After 24 h of treatment with different concentrations of ART, the cells were rinsed with PBS three times and then lysed with RIPA (Solarbio, Beijing, China) for 30 minutes. The cell lysates were collected and centrifuged at 4°C for 15min at 12000g. The supernatants after centrifugation were standardized with BCA protein analysis kit for the protein concentrations. SDS-PAGE separated the protein samples and transferred them to PVDF membranes (Merck Millipore, Billerica, MA, USA). The membrane was blocked with 5% nonfat milk at room temperature for 2h, and then incubated overnight at 4°C with primary antibodies: Wnt5a (Cell Signaling Technology, Danvers, MA, USA), Phospho-CaMKII (p-Thr286, Cell Signaling Technology), CaMKII (Hangzhou HuaAn Biotechnology, Hangzhou, China), HIF-1α (Proteintech, Rosemont, IL, USA), VE-cadherin (Affinity Biosciences, OH, USA), EphA2 (Hangzhou HuaAn Biotechnology), VEGFA (Affinity Biosciences), VEGFR2 (Proteintech), and PDGFR (Proteintech). The membranes were rinsed three times with PBST and incubated with goat anti-rabbit secondary antibody (Cell Signaling Technology) for 1 hour at room temperature. Finally, the membranes were washed three times with PBST and used for luminescence imaging with the chemiluminescence detection device (Merck Millipore Corporation, Darmstadt, Germany). ImageJ software (Bethesda, MD, USA) was used to analyze the gray value of the target protein bands, and the corresponding GAPDH gray value was used as an internal reference.

### Statistical Analysis

All experiments were repeated at least three times unless specified. The unpaired *t* test or one-way analysis of variance (ANOVA) in Prism 7.0 (GraphPad, USA) were used for statistical analysis of the experimental data, and the data are provided as the mean ± SEM (standard error of the mean). P-values less than 0.05 were considered to indicate statistical significance (*P<0.05, **P<0.01, ***P<0.001).

## Results

### Expression Profile of Pro-Angiogenic Factors in OCM-1 Cell Lines Under Hypoxic and Normoxic Conditions

**Figure 1:**
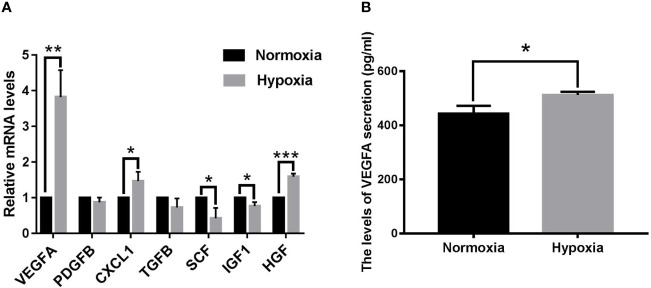
Seven classical pro-angiogenic factors mRNA levels and VEGFA protein secretion in OCM-1 cell lines under hypoxic and normoxic conditions. **(A)** mRNA expression levels of seven classical pro-angiogenic factors in OCM-1 cells were detected by qPCR after 6 h of treatment under normoxic (black) and hypoxic (grey) conditions. GAPDH was used as an internal control. Data from at least three independent samples were represented as mean ± SEM; *P < 0.05, **P < 0.01, ***P < 0.001. **(B)** Secretion level of VEGFA protein in the culture supernatants of OCM-1 cells under normoxic (black) and hypoxic (gray) conditions for 6h was measured with ELISA. Similar data were detected in three independent samples (mean ± SEM); *P < 0.05.

The hypoxia microcirculation of malignant tumors is the rate-increasing step of tumor growth and hematological dissemination. Hence, we used quantitative real time polymerase chain reaction (qPCR) to analyze the mRNA expression levels of seven pro-angiogenic factors in human CM cell lines OCM-1 under hypoxic and normoxic conditions. As shown in **Figure 1A**, the VEGFA mRNA expression level of OCM-1 cell lines under hypoxic conditions increased nearly four-fold compared with that under normoxic conditions. To evaluate whether the differential expression of VEGFA mRNA in OCM-1 cells affected its protein production, we used ELISA to analyze the protein expression of VEGFA in the culture supernatant of OCM-1 cell lines under hypoxic and normoxic conditions. The ELISA results were consistent with the qPCR data (**Figure 1B**). Taken together, these results suggested a crucial role for VEGFA in promoting the growth and development of CM.

### ART Suppressed the Viability and Reduced the Migration and Invasion of OCM-1 and C918 Cell Lines

**Figure 2:**
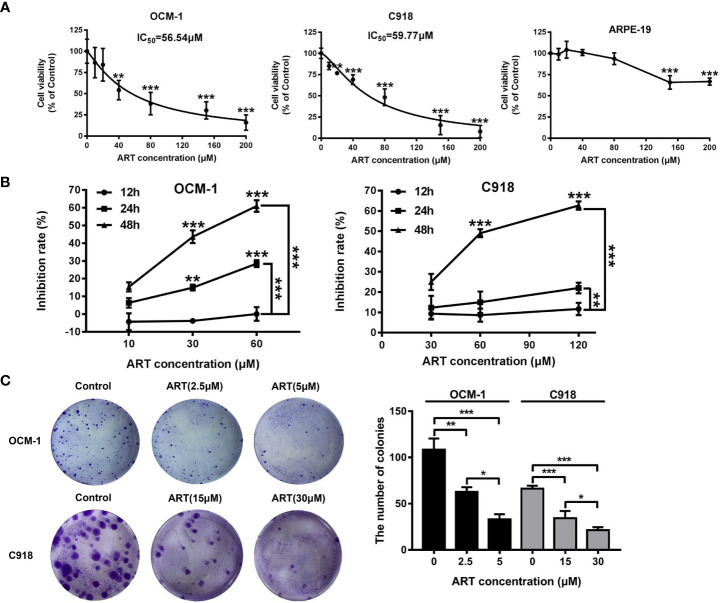
Proliferation inhibition by ART in CM cells. ART selectively killed CM cells but not human retinal pigment epithelial cells. **(A)** OCM-1 and C918 cells were treated with different concentrations of ART for 48 h, and the cell viability was measured by the CCK-8 assay. The human ARPE-19 cells were set as a negative control. The data are from three independent experiments (mean ± SEM); *P < 0.05, **P < 0.01, ***P < 0.001. **(B)** ART specifically inhibited the growth of CM cell lines in a dose-dependent and time-dependent manner. **(C)** OCM-1 and C918 cell lines were pretreated with ART and plated in six-well plates for 10-14 days. Further, 4% paraformaldehyde and 0.2% crystal violet were used for fixation and staining. The statistical data from three independent colony formation assay are represented as mean ± SEM. *P < 0.05, **P < 0.01, ***P < 0.001.

**Figure 3:**
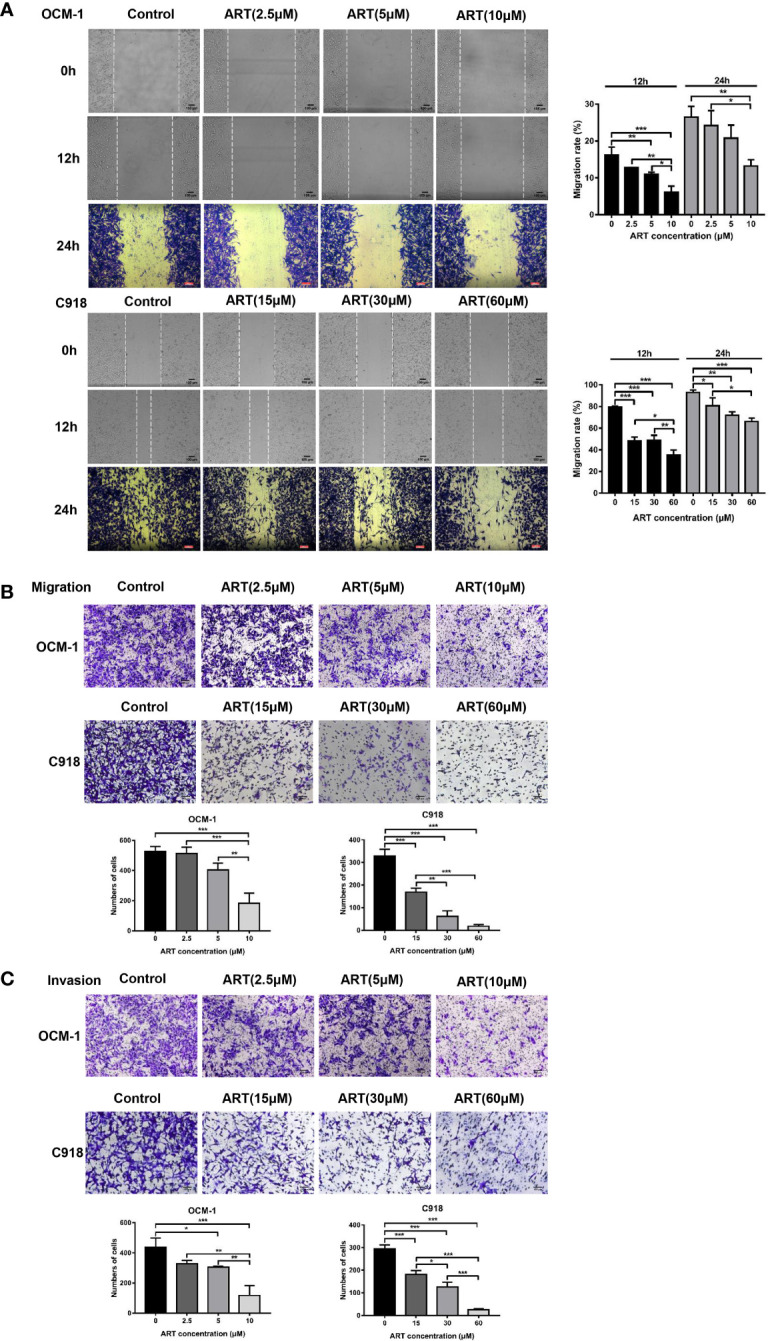
ART reduced the migration and invasion of CM cells. **(A)** ART significantly inhibited the wound-healing ability in CM cells. OCM-1 and C918 cells treated with different concentrations of ART were subjected to the wound-healing assay. Photos were taken in the same field after 0, 12, and 24 h. Further, 4% formaldehyde and 0.1% crystal violet were used to fix and stain cells after 24 h of incubation. **(B)** OCM-1 and C918 cells were pretreated with indicated concentrations of ART for 24 h; they were seeded into the upper chamber of Transwell and incubated for 24 h. After fixing with 4% formaldehyde and staining with 0.1% crystal violet, the cells migrating to the lower chamber were counted. **(C)** ART inhibited OCM-1 and C918 cell invasion according to the Transwell invasion assay. The statistic results are represented as mean ± SEM from three independent samples. *P < 0.05, **P < 0.01, ***P < 0.001.

To investigate the effect of ART on the cell viability of OCM-1 and C918 cells, the cells were treated with different concentrations of ART (0, 10, 20, 40, 80, 150, and 200μM) for 48 h, and then the CCK-8 assay was used to measure cell viability. The IC_50_ values of ART in OCM-1 and C918 cells was 56.54 and 59.77μM for 48h of exposure, respectively (**Figure 2A**). In addition, we also examined the effects of ART on the cell viability of ARPE-19 to distinguish the inhibitory effect and toxicity of ART. Our results showed that the effect of ART on ARPE-19 cell viability was relatively weak (**Figure 2A**). In detail, the inhibitory effect of ART on OCM-1 and C918 cell viability was both concentration-dependent and time-dependent (**Figure 2B**). To avoid cytotoxicity of ART for further study, we chose 10 and 60µM ART as the maximum concentrations of OCM-1 and C918 cells for subsequent experiments *in vitro*, which was based on an inhibition rate of 10% for 24 h in OCM-1 and C918 cells. Three ART concentration gradients were set for each cell lines. Since the colony formation assay can better show the malignant characteristics of tumor cells, we analyzed the effect of ART on clonogenicity in OCM-1 and C918 cells. Our data indicated that ART inhibited the clonogenicity of CM cells in a concentration-dependent manner (**Figure 2C**). These results were in line with the findings of the CCK-8 assay. The migration and invasion of tumor cells are crucial for the formation of VM by CM cells. Scratch wound-healing, cell migration, and invasion assays showed that ART had a significant inhibitory effect on the migration and invasion ability of OCM-1 and C918 cell lines (**Figure 3**). The cell migration rates in the scratch wound-healing assays and the data of Transwell invading and migrating cells were presented using bar graphs. Our results showed that ART prevented the initial stage of VM formation by inhibiting the proliferation, invasion, and migration of CM cells.

### ART Inhibited VM Formation in OCM-1 and C918 Cells and Reduced Sprouting of the Mouse Aortic Ring and CAM Neovascularization

**Figure 4:**
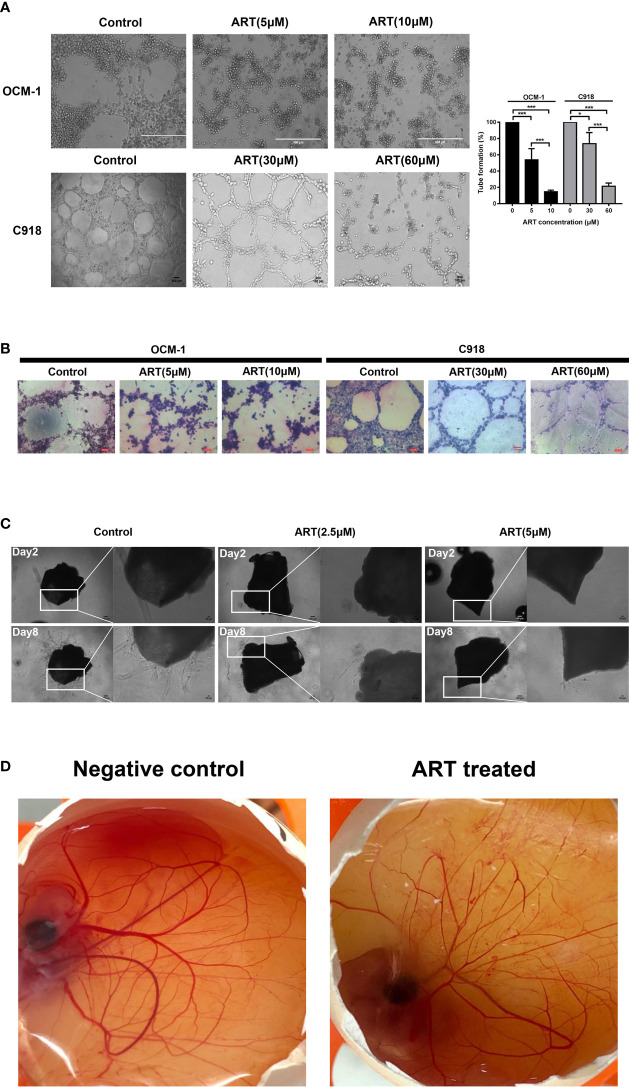
ART inhibited VM formation and angiogenesis. **(A)** OCM-1 and C918 cells treated with indicated concentrations of ART were subjected into three-dimensional Matrigel culture for 24 h. Three fields of view were randomly selected to take pictures under the inverted microscope, and the tube formation rate was calculated (n = 3). *P < 0.05, ***P < 0.001. **(B)** PAS stained the tubules surrounded by C918 and OCM-1 cells on Matrigel (n=3). **(C)** Images of aortic rings embedded in Matrigel. Time course of microvessel sprouting from aortic rings embedded in Matrigel and treated with indicated concentrations of ART. Images were obtained using a microscope from days 2 to 8 after embedding (n = 3). **(D)** Central CAM section images of the negative control (normal saline) group and ART treatment (15nmol/egg) group on day 8 (n=20).

OCM-1 and C918 cells can be efficiently lined into tubular structures on Matrigel, our results showed that these vasculogenic networks in CM cells can be suppressed by ART in a concentration-dependent manner (**Figure 4A**). PAS-positive staining can be detected along with the tubular structures, indicating that the degradation of extracellular matrix was involved in the regulation of VM formation (**Figure 4B**). To further explore the inhibitory effect of ART on the viability of vascular endothelial cells, then we constructed both mouse aortic ring sprouting and CAM neovascularization models. The results showed that ART significantly suppressed microvessel sprouting of the mouse aortic rings in a dose-dependent manner *ex vivo* (**Figure 4C**). Similarly, ART also showed excellent angiogenesis inhibition in the CAM model. After the CAMs were treated with 15nmol/egg ART for 24h, the microvessel density and the number of vascular branches decreased significantly (**Figure 4D**). These results validate that ART can not only inhibit the VM formation of tumor cells, but also has a strong inhibitory effect on angiogenesis both *ex vivo* and *in vivo*.

### ART Blocked the Wnt5a/CaMKII Signaling Pathway in OCM-1 and C918 Cell Lines

**Figure 5:**
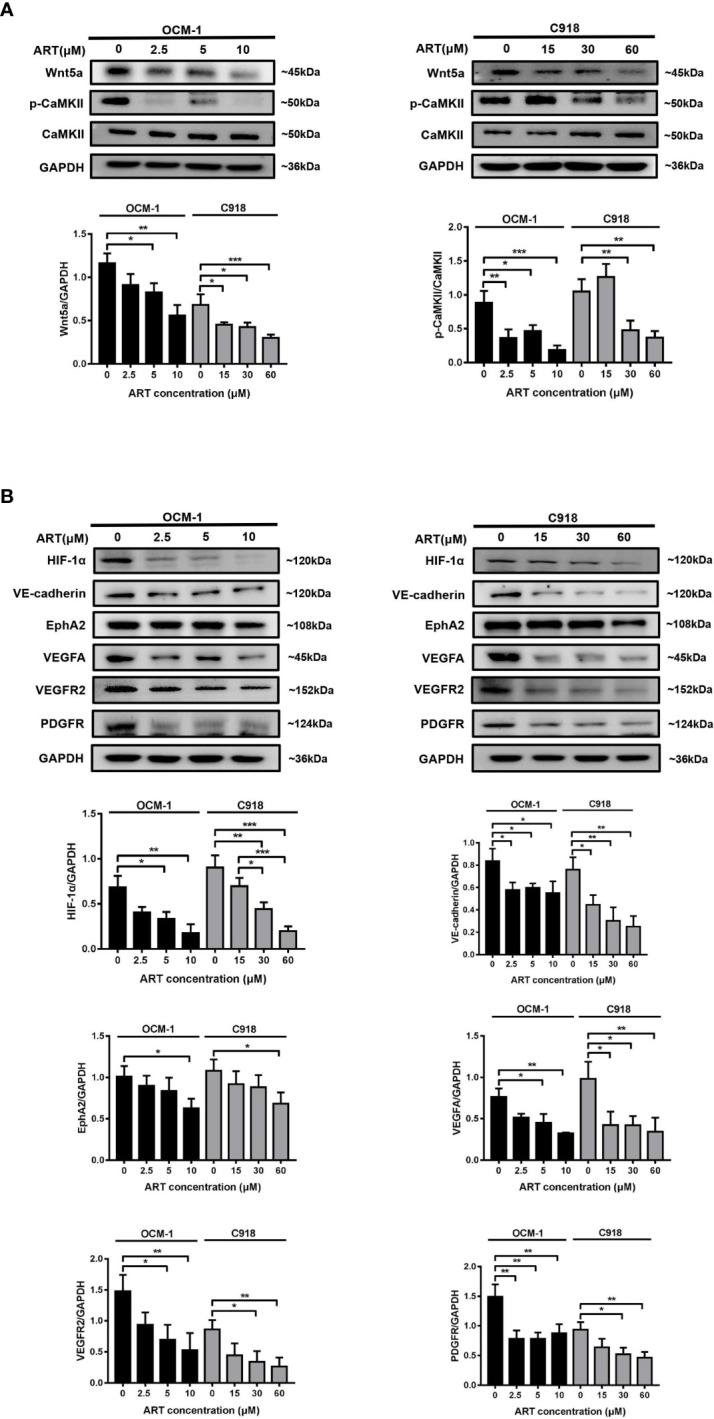
ART inhibits the Wnt5a/CaMKII signaling pathway in CM cells. **(A, B)** OCM-1 and C918 cells were treated for 24 h with ART. Western blotting showed that ART suppressed the protein expression levels of Wnt5a, p-CaMKII, HIF-1α, VE-cadherin, EphA2, VEGFA, VEGFR2, and PDGFR in OCM-1 and C918 cells. GAPDH was used as an internal control. The results are represented as the mean ± SEM from three independent samples. *P < 0.05, **P < 0.01, ***P < 0.001.

HIF-1α has a direct regulatory effect on VM formation and can modulate the expression levels of VE-cadherin, EphA2, VEGFR2, and VEGFA in tumors (17). Wnt5a/CaMKII signaling pathway has been regarded as a key regulator of retinal vascularization induced by growth factors (18). In the present study, we detected the effect of the Wnt5a/CaMKII signaling pathway after the ART treatment of OCM-1 and C918 cell lines. The data revealed that the expression levels of Wnt5a and phospho-CaMKII protein markedly reduced (**Figure 5A**). In addition, treatment with ART significantly suppressed HIF-1α, VE-cadherin, EphA2, VEGFA, VEGFR2, and PDGFR levels in OCM-1 and C918 cells (**Figure 5B**).

### ART Interrupted VM Formation in CM Cells *via* the Wnt5a/CaMKII Signaling Pathway

**Figure 6:**
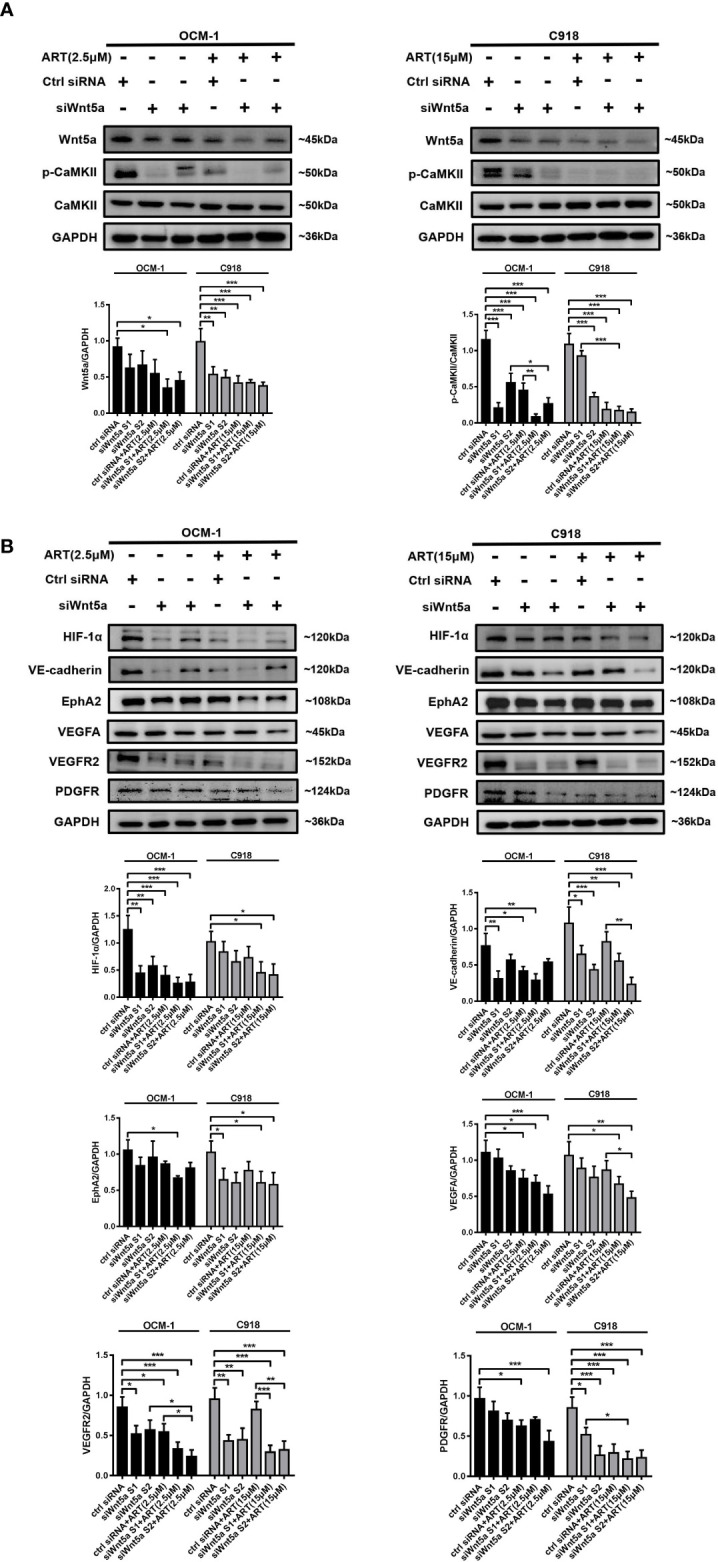
The regulation of Wnt5a/CaMKII signaling pathway in CM cells by ART. **(A, B)** Western blotting was performed after OCM-1 and C918 cells were transfected with Wnt5a siRNA or scrambled siRNA for 48 h. The results of Western blotting showed that transfected with Wnt5a siRNA effectively reduced Wnt5a protein levels and the expression levels of phosphorylated CaMKII, HIF-1α, VE-cadherin, EphA2, VEGFA, VEGFR2, and PDGFR in OCM-1 and C918 cells compared with the cells transfected with scrambled siRNA. Treatment with ART markedly enhanced the inhibitory effect of Wnt5a/CaMKII signaling pathway related protein levels. The data are expressed as the mean ± SEM from three independent samples. *P < 0.05, **P < 0.01, ***P < 0.001.

**Figure 7:**
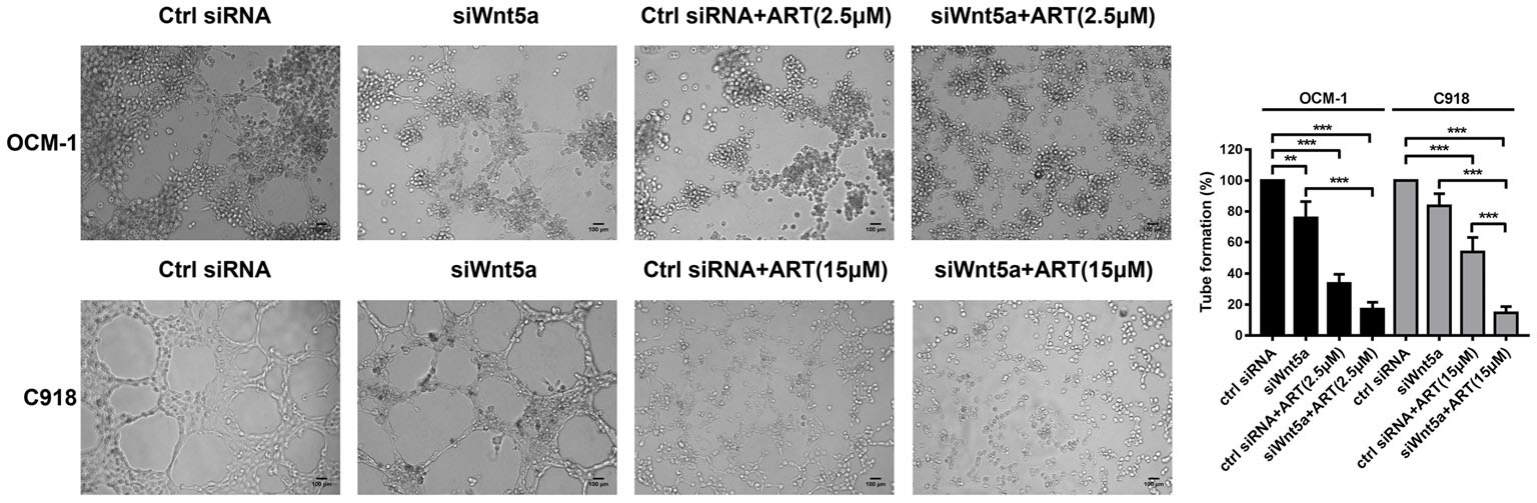
ART interrupted VM formation in CM cells *via* Wnt5a/CaMKII pathway. OCM-1 and C918 cells transfected with Wnt5a siRNA or scrambled siRNA subsequently seeded on three-dimensional Matrigel layer culture for 24 h. OCM-1 and C918 cells transfected with Wnt5a siRNA showed a decrease in the number of tubes formed compared with cells transfected with scramble siRNA. Compared with the cells transfected only with small interfering RNA, transfected cells treated with ART have an enhanced inhibitory effect on tube formation. The results are represented as the mean ± SEM of three independent samples. **P < 0.01, ***P < 0.001.

To analyze whether ART inhibits VM formation of CM depending on the Wnt5a/CaMKII signaling pathway. We used an siRNA-based technique to specifically silence Wnt5a expression in CM cells. The result of Western blotting showed that transfected with Wnt5a siRNA effectively reduced the protein levels of Wnt5a (**Figure 6A**). Moreover, the expression levels of phosphorylated CaMKII, HIF-1α, VE-cadherin, EphA2, VEGFA, VEGFR2 and PDGFR in OCM-1 and C918 cells transfected with Wnt5a siRNA were lower than the levels in cells transfected with scrambled siRNA (**Figures 6A, B**). The results showed a decrease in the number of tubes formed in CM cells transfected with Wnt5a siRNA compared with cells transfected with Scramble siRNA (**Figure 7**). In addition, we also treated the transfected cells with ART subsequently seeded on Matrigel layer. The number of tubes formed decreased after ART treatment compared with that in the cells transfected only with small interfering RNAs (**Figure 7**).

Treatment with ART markedly enhanced the inhibitory effect of angiogenesis and VM marker protein levels (**Figures 6A, B**). Taken together, our findings indicated that the inhibition of VM formation by ART was partly due to the suppression of Wnt5a/CaMKII signaling in CM cells.

## Discussion

CM develops in the capillary-rich tissues of the body lacking lymphatic vessels; it has been serious concern as purely hematogenous dissemination, but not lyphangiogenesis (19). Thus, the adequate blood supply is of significance for the growth and metastasis of CM. VM is a new tumor microcirculation mode formed by highly aggressive tumor cells, which differs from the traditional endothelium-dependent angiogenesis; it is closely related to tumor cell invasion, migration, and poor clinical prognosis (20, 21). The current anti-VEGF therapy that targets abnormal angiogenesis seems to be able to alleviate some cases of endothelium-induced angiogenesis. However, the conventional blockade of VEGF therapy did not inhibit VM in CM. Therefore, a combined therapy targeting VM formation and angiogenesis based on its incentives to prevent tumor blood supply urgently needs to be developed. ART has been reported to inhibit iris and retinal neovascularization of rabbits by blocking the expression of VEGFR2, PDGFR and protein kinase Cα (PKCα) (22). Several researchers have reported the effect of ART on angiogenesis in both preclinical *in vitro* and *in vivo* models (23–25). ART significantly reduced the expression of VEGF in tumor cells, and the expression of VEGFR2 in endothelial cells, and reduced microvessel density in xenograft tumors (26, 27). Anfosso performed a hierarchical cluster analysis of genes related to ART and artemisinin sensitivity and found that the expression of angiogenesis-related genes highly correlated with drug response (28). Our study evaluated the anti-angiogenic effect of ART in vascular endothelial cells. The microvessel sprouting of mouse aortic rings *ex vivo* and the CAM neovascularization *in vivo* were used for verification. All these results indicated that ART might inhibit tumor growth by suppressing angiogenesis and VM formation in CM. Most importantly, it had a favorable safety profile in human retinal pigment epithelial (ARPE-19) cells.

As reported for CM, hypoxia regulates the main signaling pathways involved in tumor progression and resistance to therapies. Many studies reported the essential role of hypoxia in regulating angiogenesis, VM, and response to therapy in melanoma (29). Hypoxia-inducible factors (HIFs), as a group of transcriptional activators, have emerged as the main regulators of these hypoxia-regulated angiogenic stimulators (30). However, HIF-1α was not restricted to hypoxic regions. As previously reported, the expression of HIF-1α in cells grown under normoxic conditions and in well-vascularized regions demonstrated the correlation of this protein, and more generally of its target genes, at the beginning of CM growth; when hypoxic areas are not yet obvious (29). After uveal melanoma cells were exposed to hypoxia, the expression and nuclear localization of HIF-1α increased, targeting VEGF genes, including VEGF-A, for its transcription (31, 32). VEGFA has been demonstrated to regulate the proliferation, migration, and survival of vascular endothelial cells by activating its receptor VEGFR2 on endothelial cells (33, 34). Previous studies have shown that VEGFR2 plays a key role in maintaining the “stemness” of glioma stem cell-like cells during VM formation and tumorigenesis (35, 36). VEGFR2 was also demonstrated to play the most important role in promoting endothelial cell mitogenesis and retinal vessels permeability (37). We cultured OCM-1 cells under hypoxia conditions to demonstrate the effect of hypoxia on the expression of pro-angiogenic factors in CM cells, and confirmed that VEGFA was one of the most significant genes in CM.

Evidence that shows new molecular targets, such as platelet-derived growth factor (PDGF), VE-cadherin and EphA2, as well as other hypoxia-regulated gene products, also provide avenues for improving the therapeutic effect of anti-VM strategies (38). PDGF has been demonstrated to be an important therapeutic target for treating ocular neovascularization (39, 40). Blocking the PDGF pathway led to the inhibition of corneal neovascularization (41), and increased the effect of the VEGF pathway blockade by bevacizumab (42). In addition, VE-cadherin and EphA2 were the first two proteins identified as having a role in mediating melanoma VM (43, 44). Studies aimed at testing the role of these proteins in promoting VM in melanoma have shown that the down-regulation of VE-cadherin or EphA2 inhibits VM. Furthermore, it has been shown that VE-cadherin can regulate the position and level of EphA2 phosphorylation, providing the first evidence that signal transduction from the plasma membrane is necessary for melanoma VM (45). Moreover, EphA2 has also been shown to mediate VEGF expression and VEGF-induced angiogenesis in breast cancer and pancreatic islet cancer cells, which suggests that EphA2 may promote the plasticity of tumor cells in some cases (46). Considering the diversity of tumor vascular perfusion pathways, drugs targeting HIF-1α, angiogenesis, or VM represent an attractive therapeutic target in CM. In this study, we confirmed that ART significantly inhibited CM cell proliferation and tube formation in a dose-dependent manner. In addition, ART obviously reduced the expression of HIF-1α, VEGFR2, PDGFR, VEGFA, VE-cadherin, and EphA2 in CM cells.

Our further mechanistic study showed that the ART-induced inhibition of VM formation and angiogenesis might be due to the suppression of the Wnt5a/CaMKII pathway. This was because blocking the Wnt5a/CaMKII pathway using Wnt5a siRNA on CM cells led to the suppression of VM formation and the expression of HIF-1α, VEGFR2, PDGFR, VEGFA, VE-cadherin, and EphA2, indicating the potential relationship between Wnt5a/CaMKII pathway and pathological angiogenesis (47). Recent studies have shown that Wnt family members play a key role in vascular endothelial cell differentiation, angiogenesis, and vascular development, especially the non-canonical Wnt5a/CaMKII signaling pathway (48–50). Wnt5a, as a non-canonical Wnt ligand, also regulates the signaling of VEGF-A, a hypoxia-inducible cytokine involved in retinal vascularization and development of retinal blood vessels (51). CaMKII is recognized as a key regulator of retinal angiogenesis induced by growth factors *in vivo* and *in vitro*. In addition, VEGF-induced tube formation can be inhibited by the knockdown of CaMKII expression in adult retinal microvascular endothelial cells (52). In melanoma cells, Wnt5a signaling enhances tumor angiogenesis, which involves a Ca^2+^-dependent release of exosomes containing pro-angiogenic proteins, VEGF and MMP2, whereas the promotion of exosomal secretion by Wnt5a can be inhibited by the calcium chelator Bapta (53). Wnt5a is also involved in regulating angiogenesis through the Wnt5a/β-catenin signaling pathway. The overexpression of Wnt5a activates the canonical Wnt signaling pathway by inducing the nuclear accumulation of β-catenin, increases the expression of the target genes, such as VE-cadherin, MMP2, and MMP9, and accelerates angiogenesis, ultimately leading to the growth and metastasis of NSCLC (10). Joo-Hyun et al. found that Wnt5a antagonized the Wnt/β-catenin pathway by inducing β-catenin phosphorylation/degradation in retinal pigment epithelium cells (18). Moreover, retinal myeloid cells were shown to suppress retinal angiogenesis by inhibiting the Wnt5a/VEGFR2 pathway (54). These results revealed that HIF-1α, VEGFR2, PDGFR, VEGFA, VE-cadherin, and EphA2 promoted VM formation in CM through CaMKII phosphorylation and Wnt5a activation. ART likely interrupted VM formation in OCM-1 and C918 cells partly by blocking the Wnt5a/CaMKII pathway.

**Figure 8:**
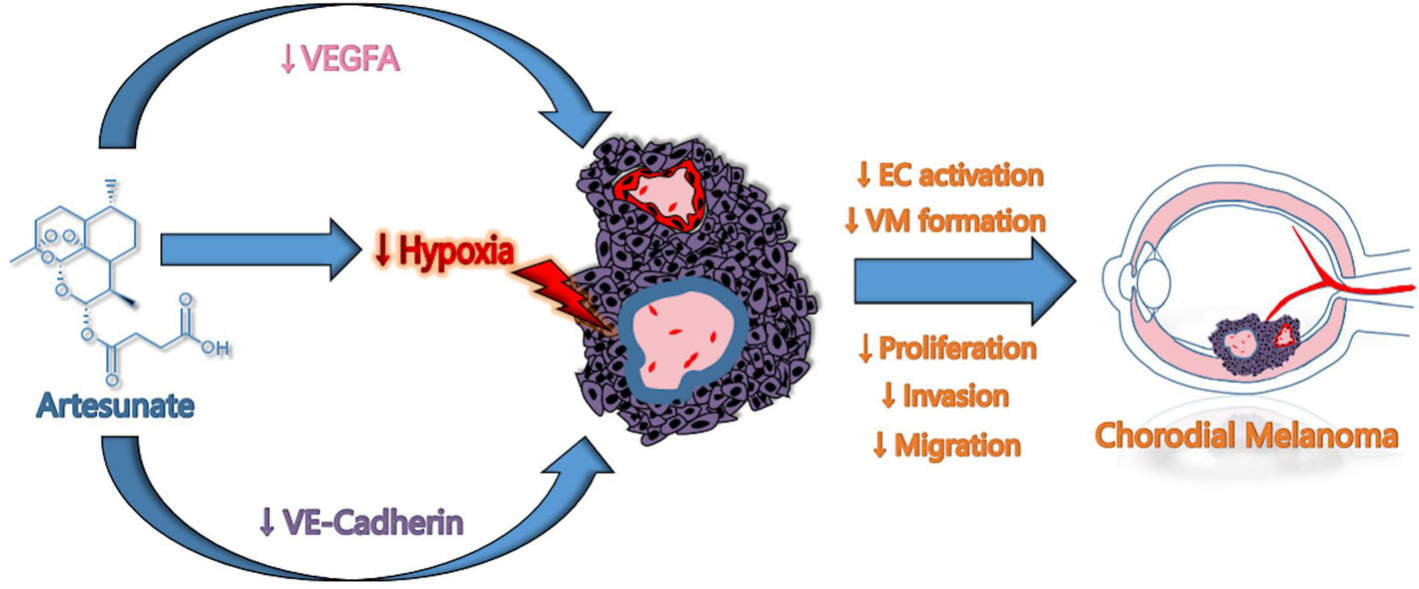
Schematic model of proposed mechanisms by which ART suppressed VM and angiogenesis in CM.

In summary, this study elucidated a new underlying mechanism of ART anti-CM: ART suppressed angiogenesis and VM formation of CM through a mechanism probable related to the inhibition of the Wnt/CaMKII signaling pathway, subsequently inducing the degradation of HIF-1α resulting in the reduction of VEGFR2, PDGFR, VEGFA, VE-cadherin and EphA2 expression. Eventually, the critical steps of CM cells in angiogenesis and VM formation were inhibited, including tumor cell proliferation, invasion, and migration (**Figure 8**). This study indicated that ART could potentially improve the therapeutic effect of anti-VEGF alone in the current CM treatment; and provided a more reasonable treatment strategy for patients who were resistant to these drugs. Nevertheless, the exact anti-CM mechanisms of ART remain to be further explored.

## Data Availability Statement

The original contributions presented in the study are included in the article/**Supplementary Material**. Further inquiries can be directed to the corresponding author.

## Ethics Statement

The animal study was reviewed and approved by the Ethics Committee of the Affiliated Hospital of Qingdao University (permit number: QYFYWZLL26381).

## Author Contributions

BG and WL: responsible for generation of hypothesis and experimental design of all experiments; they conducted most of the experiments and wrote and revised the manuscript. YZ, YY, JB, ZD, and AS: contributed to the experimental design and data analysis and contributed to manuscript preparation. All authors contributed to the article and approved the submitted version.

## Funding

The research was funded by the National Natural Science Foundation of China (Grant No. 81873345), the National Natural Science Foundation of China (Grant No. 81772633) and the Taishan Scholars Program of Shandong Province (Grant No. ts20190987).

## Conflict of Interest

The authors declare that the research was conducted in the absence of any commercial or financial relationships that could be construed as a potential conflict of interest.

## Publisher’s Note

All claims expressed in this article are solely those of the authors and do not necessarily represent those of their affiliated organizations, or those of the publisher, the editors and the reviewers. Any product that may be evaluated in this article, or claim that may be made by its manufacturer, is not guaranteed or endorsed by the publisher.
