# Corrigendum: Artesunate Suppresses Choroidal Melanoma Vasculogenic Mimicry Formation and Angiogenesis *via* the Wnt/CaMKII Signaling Axis

**DOI:** 10.3389/fonc.2022.870805

**Published:** 2022-03-01

**Authors:** Bochao Geng, Yuanzhang Zhu, Yingying Yuan, Jingyi Bai, Zhizhi Dou, Aihua Sui, Wenjuan Luo

**Affiliations:** ^1^Department of Ophthalmology, The Affiliated Hospital of Qingdao University, Qingdao, China; ^2^Department of Physiology and Pathophysiology, School of Basic Medicine, Qingdao University, Qingdao, China; ^3^Central Laboratory, The Affiliated Hospital of Qingdao University, Qingdao, China

**Keywords:** artesunate, choroidal melanoma, vasculogenesis mimicry, angiogenesis, VE-cadherin

In the original article, there was a mistake in **Figure 7** as published. We recognized by ourselves that the image of the Ctrl siRNA group in OCM-1 cells was misused. The corrected **Figure 7** appears below.

**Figure 7:**
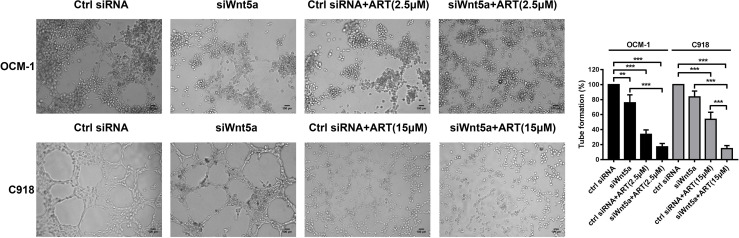
ART interrupted VM formation in CM cells *via* Wnt5a/CaMKII pathway. OCM-1 and C918 cells transfected with Wnt5a siRNA or scrambled siRNA subsequently seeded on three-dimensional Matrigel layer culture for 24 h. OCM-1 and C918 cells transfected with Wnt5a siRNA showed a decrease in the number of tubes formed compared with cells transfected with scramble siRNA. Compared with the cells transfected only with small interfering RNA, transfected cells treated with ART have an enhanced inhibitory effect on tube formation. The results are represented as the mean ± SEM of three independent samples. ^**^P < 0.01, ^***^P < 0.001.

The authors apologize for this error and state that this does not change the scientific conclusions of the article in any way. The original article has been updated.

## Publisher’s Note

All claims expressed in this article are solely those of the authors and do not necessarily represent those of their affiliated organizations, or those of the publisher, the editors and the reviewers. Any product that may be evaluated in this article, or claim that may be made by its manufacturer, is not guaranteed or endorsed by the publisher.

