# Supplementary material for: Artesunate Suppresses Choroidal Melanoma Vasculogenic Mimicry Formation and Angiogenesis via the Wnt/CaMKII Signaling Axis
Source: Front Oncol. 2021 Aug 12;11:714646. doi: 10.3389/fonc.2021.714646 (PMC8406848; doi:10.3389/fonc.2021.714646)
Supplement: Supplementary file 1 [file DataSheet_1.zip › Supplementary materials/raw data.pptx]

## Slide 1
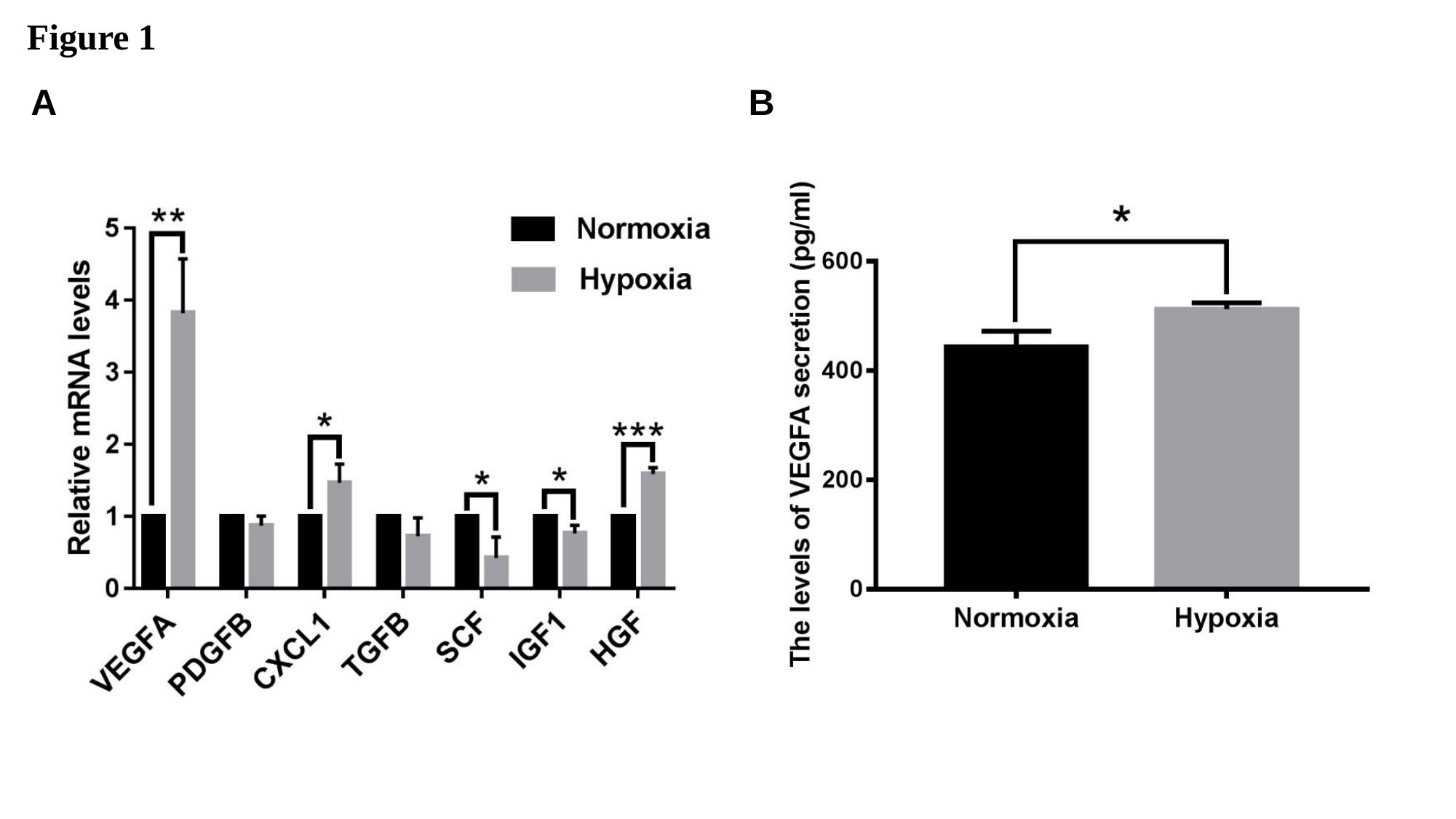

# Figure 1
A
B

## Slide 2
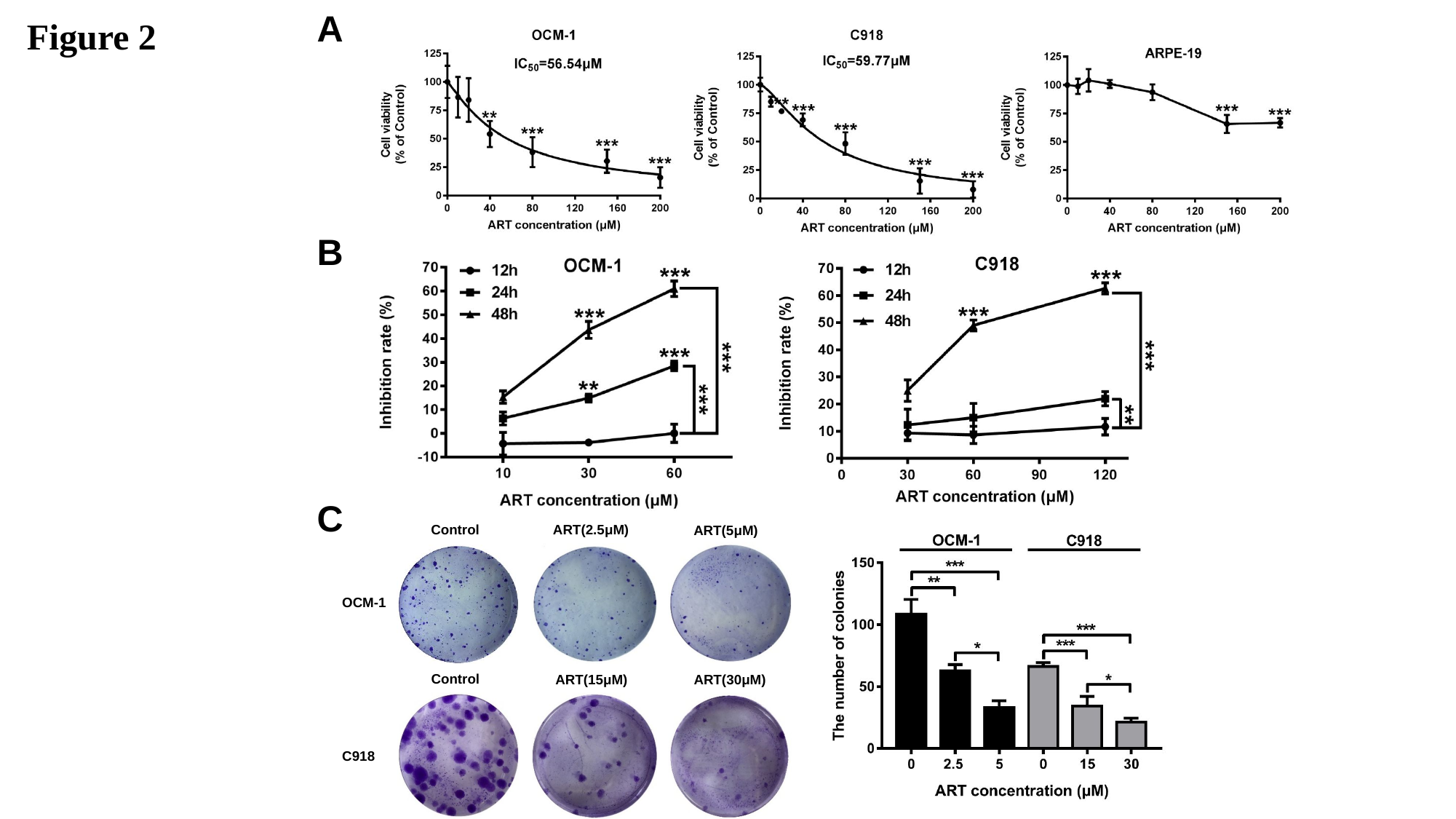

A
# Figure 2
B
C
Control
ART(2.5μM)
ART(5μM)
OCM-1
Control
ART(15μM)
ART(30μM)
C918

## Slide 3
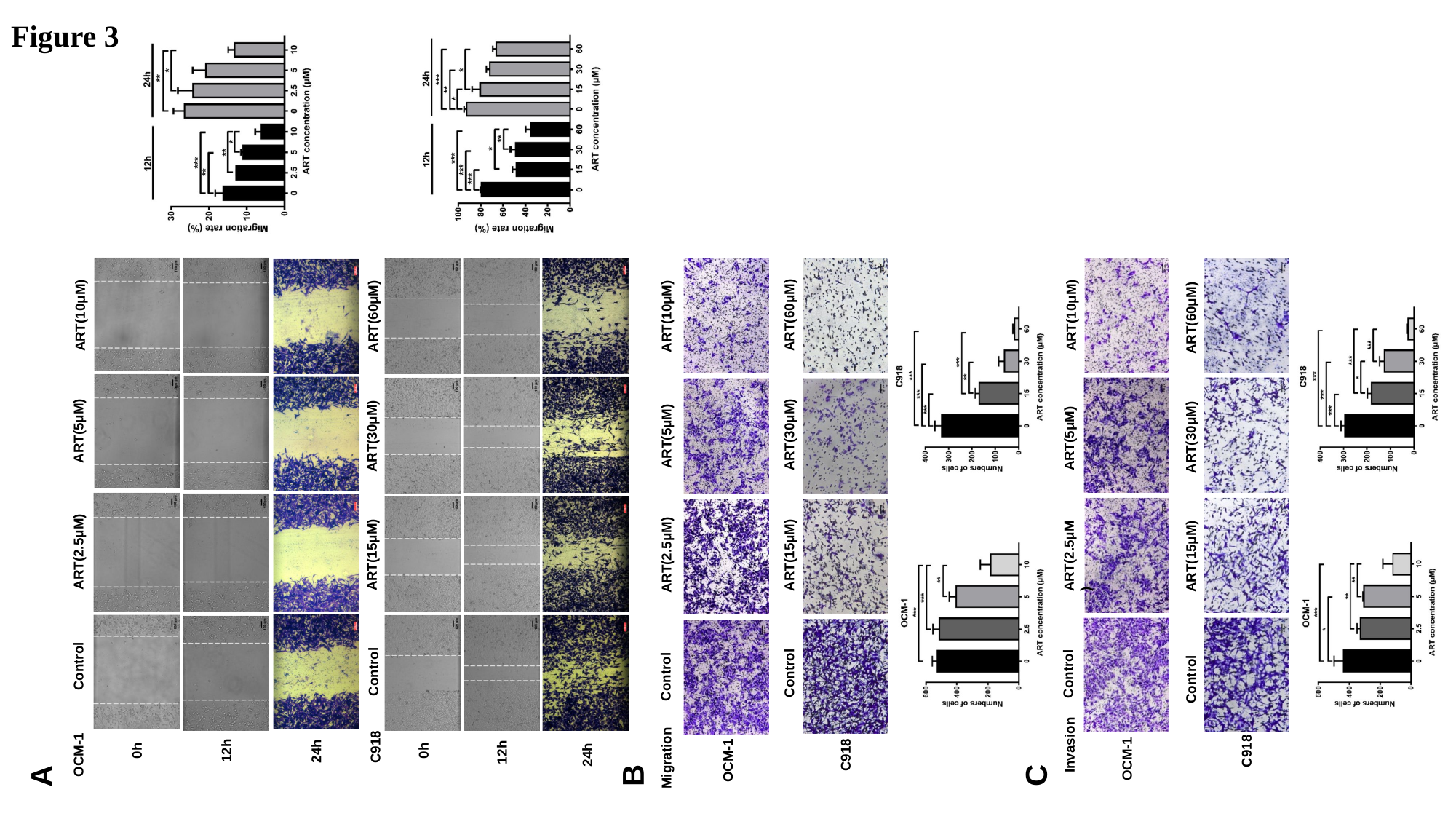

# Figure 3
ART(10μM)
ART(60μM)
ART(10μM)
ART(60μM)
ART(10μM)
ART(60μM)
ART(5μM)
ART(30μM)
ART(30μM)
ART(5μM)
ART(5μM)
ART(30μM)
ART(2.5μM)
ART(2.5μM)
ART(2.5μM)
ART(15μM)
ART(15μM)
ART(15μM)
Control
Control
Control
Control
Control
Control
Invasion
C918
0h
12h
12h
24h
C918
0h
OCM-1
C918
24h
OCM-1
Migration
OCM-1
B
C
A

## Slide 4
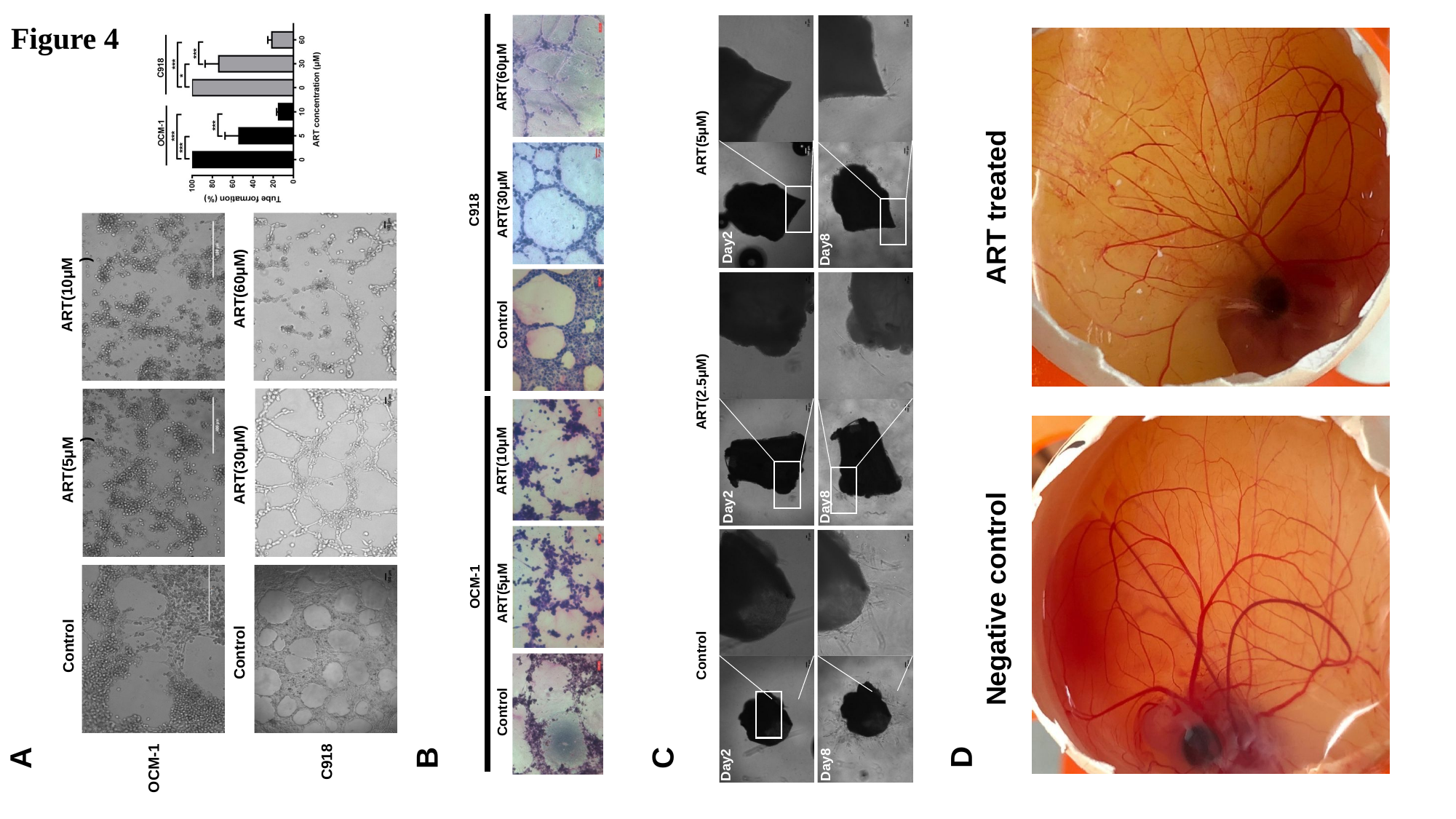

# Figure 4
ART(60μM)
ART(5μM)
ART treated
ART(30μM)
C918
Day2
Day8
Day2
Day8
ART(10μM)
ART(60μM)
Control
ART(2.5μM)
ART(10μM)
ART(5μM)
ART(30μM)
Day2
Day8
OCM-1
Negative control
ART(5μM)
Control
Control
Control
Control
Day8
A
B
C
D
C918
Day8
Day2
OCM-1

## Slide 5
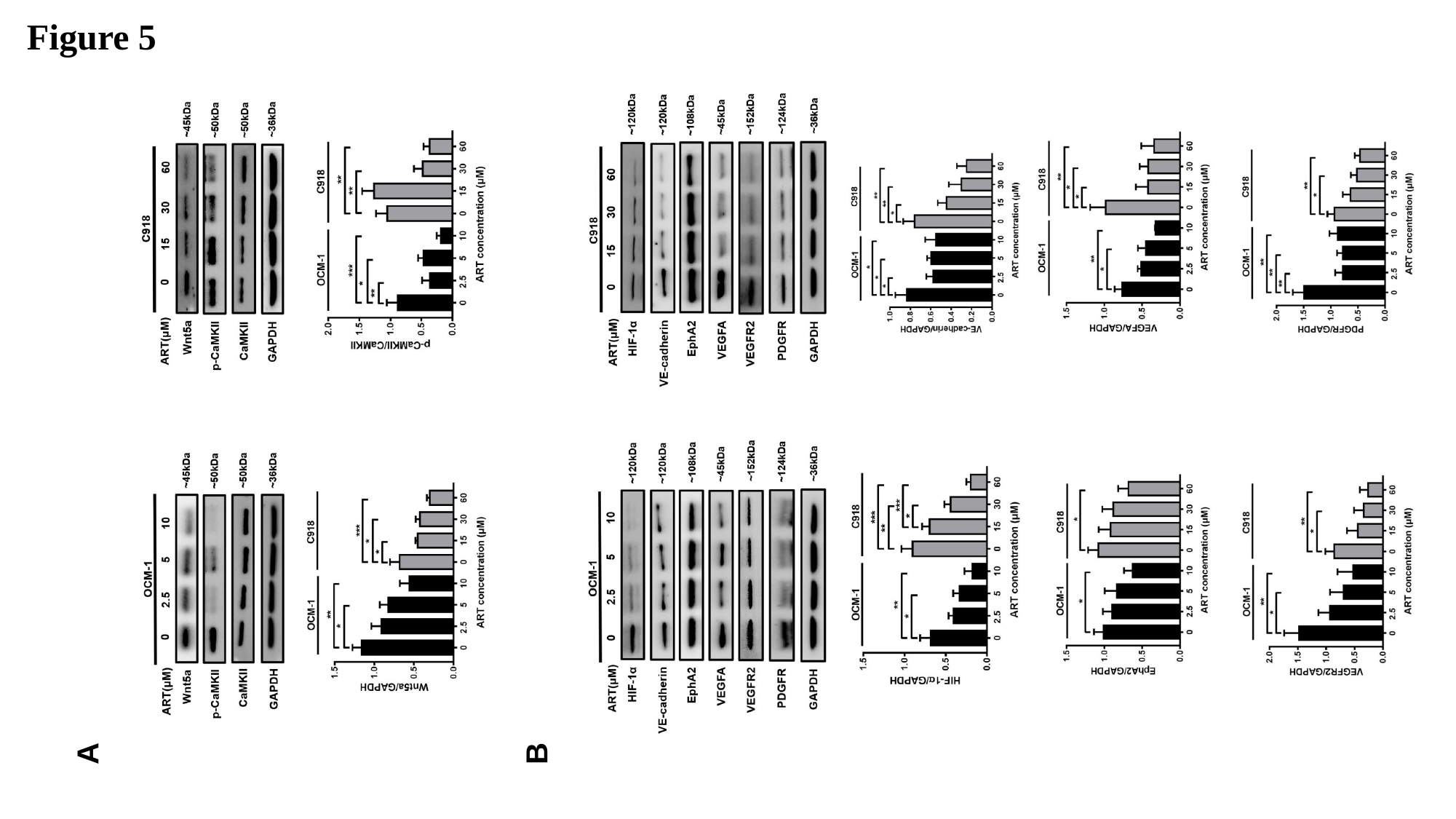

# Figure 5
A
B

## Slide 6
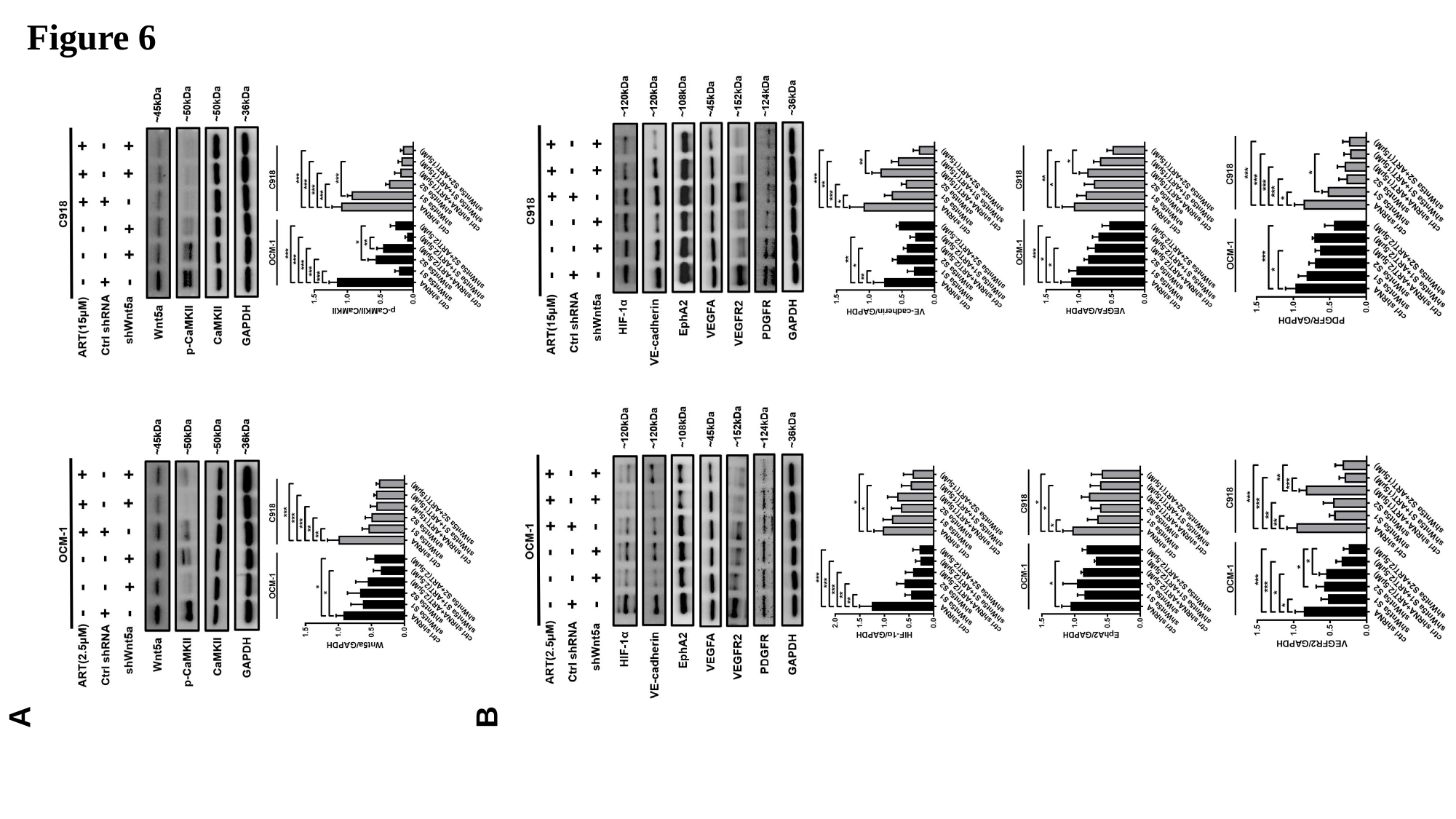

# Figure 6
A
B

## Slide 7
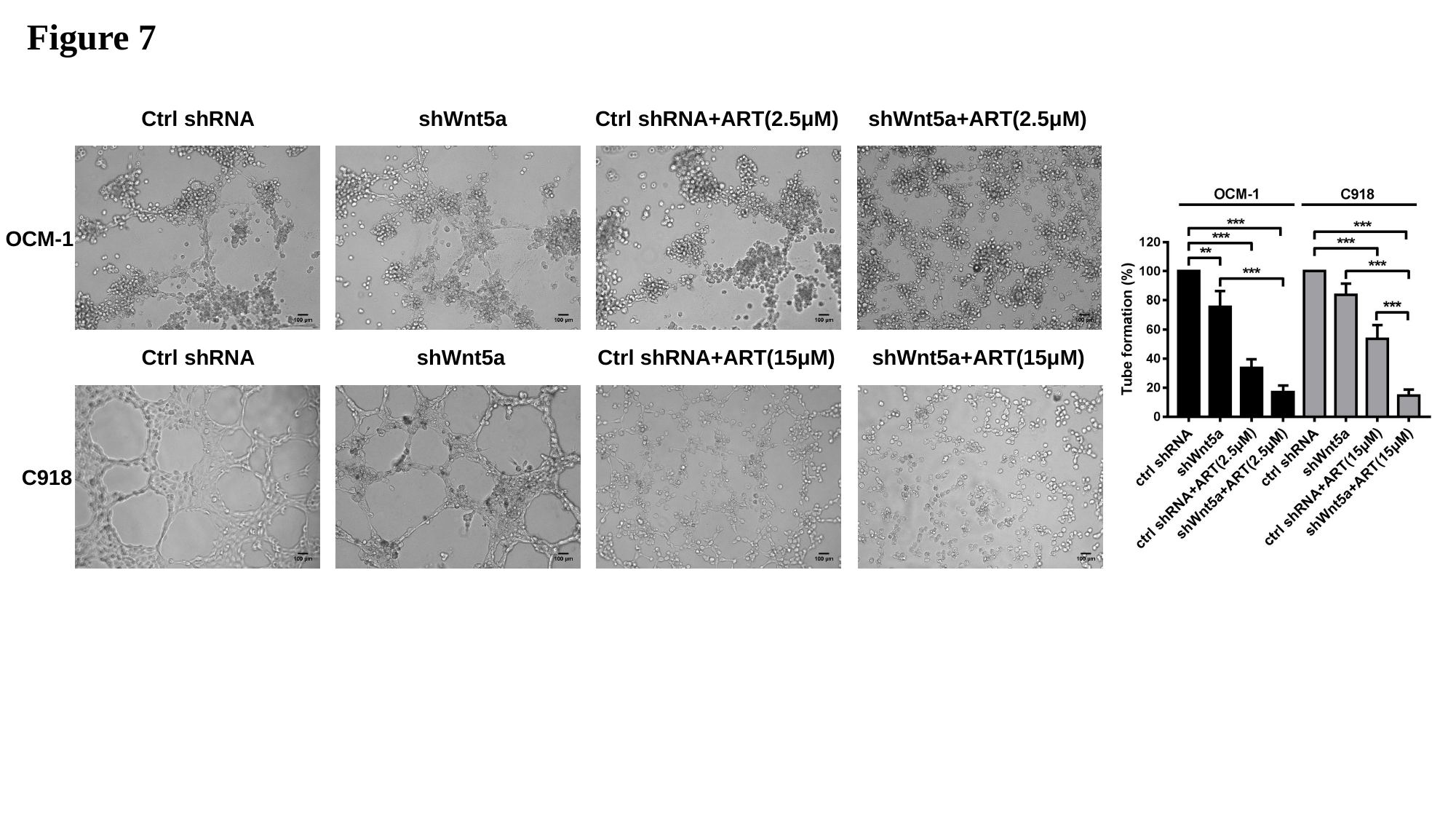

# Figure 7
Ctrl shRNA
shWnt5a
Ctrl shRNA+ART(2.5μM)
shWnt5a+ART(2.5μM)
OCM-1
Ctrl shRNA
shWnt5a
Ctrl shRNA+ART(15μM)
shWnt5a+ART(15μM)
C918

## Slide 8
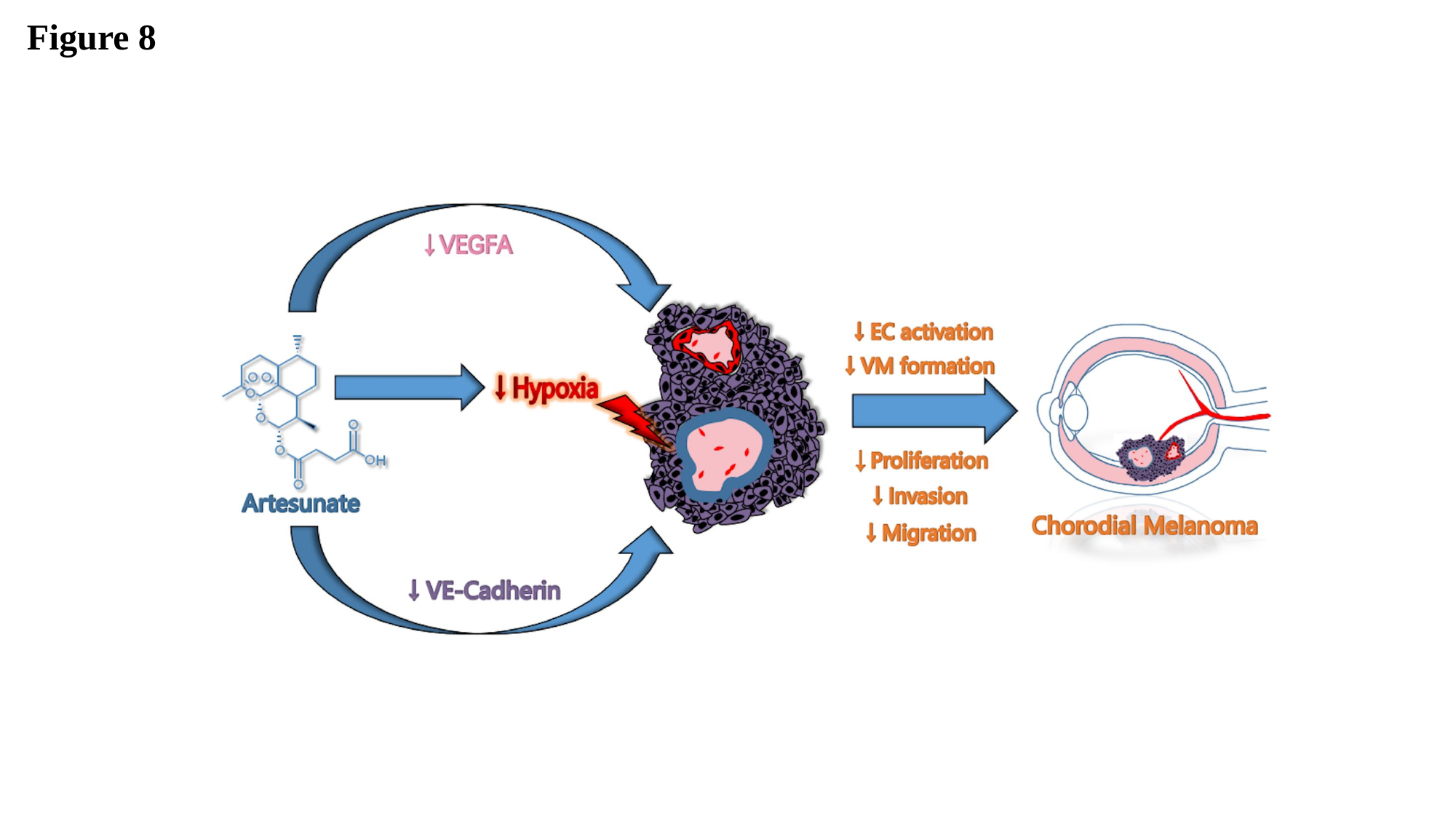

# Figure 8

## Slide 9
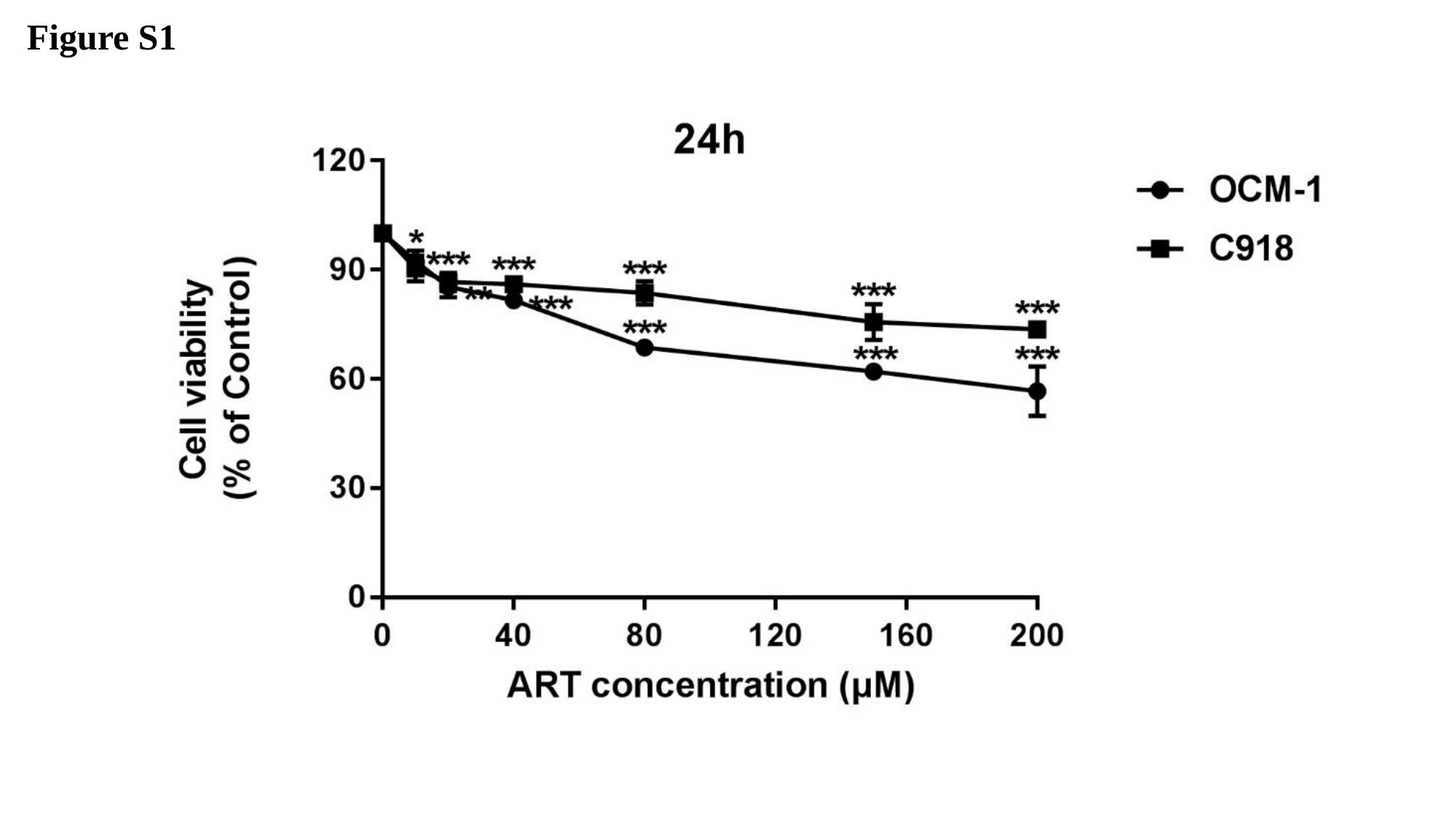

# Figure S1

## Slide 10
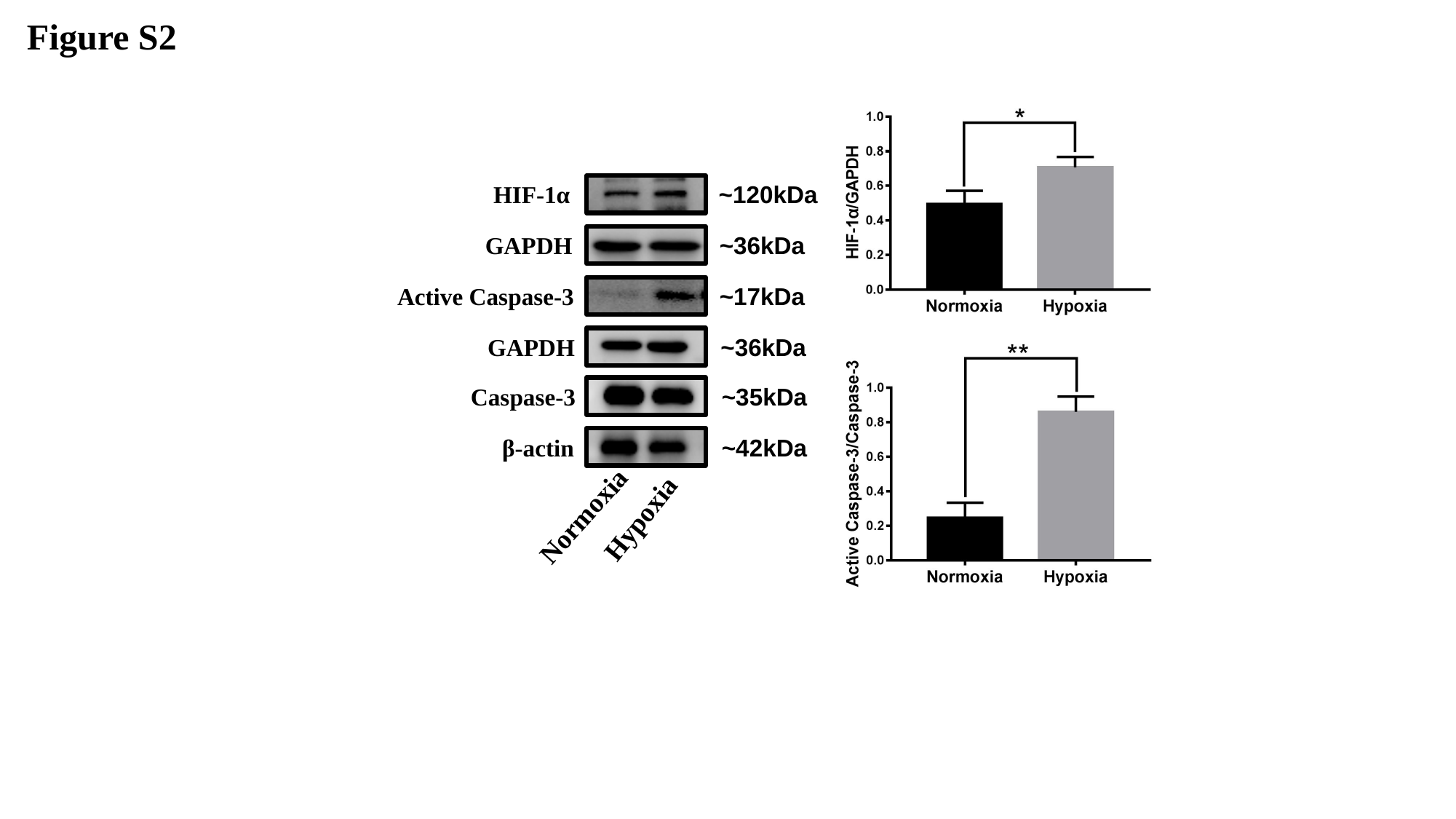

# Figure S2
~120kDa
HIF-1α
GAPDH
~36kDa
Active Caspase-3
~17kDa
~36kDa
GAPDH
Caspase-3
~35kDa
β-actin
~42kDa
Hypoxia
Normoxia

## Slide 11
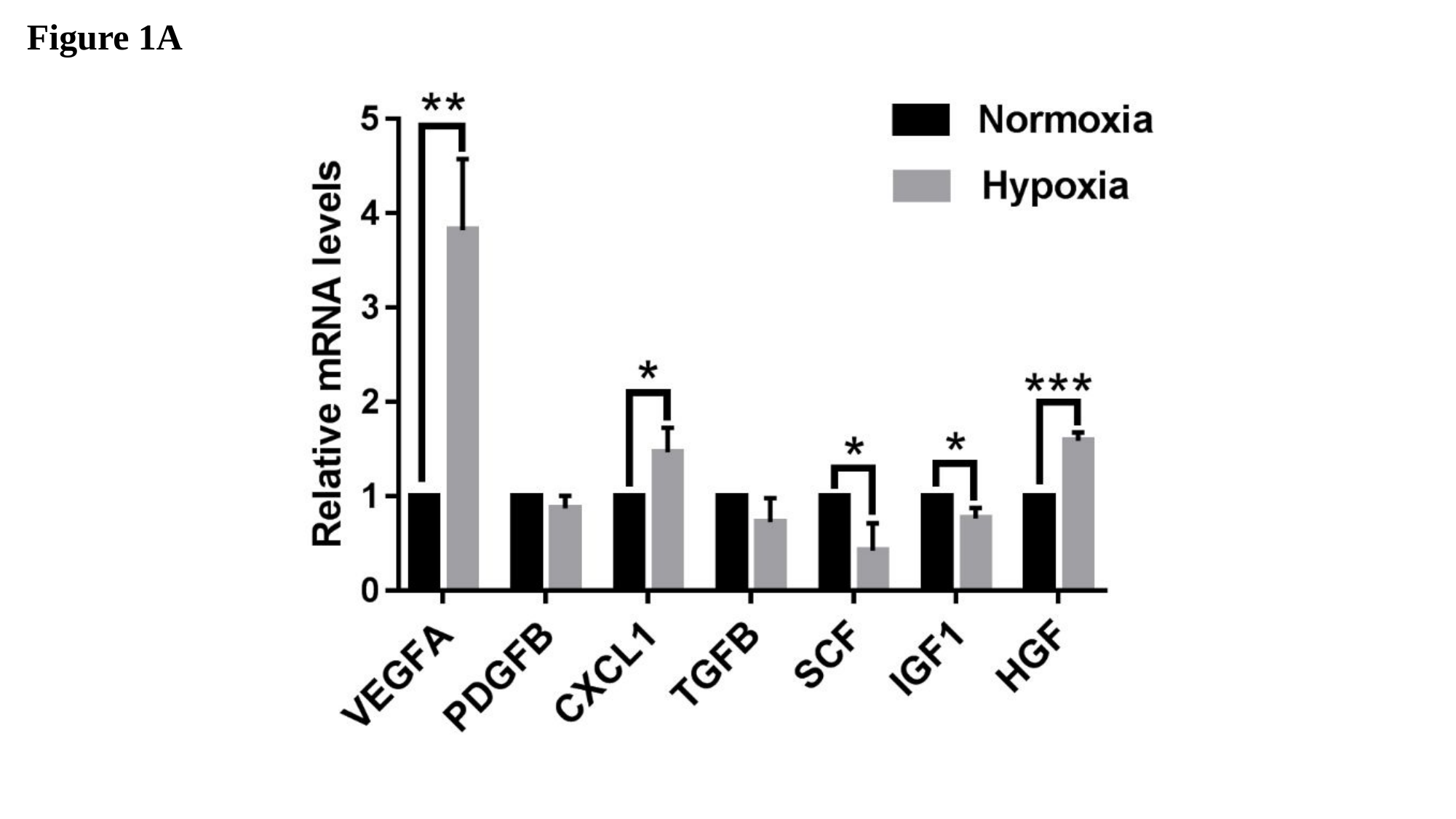

# Figure 1A

## Slide 12
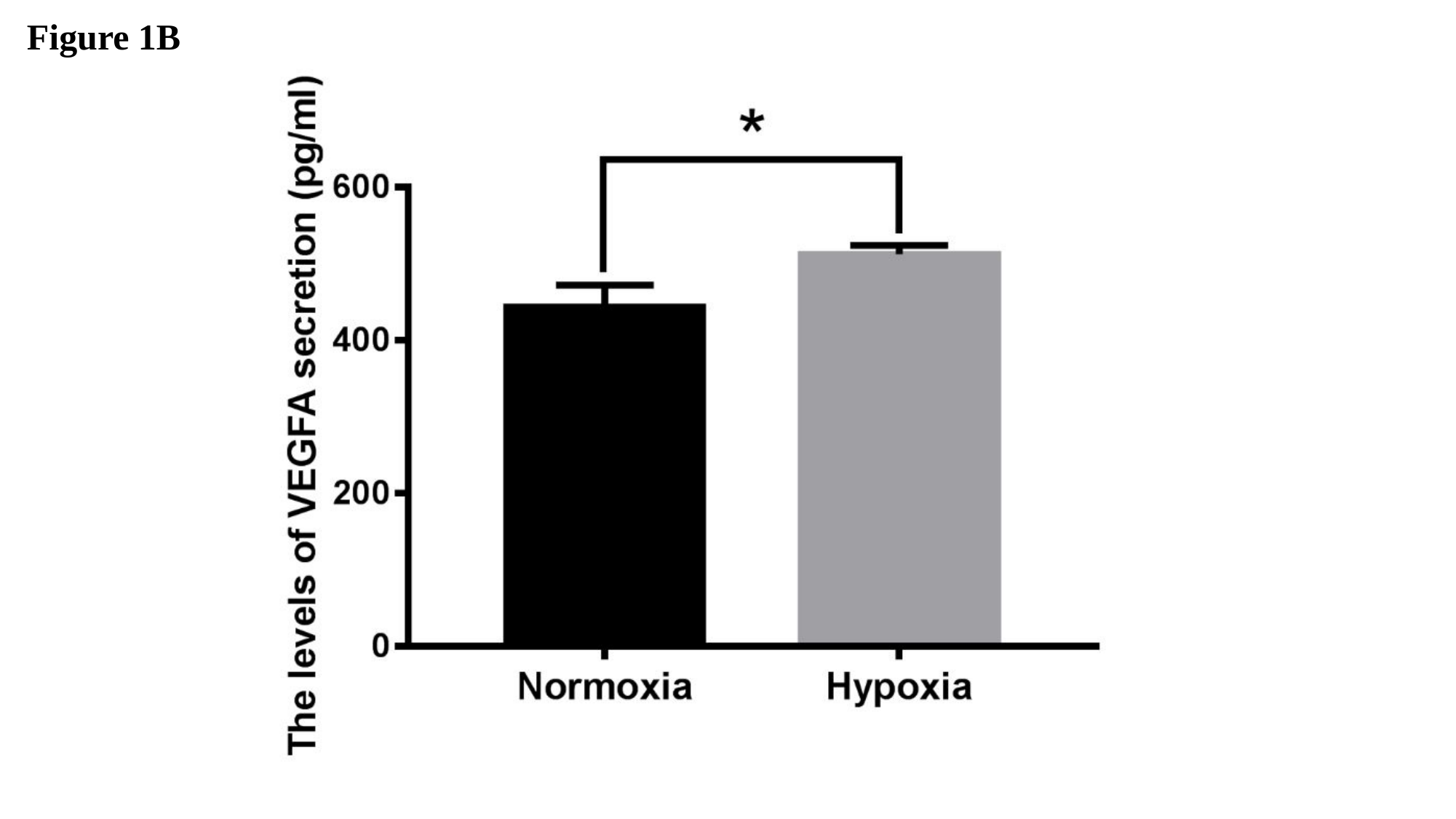

# Figure 1B

## Slide 13
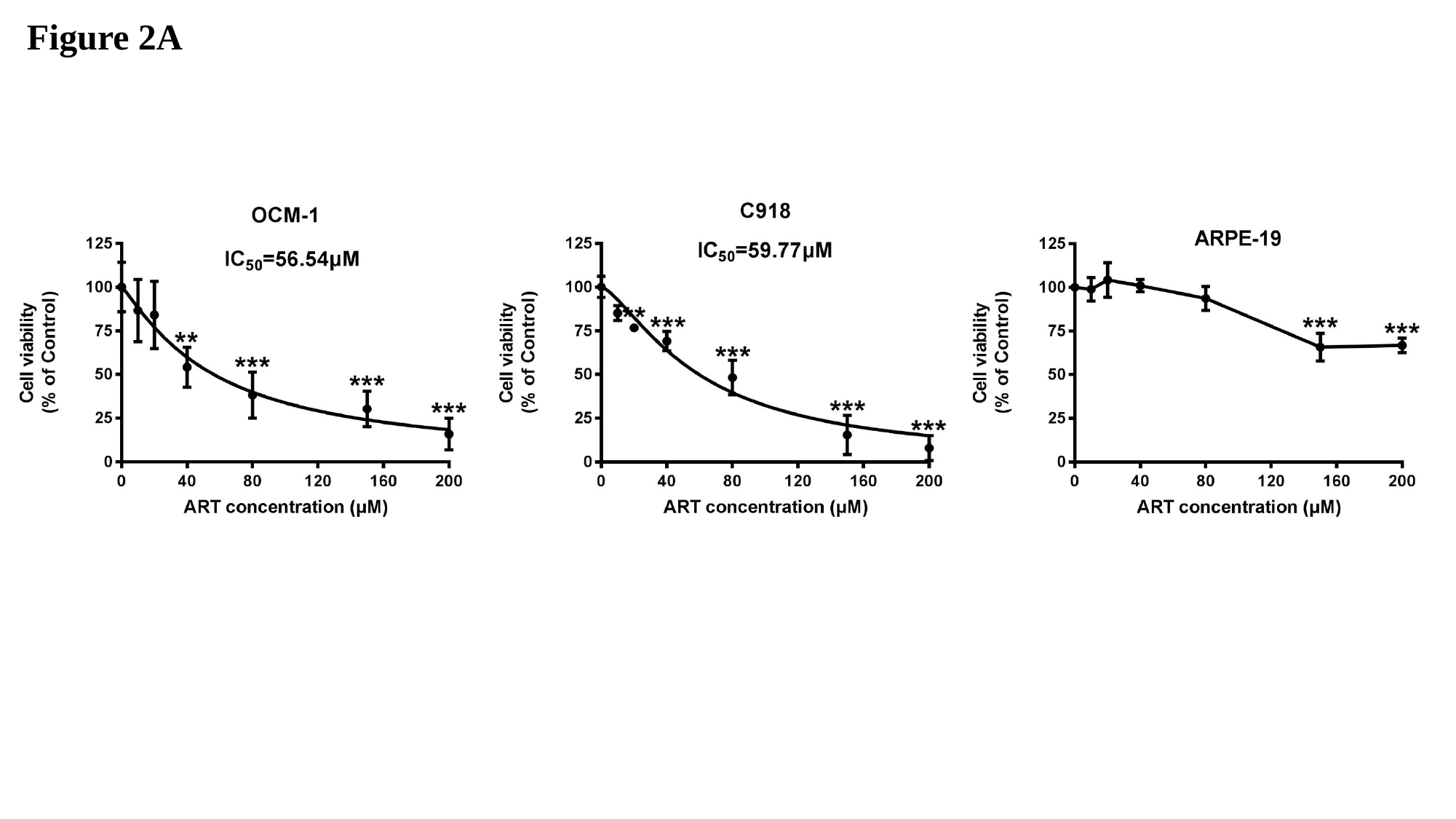

# Figure 2A

## Slide 14
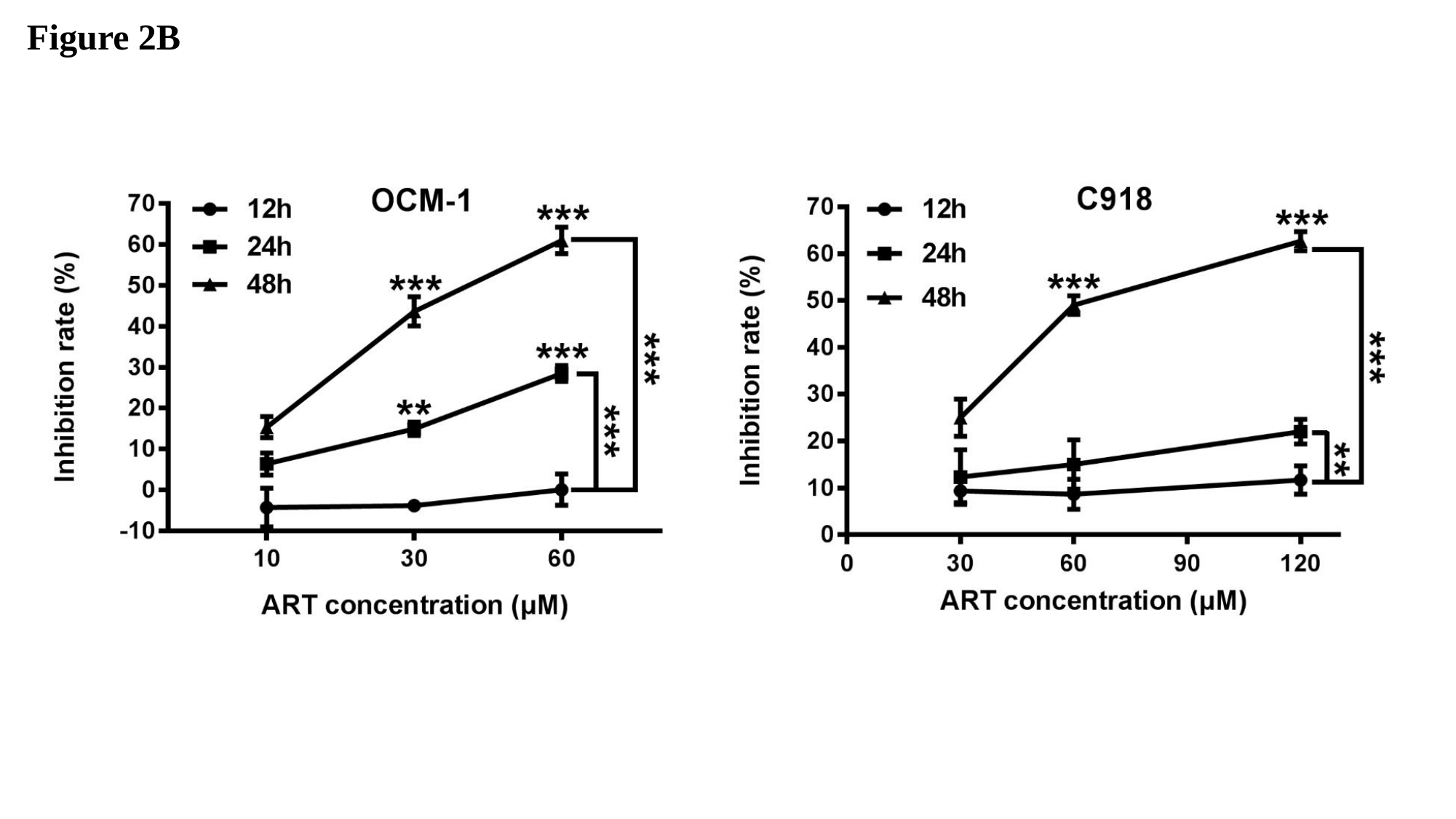

# Figure 2B

## Slide 15
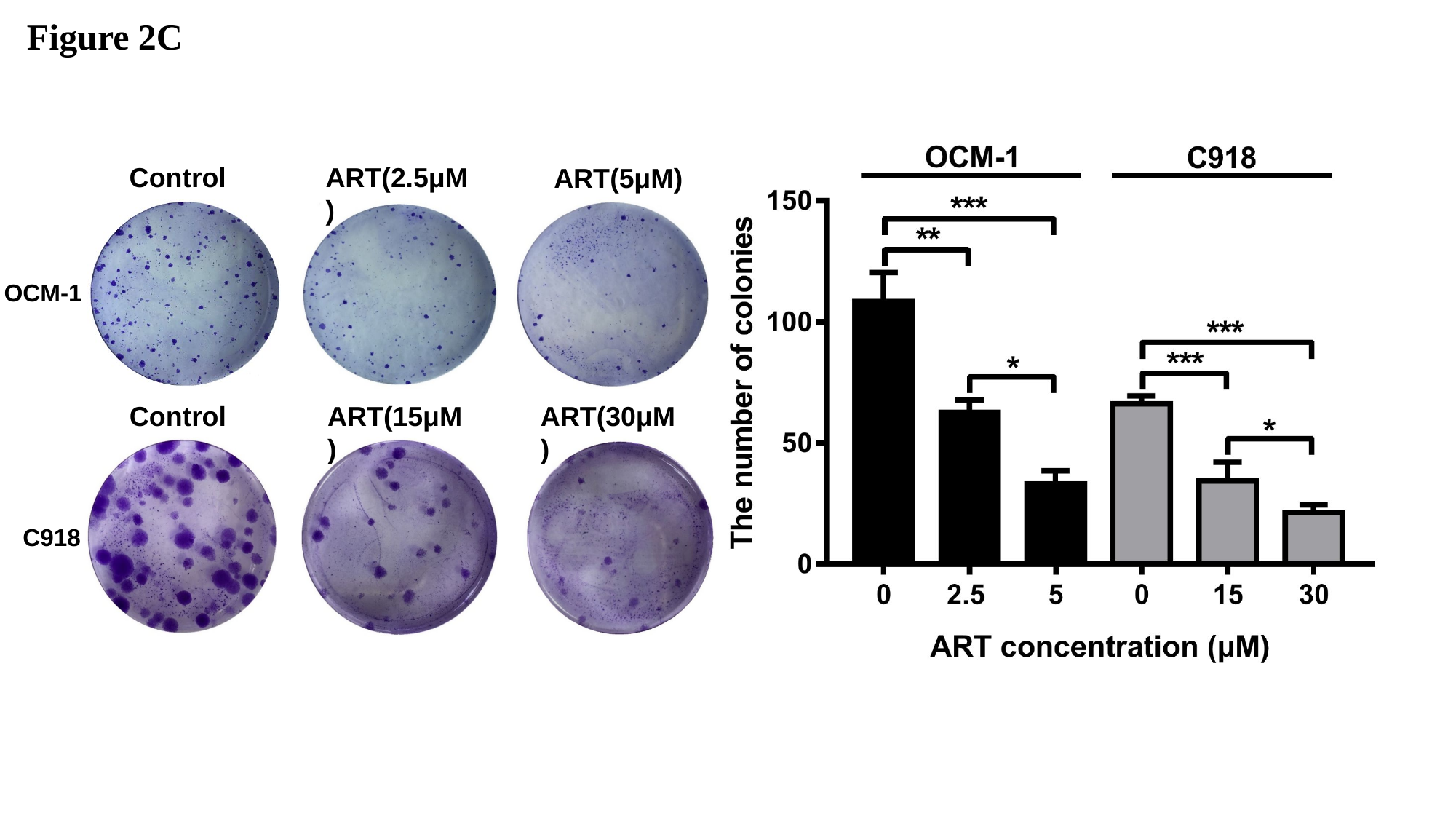

# Figure 2C
Control
ART(2.5μM)
ART(5μM)
OCM-1
ART(15μM)
ART(30μM)
Control
C918

## Slide 16
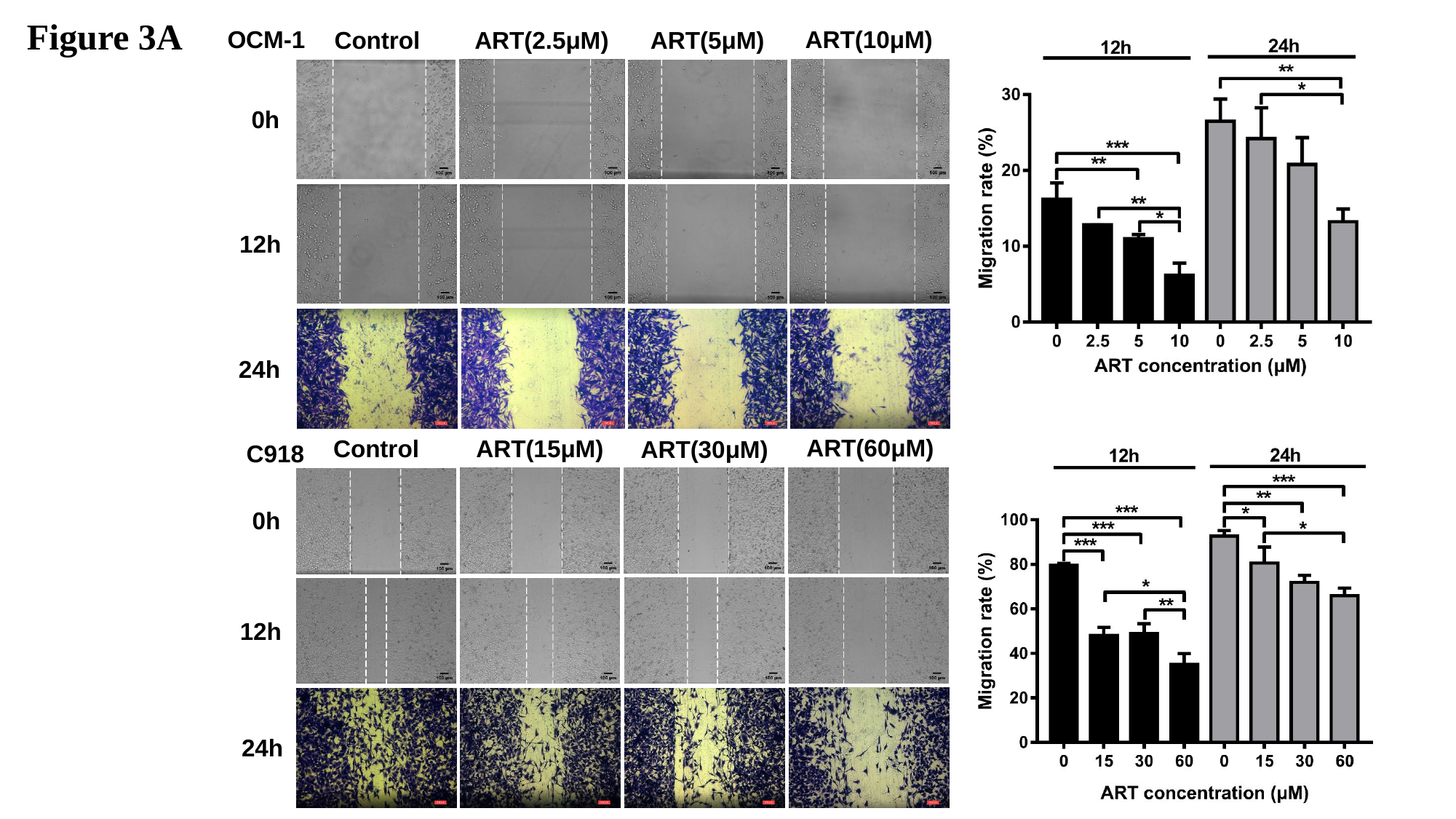

# Figure 3A
OCM-1
ART(10μM)
Control
ART(2.5μM)
ART(5μM)
0h
12h
24h
ART(60μM)
Control
ART(15μM)
ART(30μM)
C918
0h
12h
24h
c

## Slide 17
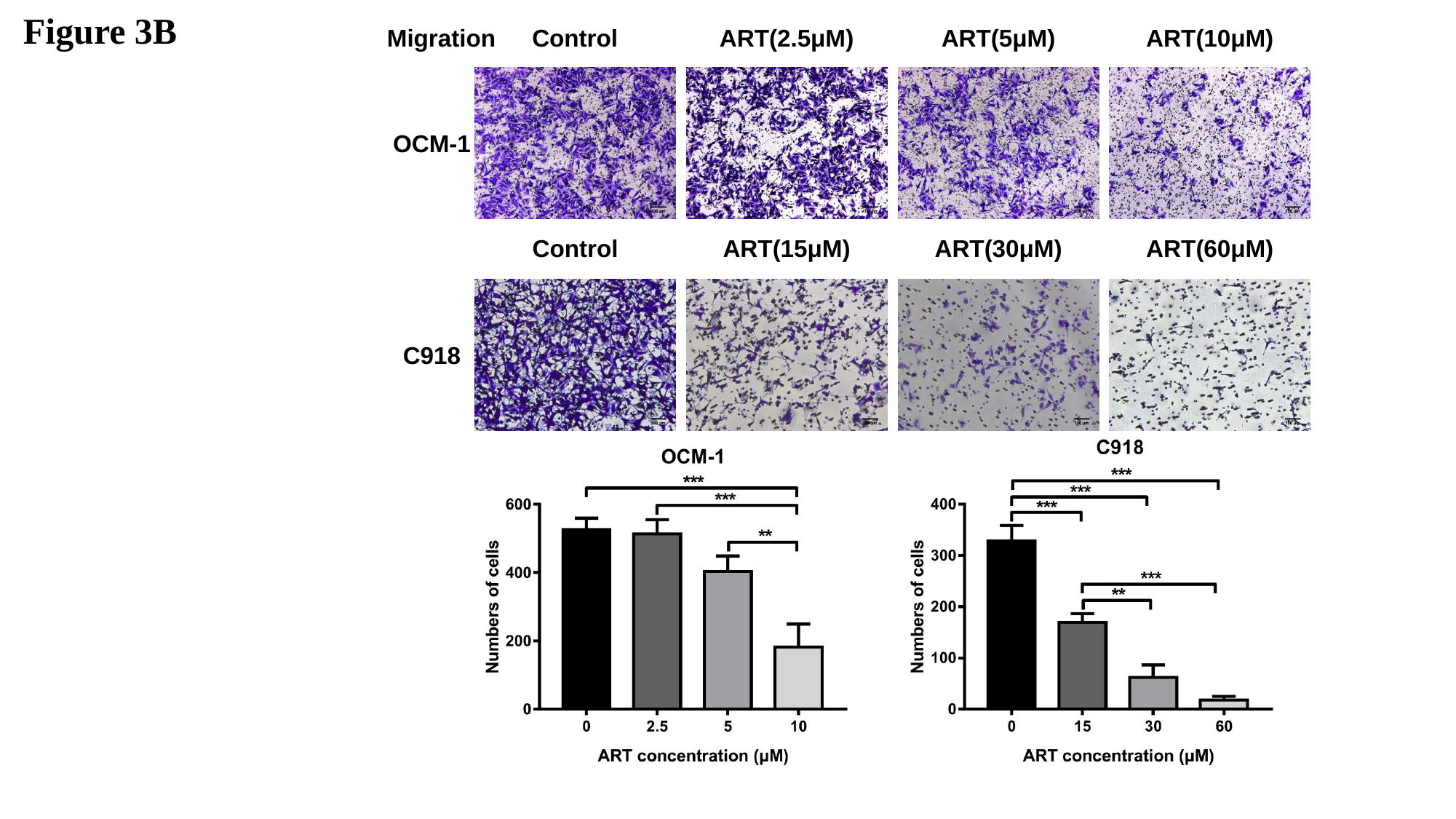

# Figure 3B
Migration
ART(10μM)
Control
ART(2.5μM)
ART(5μM)
OCM-1
ART(60μM)
ART(15μM)
Control
ART(30μM)
C918

## Slide 18
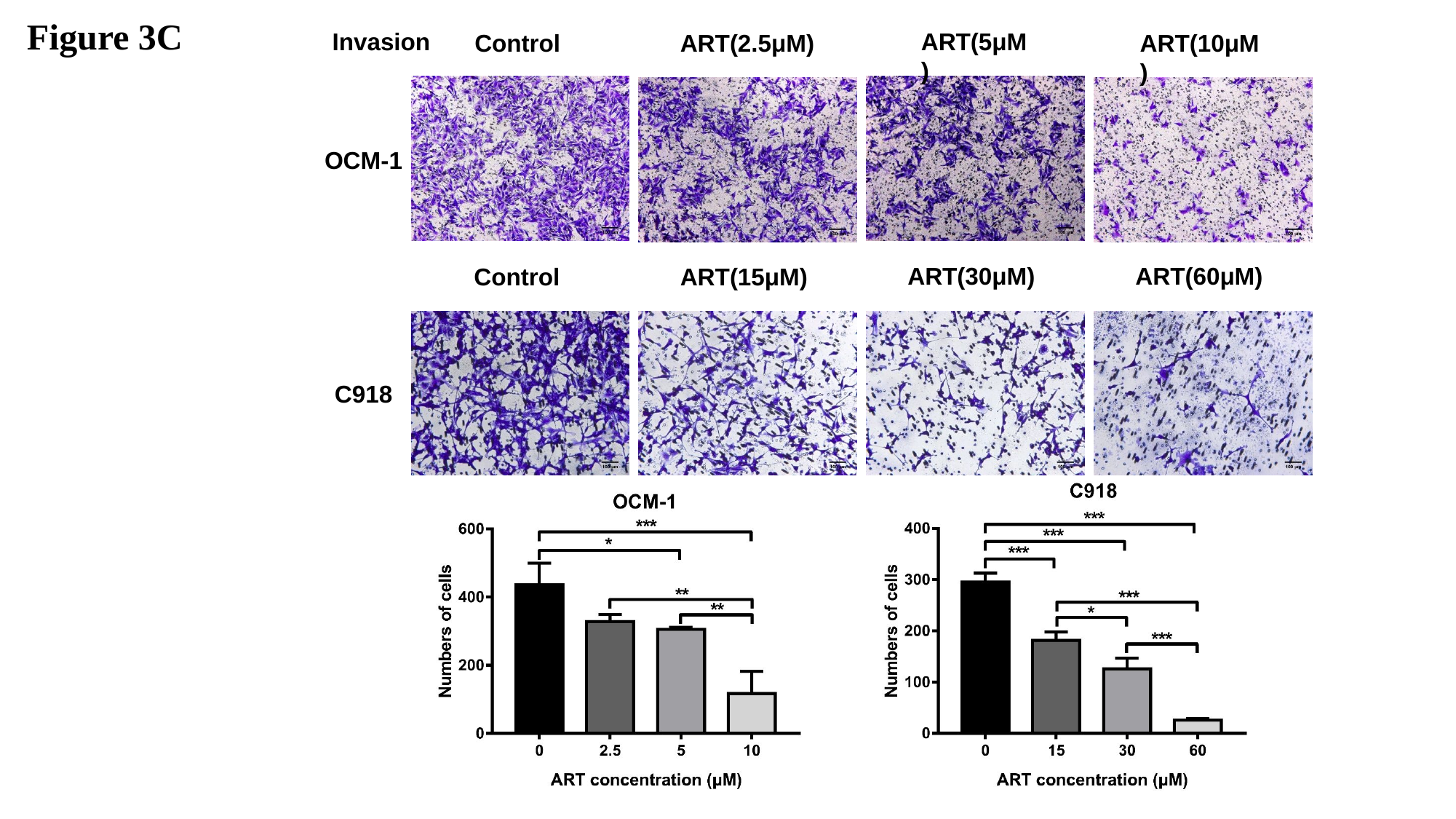

# Figure 3C
Invasion
ART(5μM)
Control
ART(10μM)
ART(2.5μM)
OCM-1
ART(30μM)
ART(60μM)
ART(15μM)
Control
C918

## Slide 19
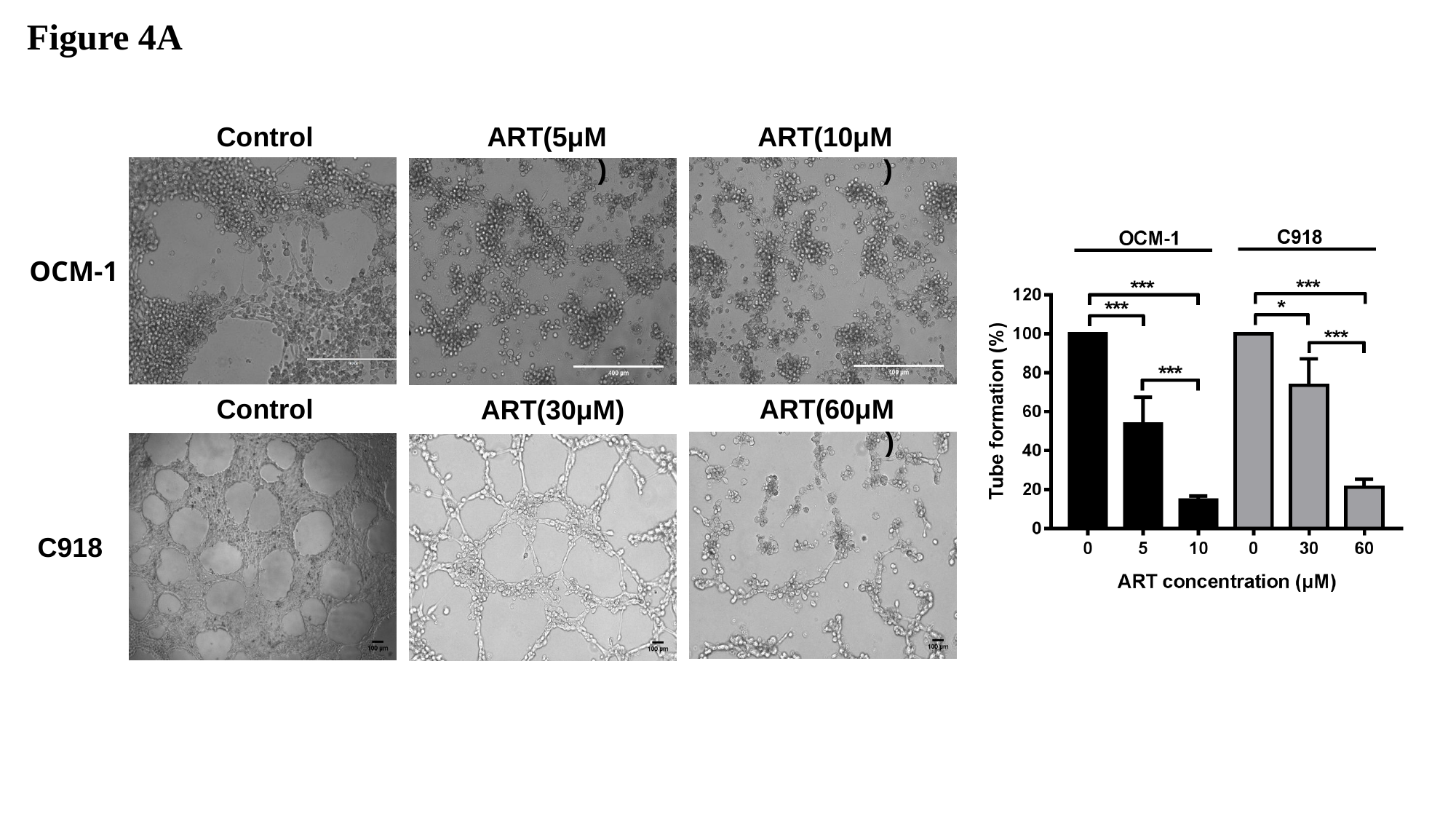

# Figure 4A
Control
ART(5μM)
ART(10μM)
OCM-1
ART(60μM)
Control
ART(30μM)
C918

## Slide 20
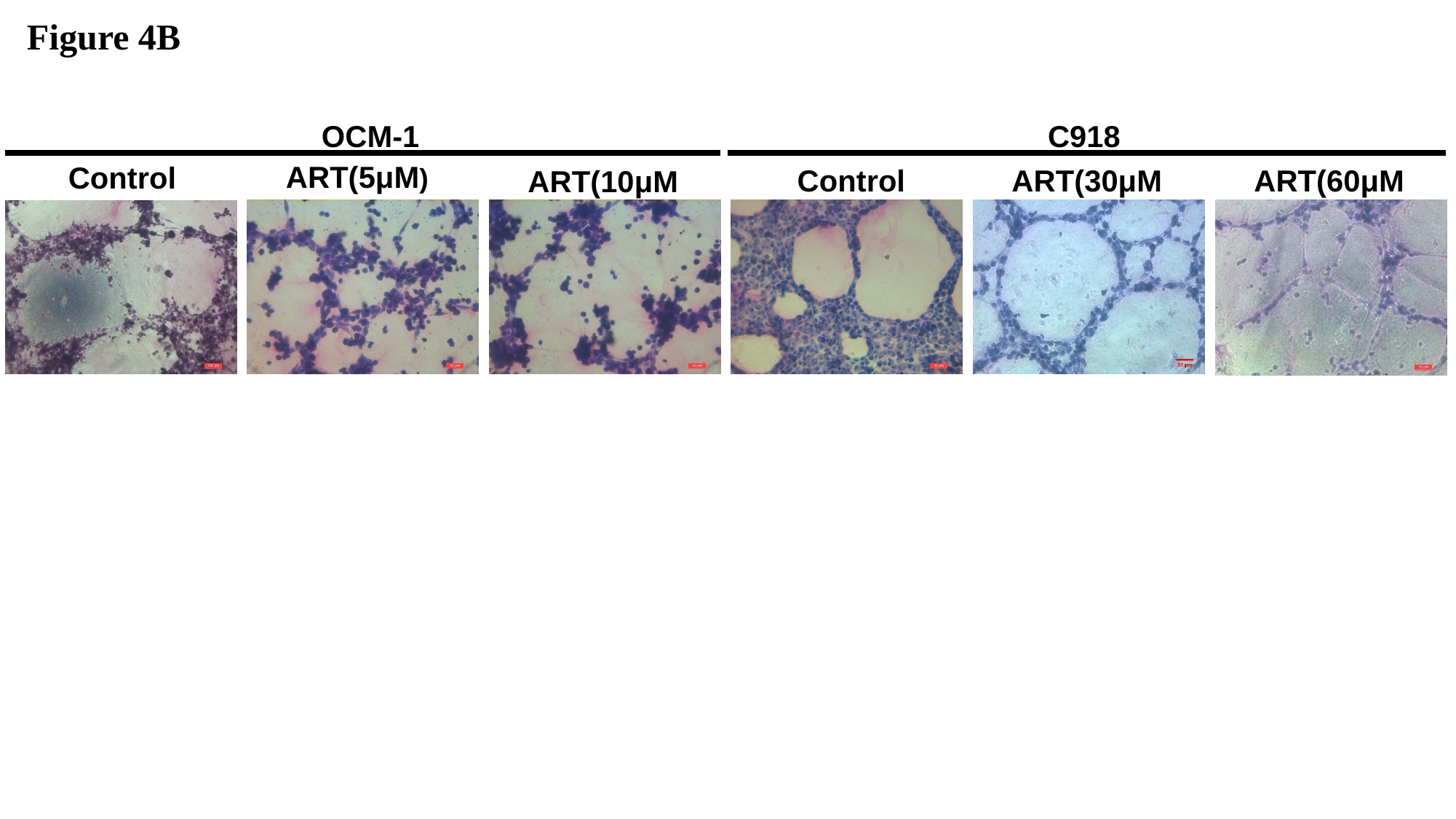

# Figure 4B
OCM-1
C918
ART(5μM)
Control
ART(30μM)
Control
ART(60μM)
ART(10μM)

## Slide 21
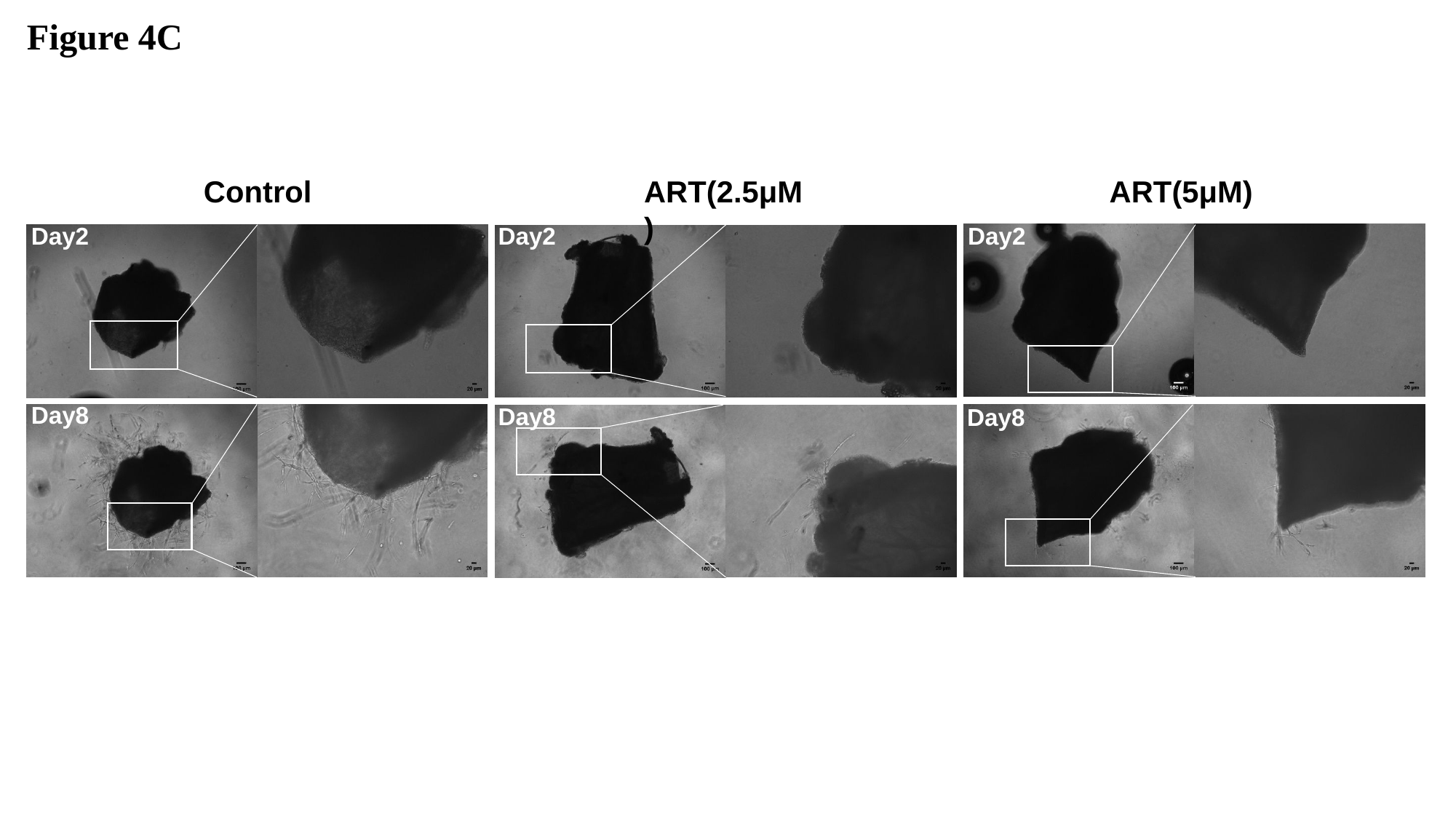

# Figure 4C
ART(5μM)
Control
ART(2.5μM)
Day2
Day2
Day2
Day2
Day8
Day8
Day8
Day8
Day8

## Slide 22
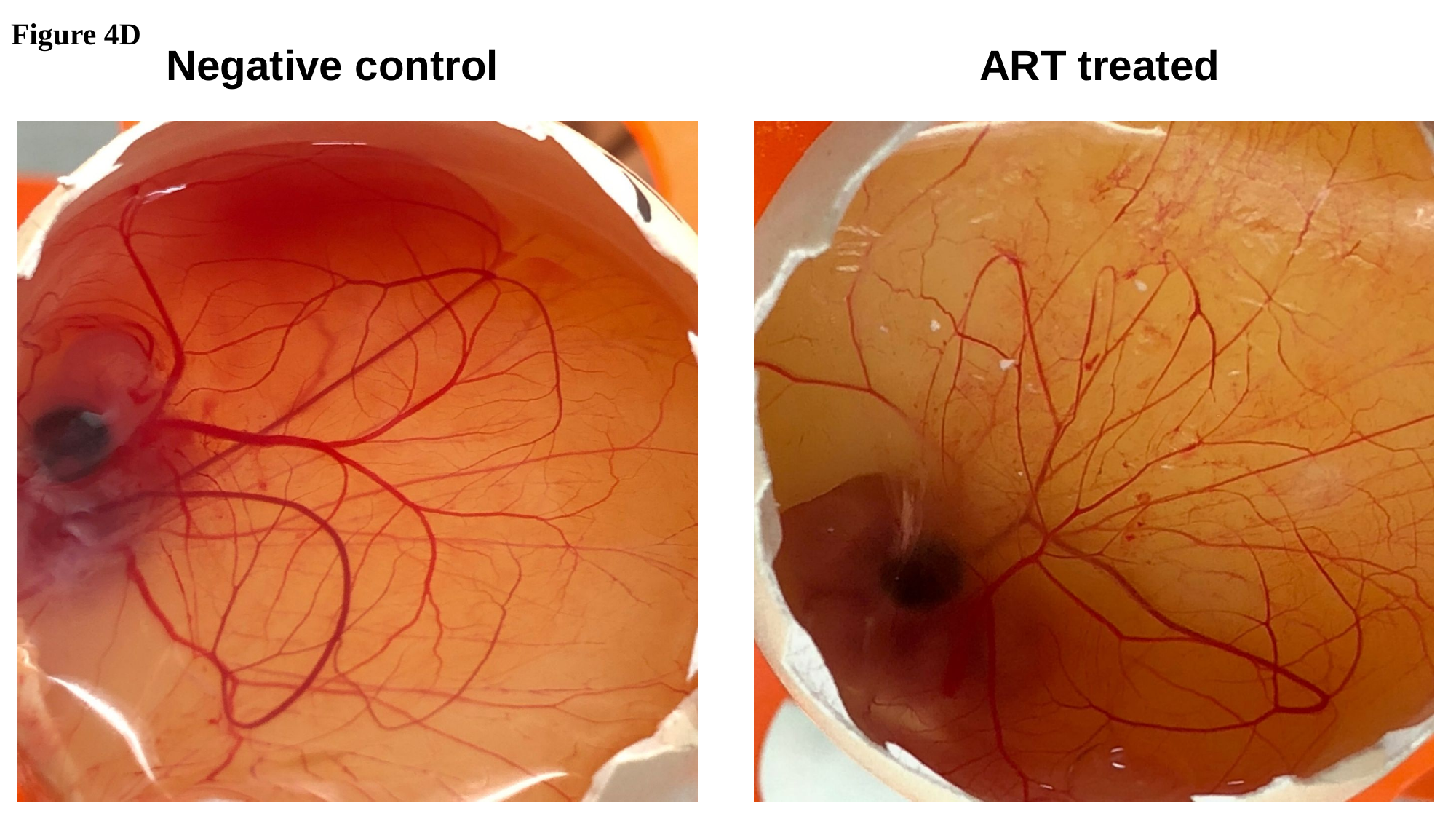

# Figure 4D
Negative control
ART treated

## Slide 23
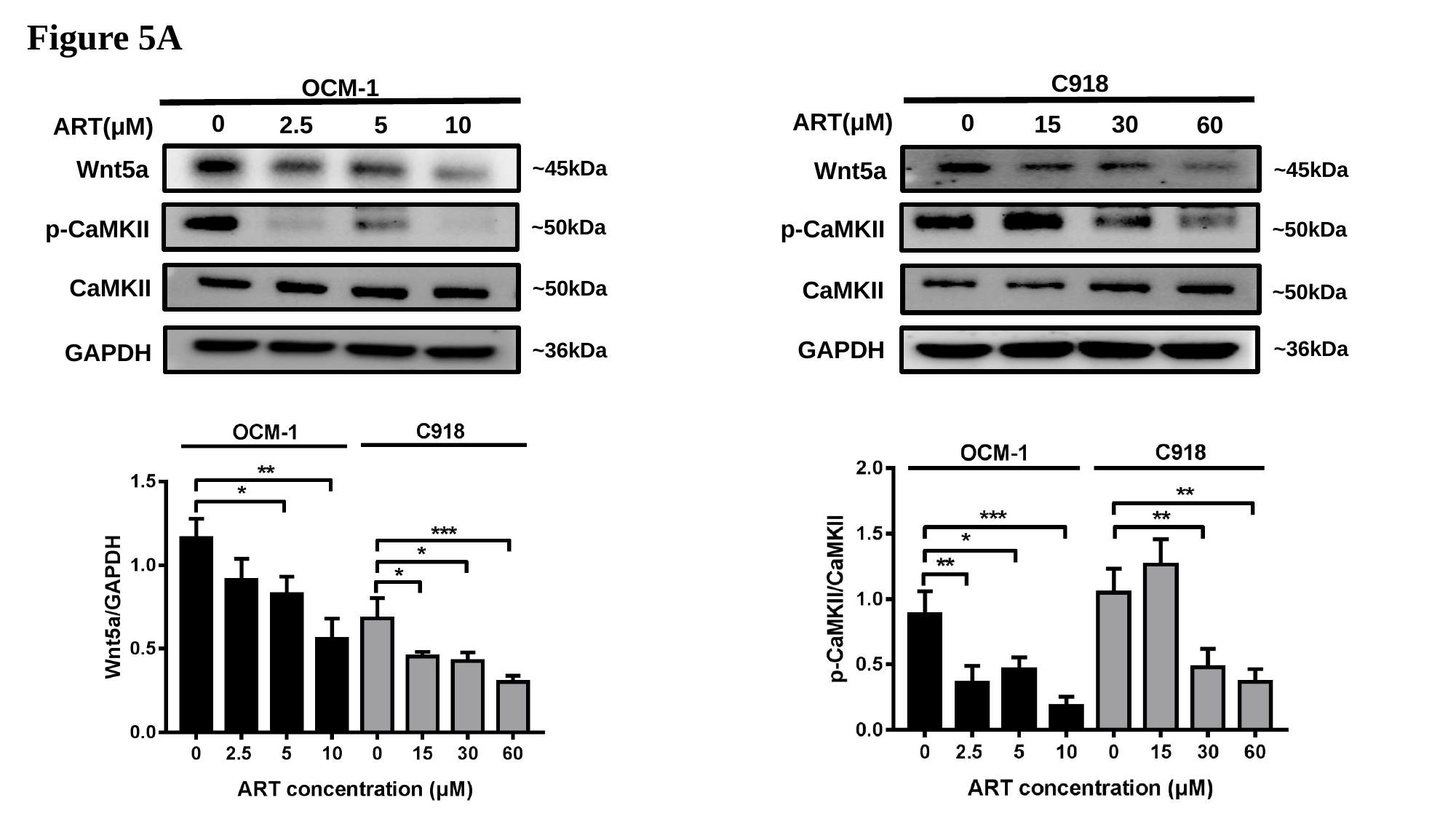

# Figure 5A
C918
OCM-1
ART(μM)
0
0
15
30
5
10
2.5
60
ART(μM)
Wnt5a
~45kDa
Wnt5a
~45kDa
p-CaMKII
p-CaMKII
~50kDa
~50kDa
CaMKII
CaMKII
~50kDa
~50kDa
GAPDH
~36kDa
~36kDa
GAPDH

## Slide 24
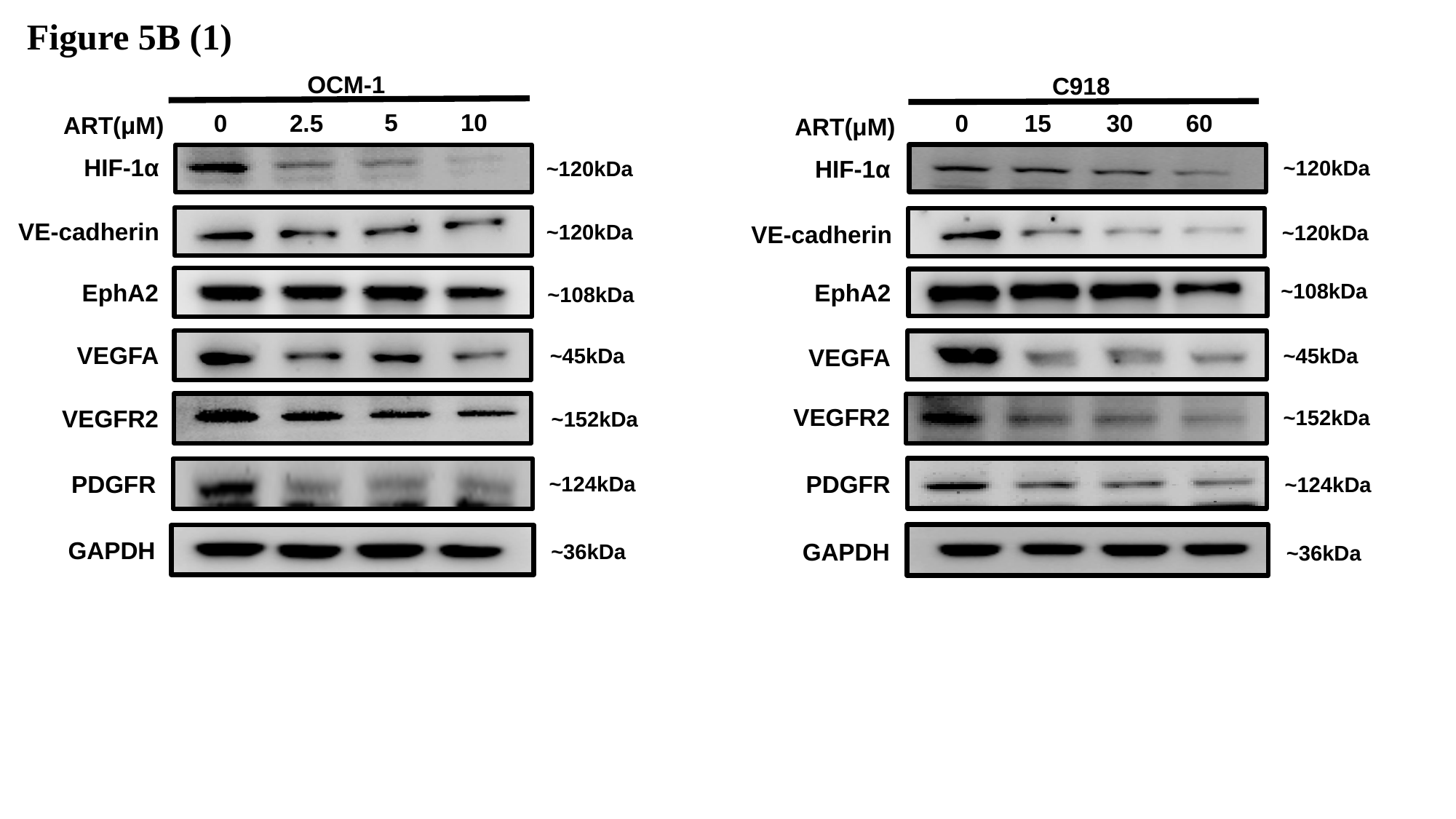

# Figure 5B (1)
OCM-1
C918
5
10
60
30
0
0
2.5
15
ART(μM)
ART(μM)
HIF-1α
HIF-1α
~120kDa
~120kDa
VE-cadherin
~120kDa
VE-cadherin
~120kDa
EphA2
EphA2
~108kDa
~108kDa
VEGFA
VEGFA
~45kDa
~45kDa
VEGFR2
VEGFR2
~152kDa
~152kDa
PDGFR
PDGFR
~124kDa
~124kDa
GAPDH
GAPDH
~36kDa
~36kDa

## Slide 25
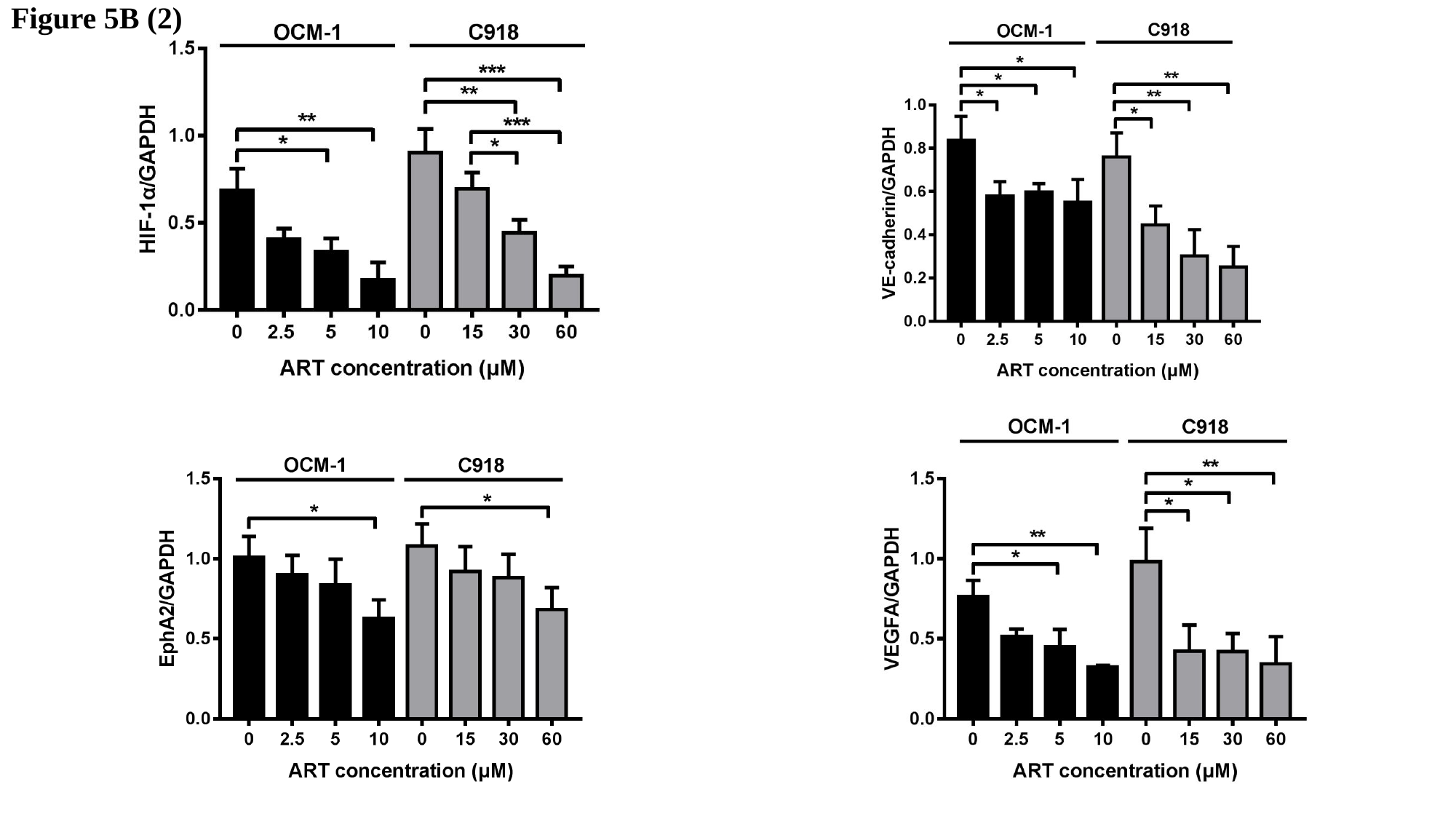

# Figure 5B (2)

## Slide 26
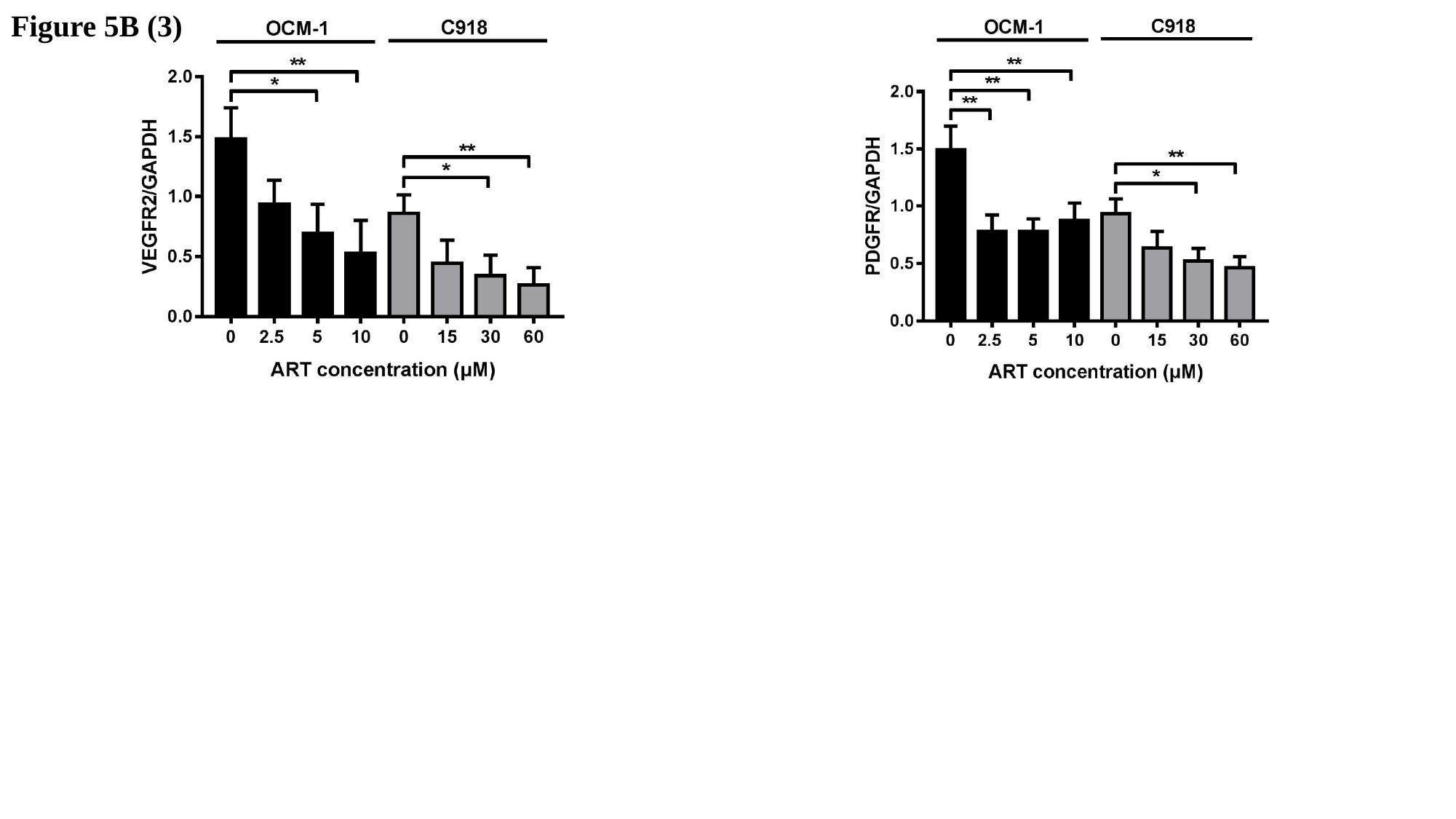

# Figure 5B (3)

## Slide 27
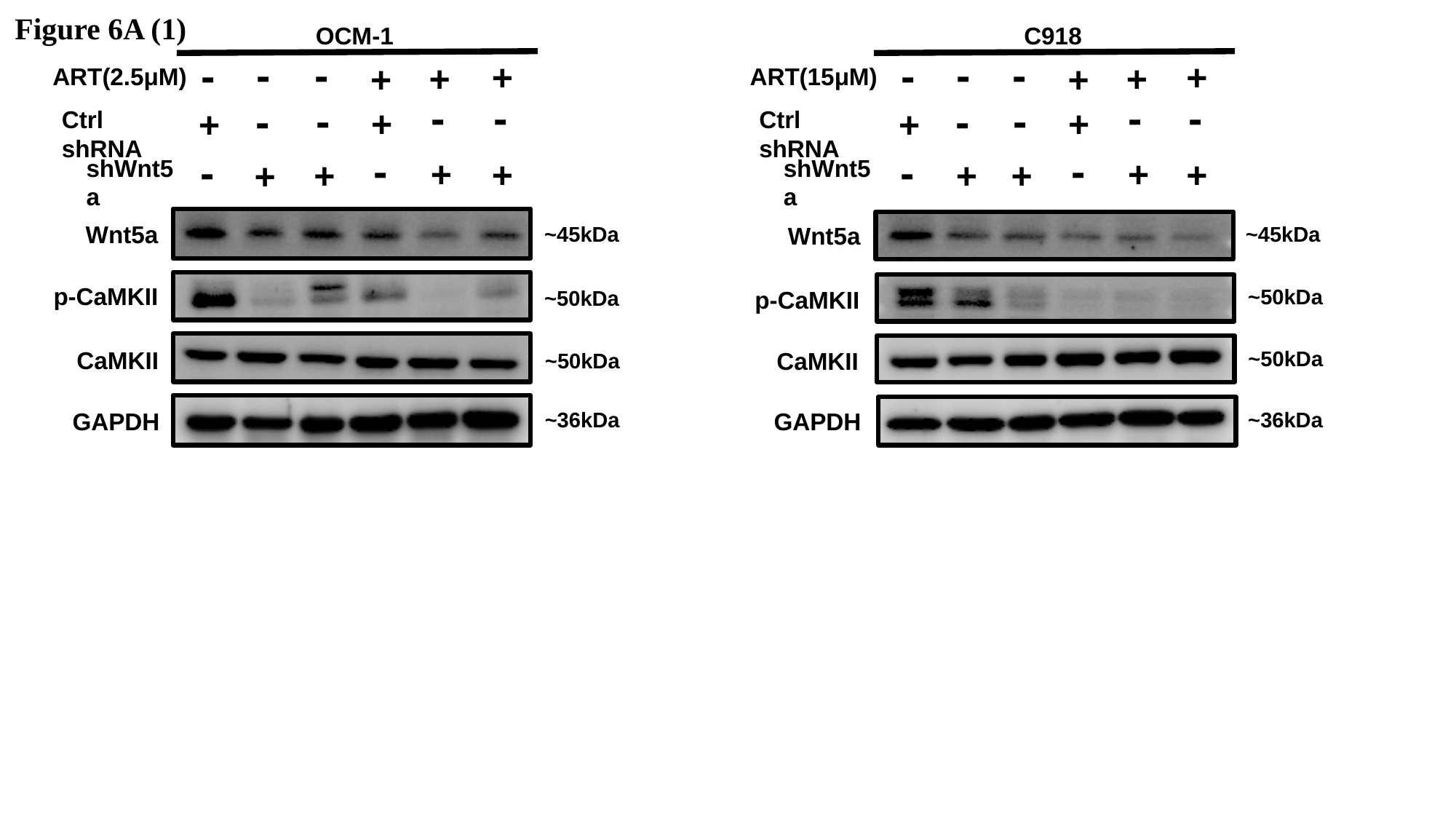

# Figure 6A (1)
OCM-1
-
-
-
+
Ctrl shRNA
+
+
ART(2.5μM)
-
-
-
-
+
+
shWnt5a
-
-
+
+
+
+
C918
-
-
-
+
Ctrl shRNA
+
+
ART(15μM)
-
-
-
-
+
+
shWnt5a
-
-
+
+
+
+
Wnt5a
~45kDa
~45kDa
Wnt5a
p-CaMKII
~50kDa
p-CaMKII
~50kDa
CaMKII
~50kDa
CaMKII
~50kDa
~36kDa
GAPDH
~36kDa
GAPDH

## Slide 28
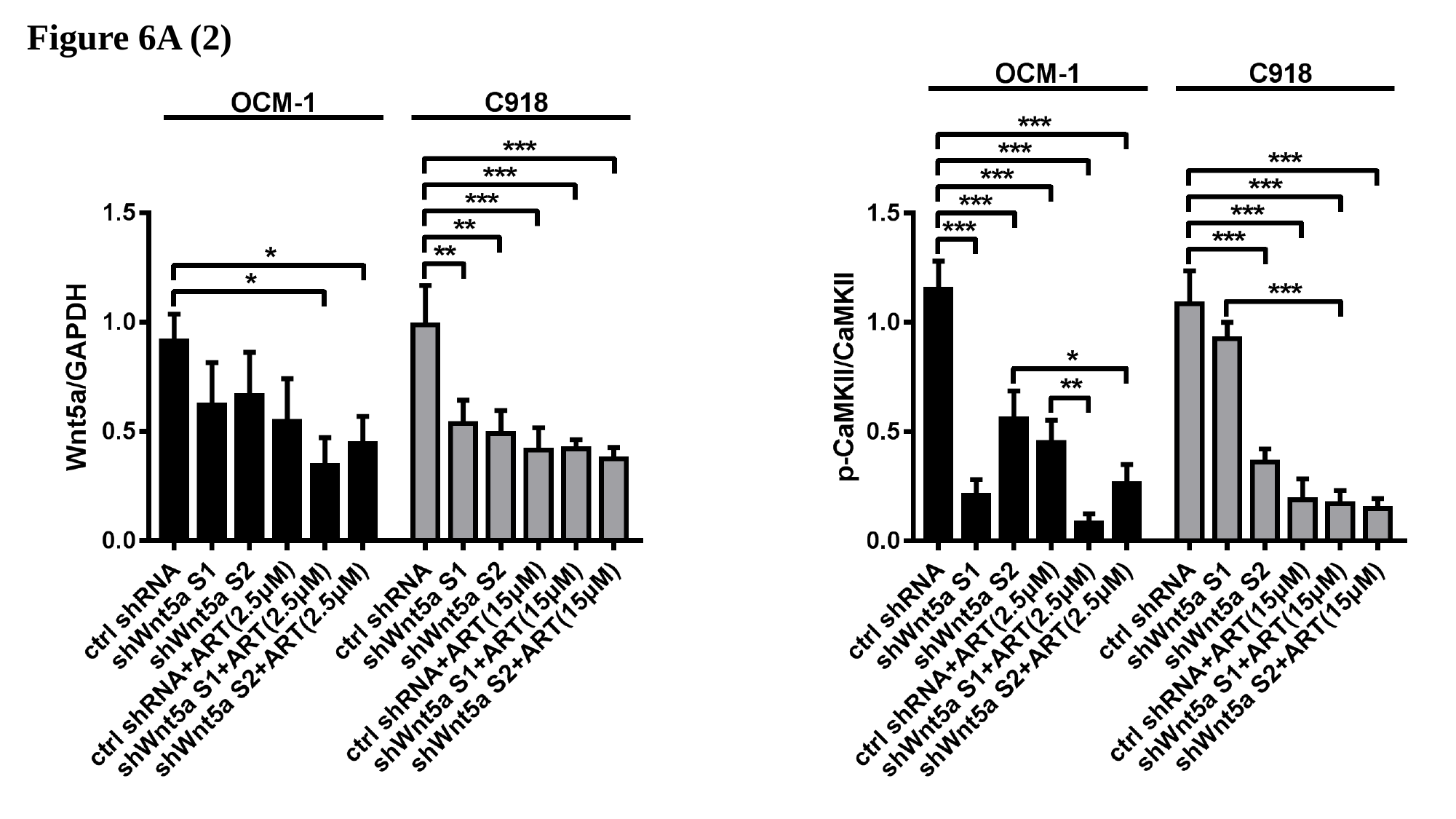

# Figure 6A (2)

## Slide 29
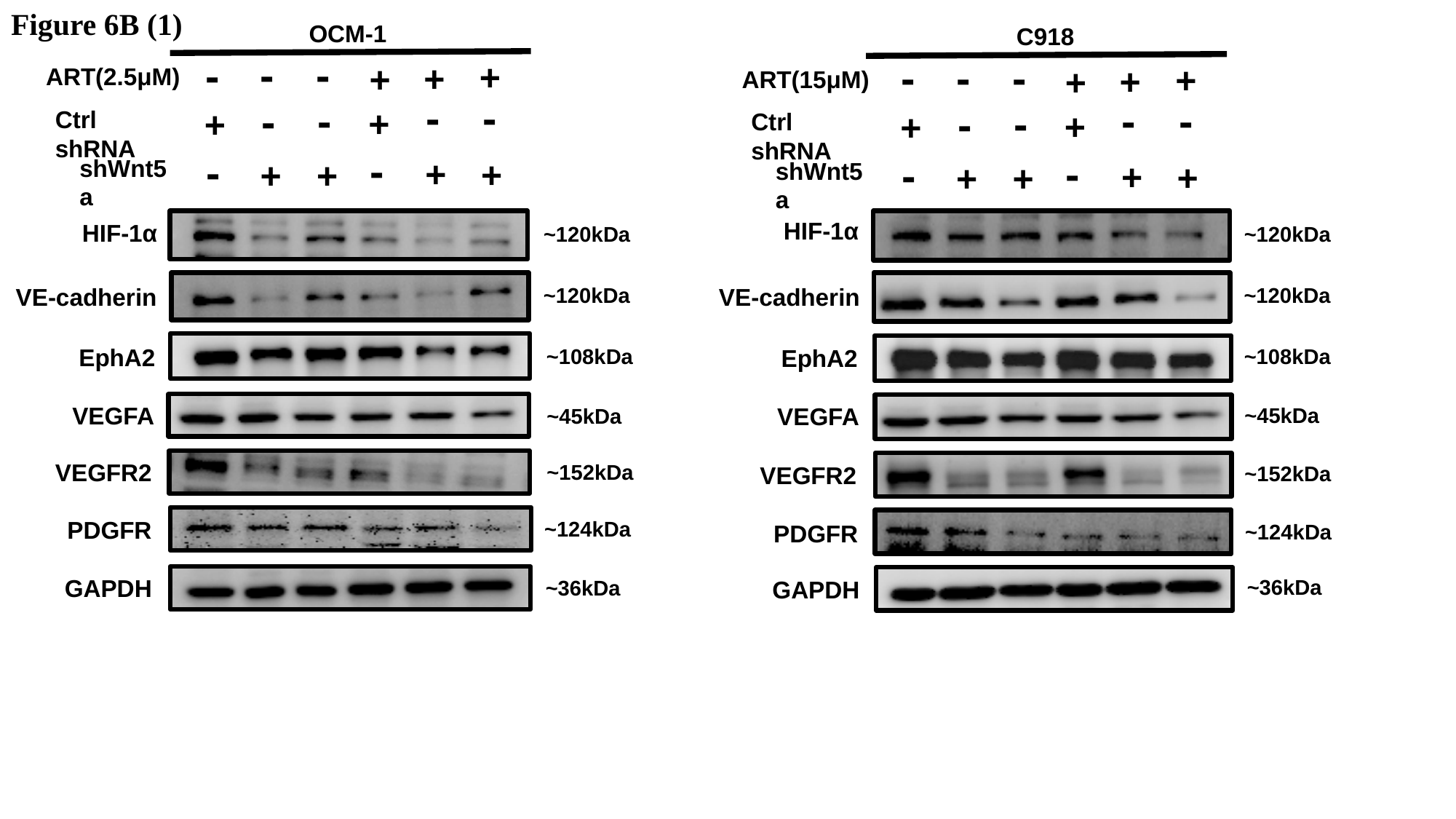

# Figure 6B (1)
OCM-1
-
-
-
+
Ctrl shRNA
+
+
ART(2.5μM)
-
-
-
-
+
+
shWnt5a
-
-
+
+
+
+
C918
-
-
-
+
Ctrl shRNA
+
+
ART(15μM)
-
-
-
-
+
+
shWnt5a
-
-
+
+
+
+
HIF-1α
HIF-1α
~120kDa
~120kDa
VE-cadherin
VE-cadherin
~120kDa
~120kDa
EphA2
EphA2
~108kDa
~108kDa
VEGFA
VEGFA
~45kDa
~45kDa
VEGFR2
~152kDa
VEGFR2
~152kDa
PDGFR
~124kDa
~124kDa
PDGFR
GAPDH
GAPDH
~36kDa
~36kDa

## Slide 30
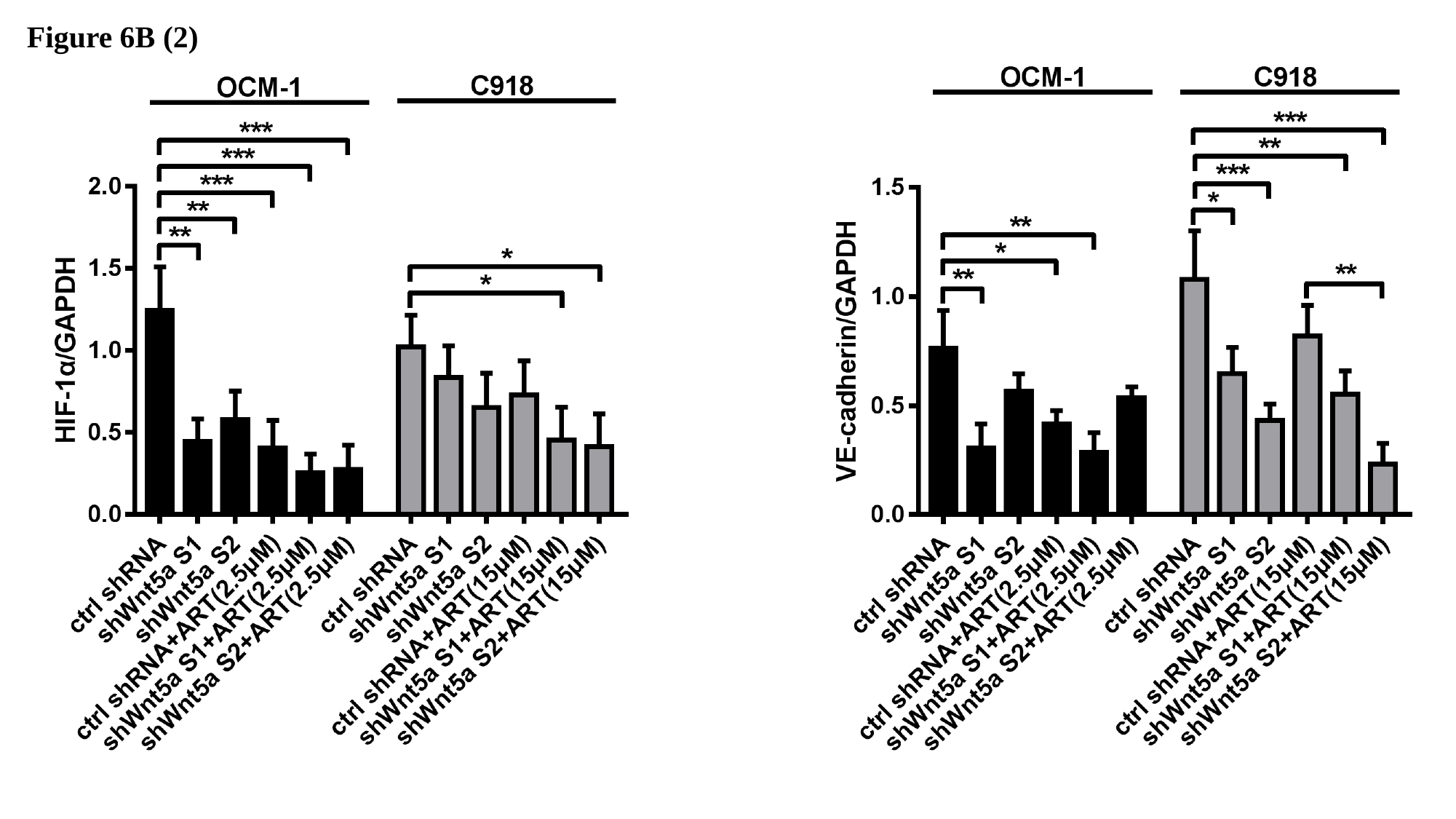

# Figure 6B (2)

## Slide 31
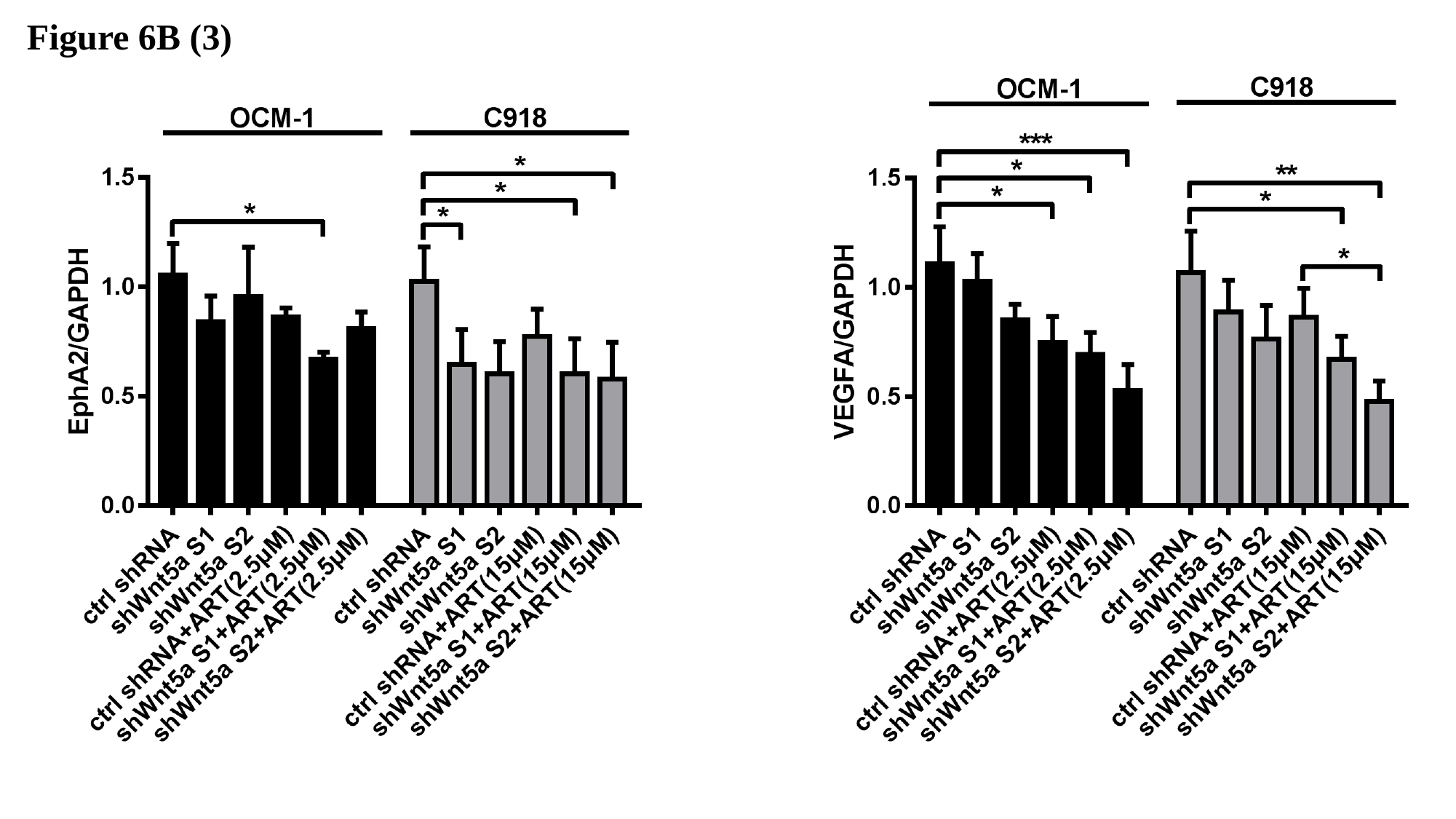

# Figure 6B (3)

## Slide 32
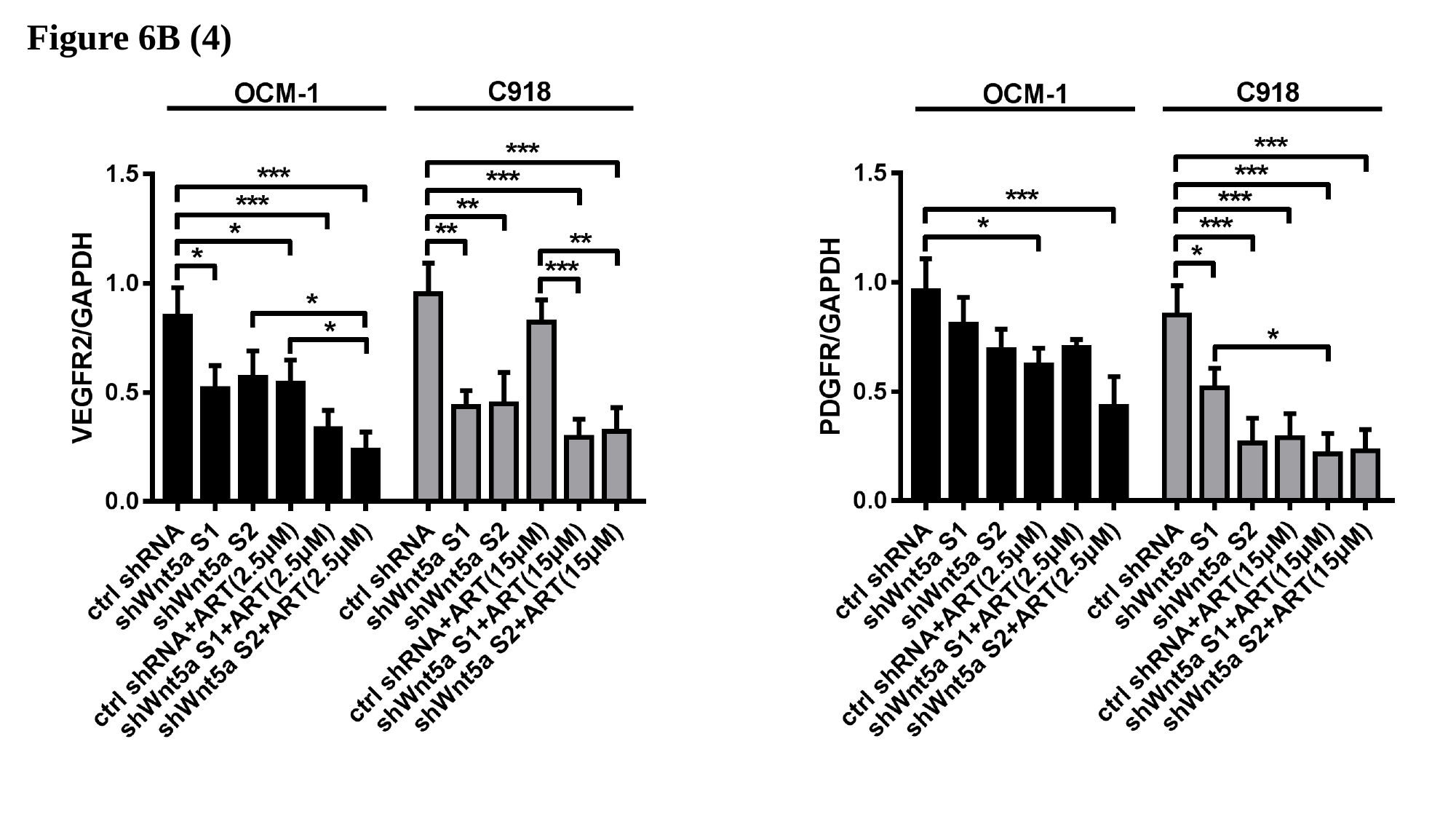

# Figure 6B (4)

## Slide 33
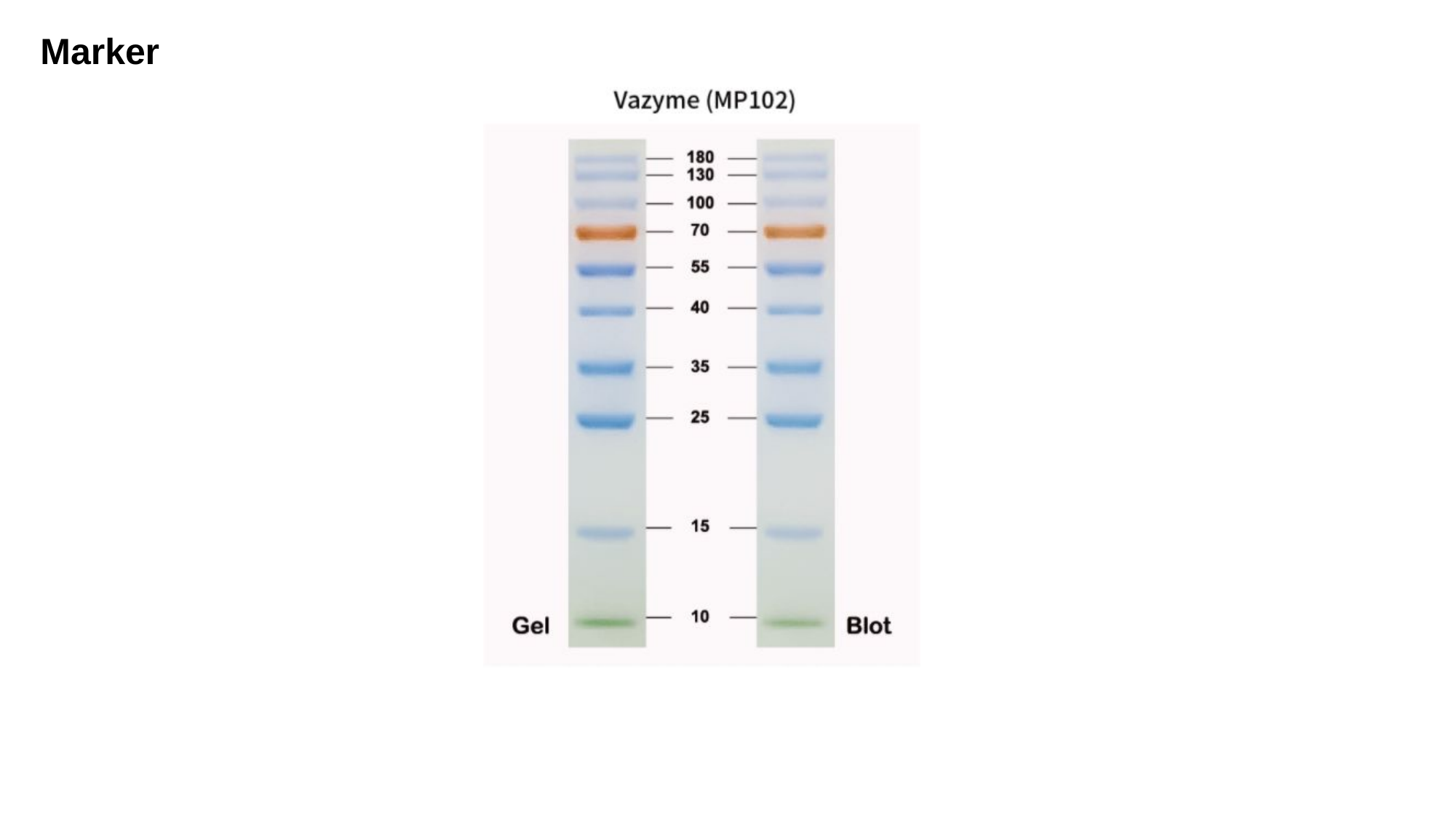

Marker

## Slide 34
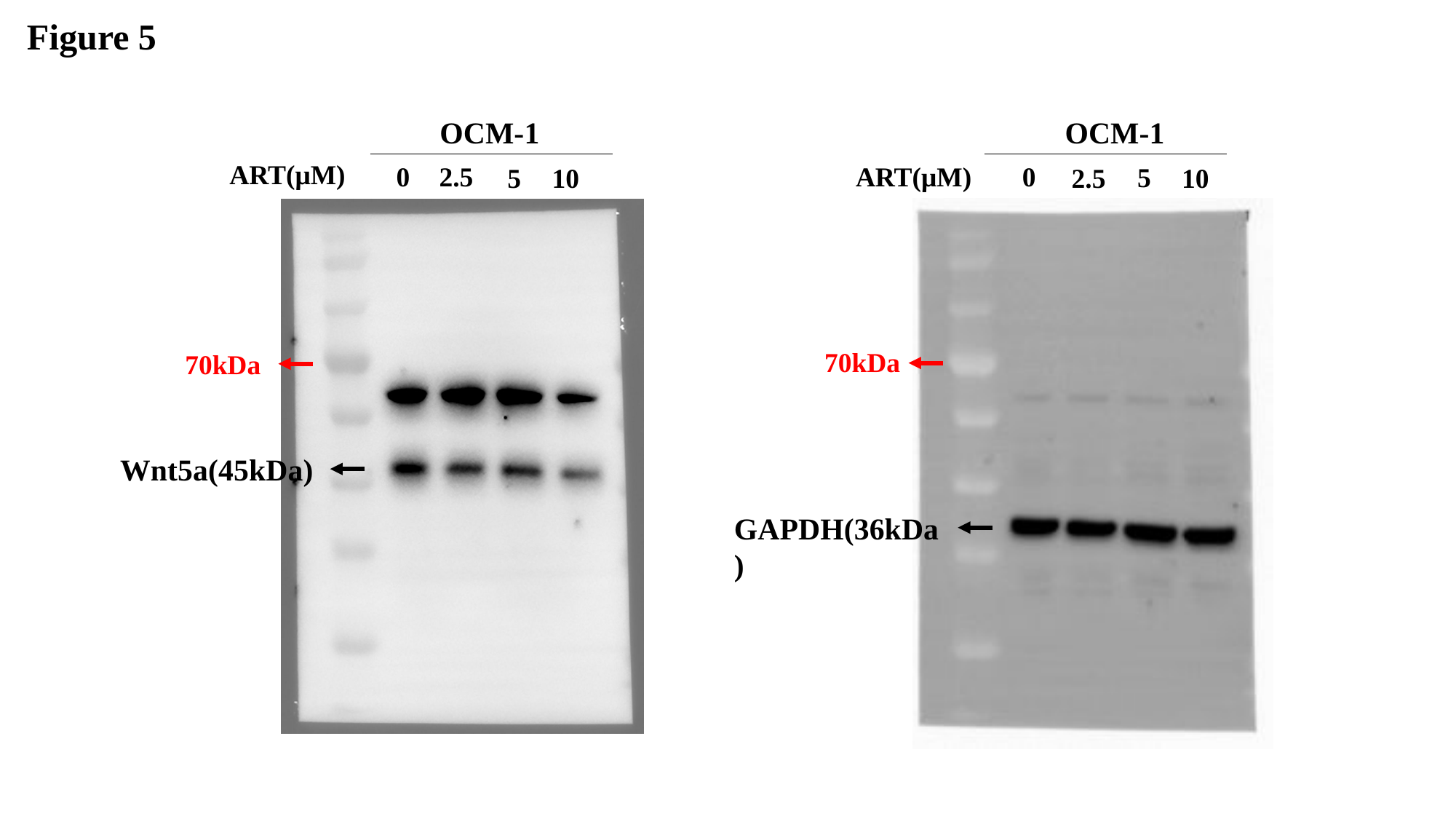

# Figure 5
OCM-1
OCM-1
ART(μM)
0
2.5
ART(μM)
0
5
10
10
5
2.5
70kDa
70kDa
Wnt5a(45kDa)
GAPDH(36kDa)

## Slide 35
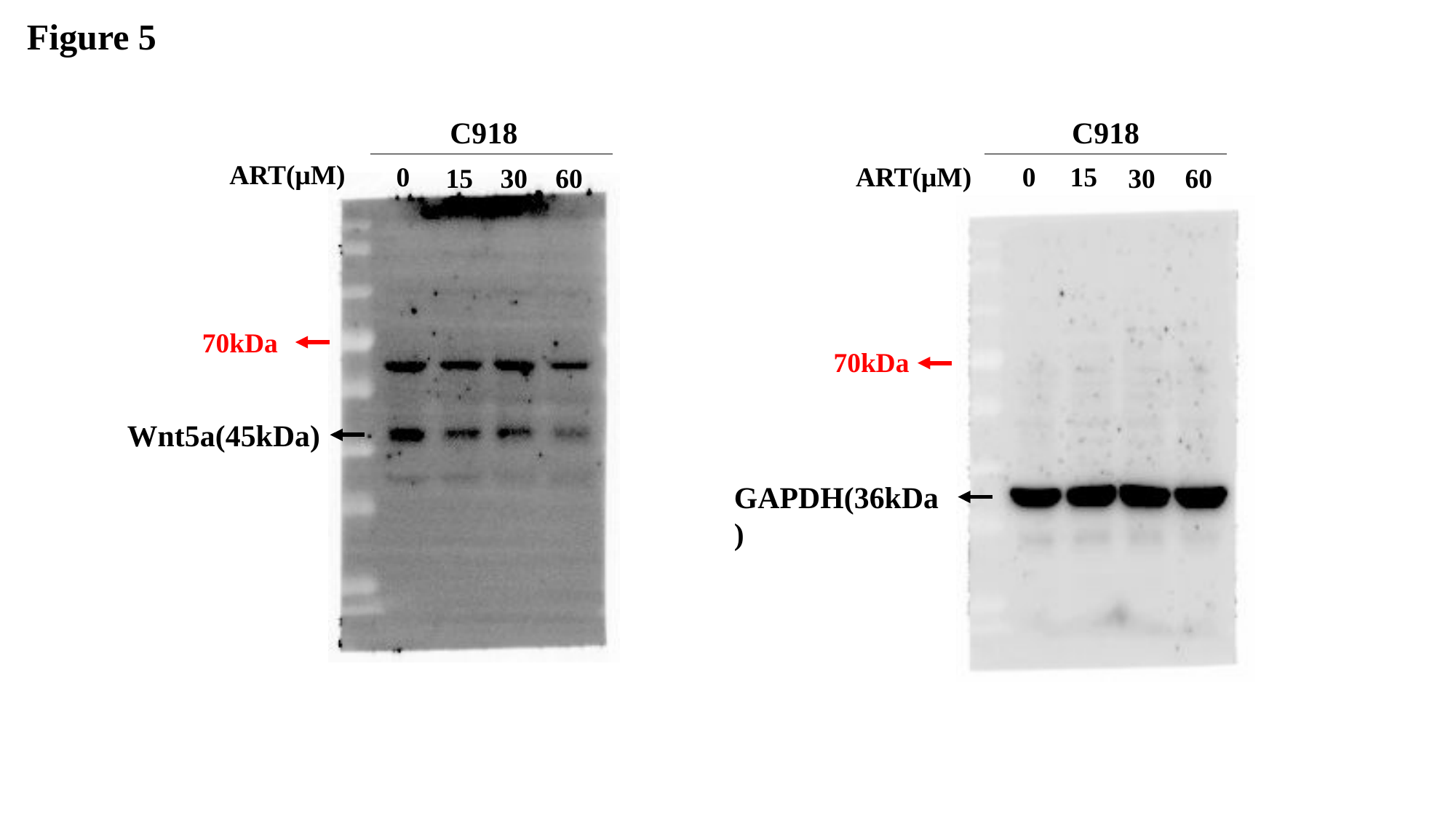

# Figure 5
C918
C918
ART(μM)
0
15
ART(μM)
0
30
30
15
60
60
70kDa
70kDa
Wnt5a(45kDa)
GAPDH(36kDa)

## Slide 36
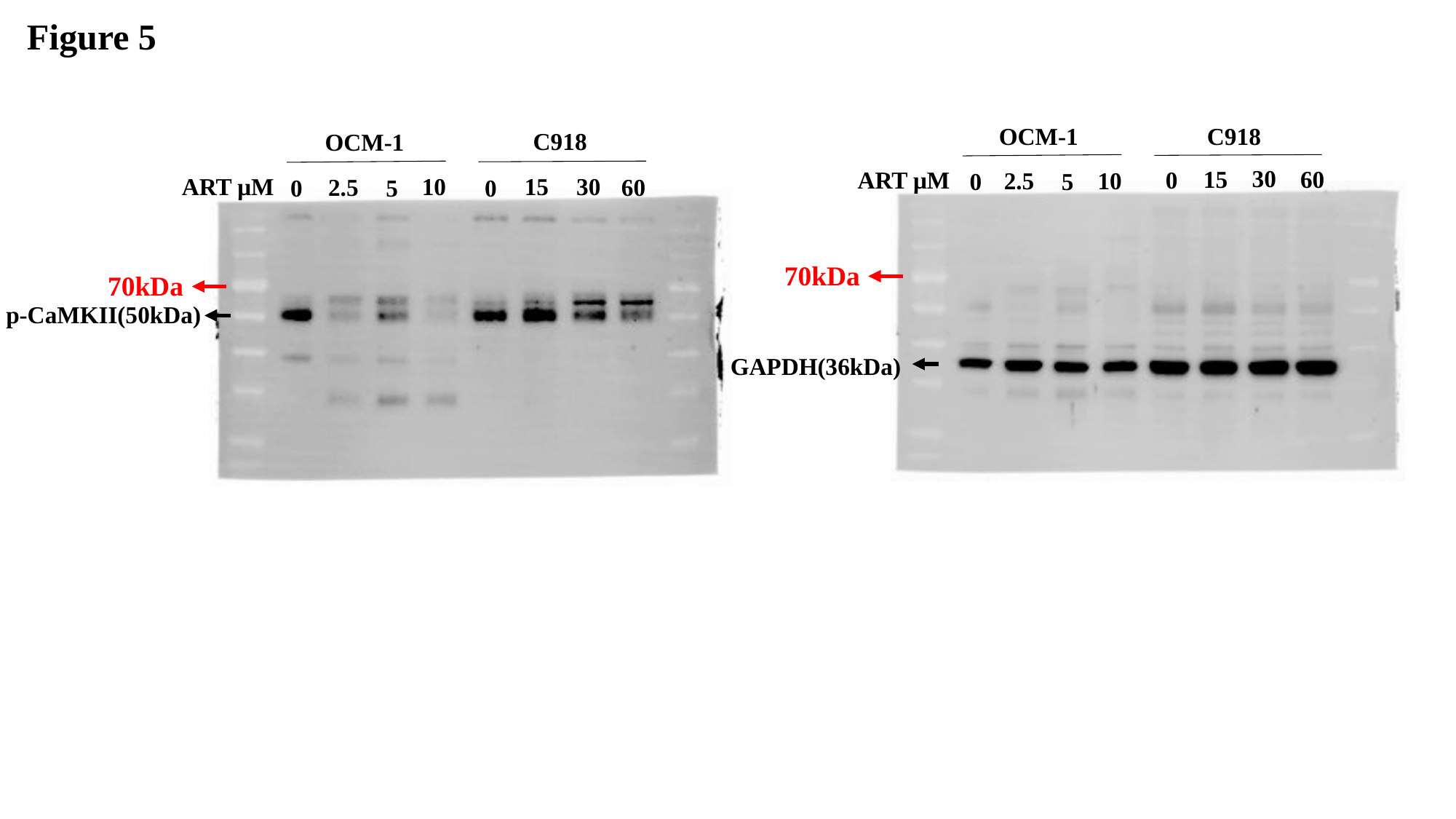

# Figure 5
OCM-1
C918
C918
OCM-1
30
60
15
0
ART μM
10
2.5
0
5
ART μM
15
10
30
60
2.5
0
0
5
70kDa
70kDa
p-CaMKII(50kDa)
GAPDH(36kDa)

## Slide 37
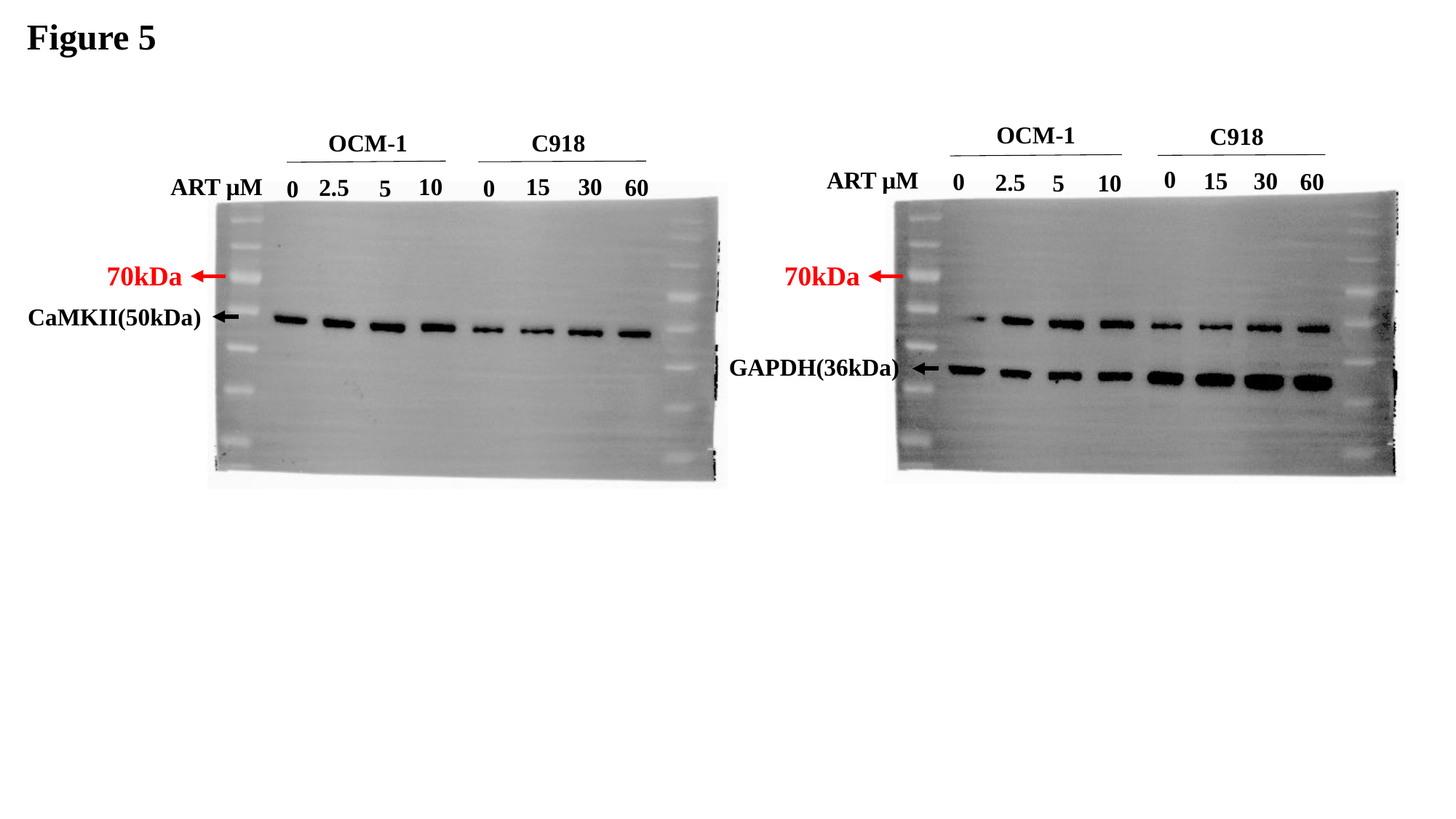

# Figure 5
OCM-1
C918
OCM-1
C918
0
ART μM
30
15
60
0
2.5
10
5
15
ART μM
10
30
60
2.5
5
0
0
70kDa
70kDa
CaMKII(50kDa)
GAPDH(36kDa)

## Slide 38
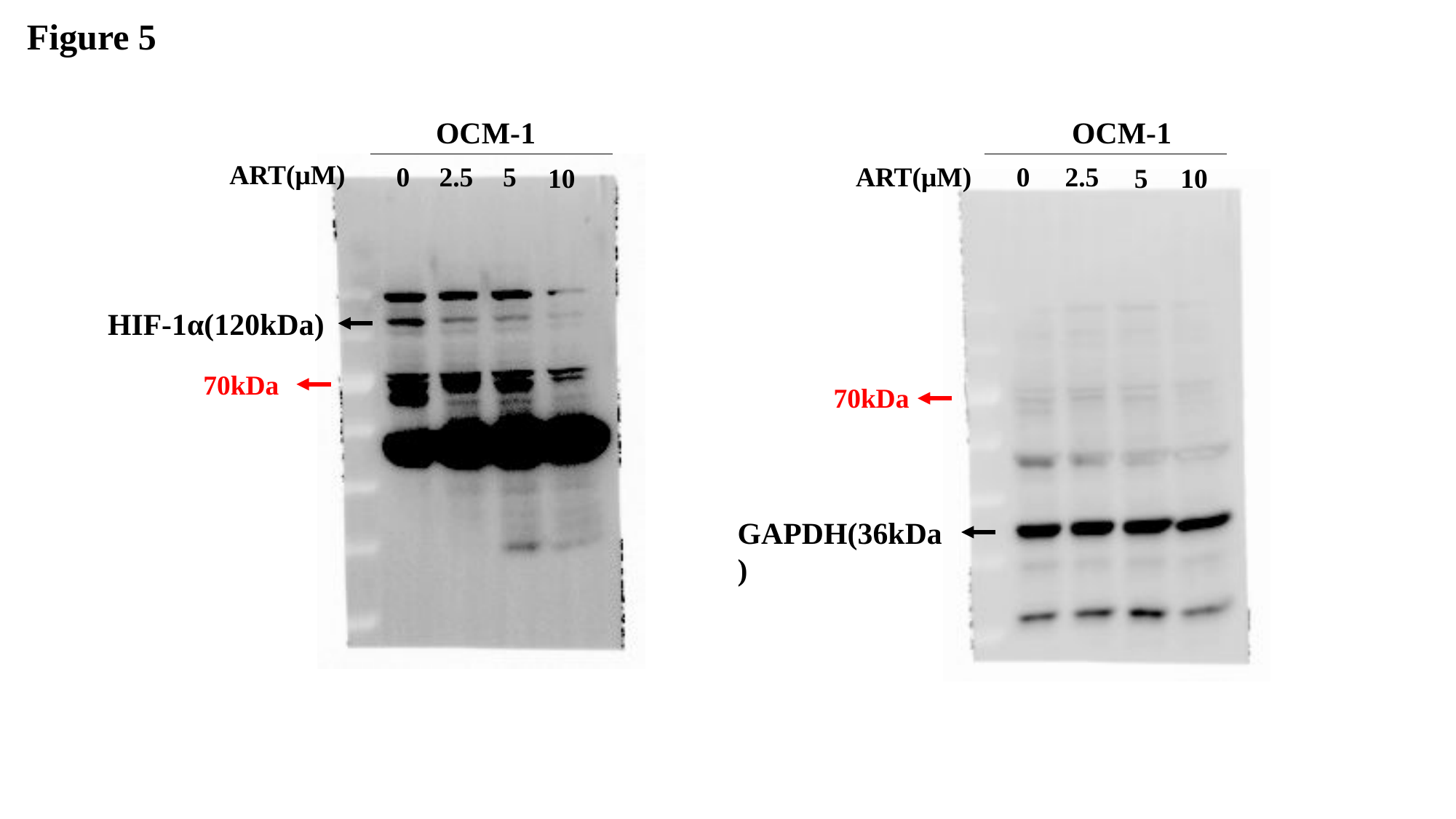

# Figure 5
OCM-1
OCM-1
ART(μM)
5
0
2.5
2.5
ART(μM)
0
5
10
10
HIF-1α(120kDa)
70kDa
70kDa
GAPDH(36kDa)

## Slide 39
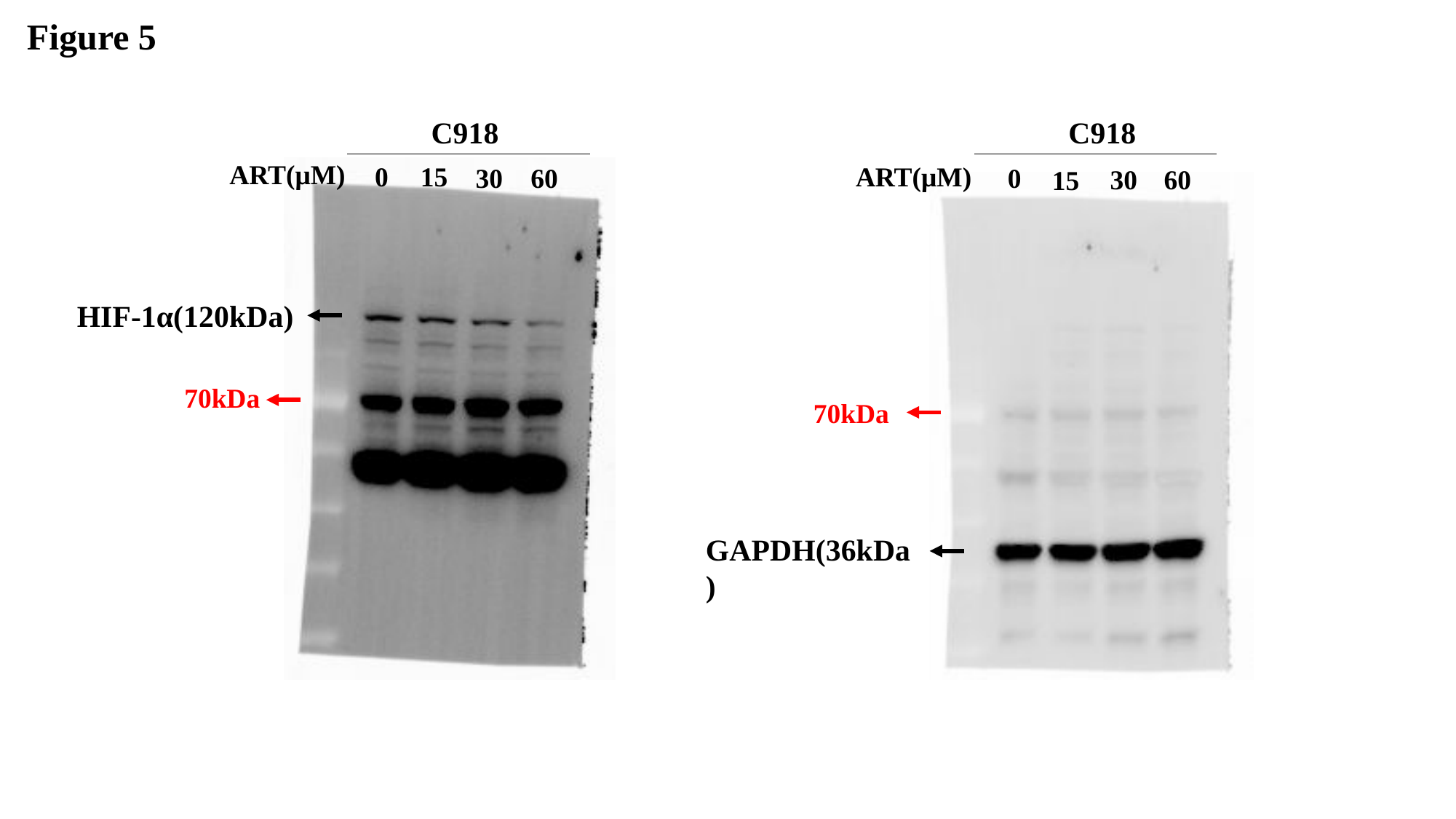

# Figure 5
C918
C918
ART(μM)
15
ART(μM)
0
60
0
30
30
60
15
HIF-1α(120kDa)
70kDa
70kDa
GAPDH(36kDa)

## Slide 40
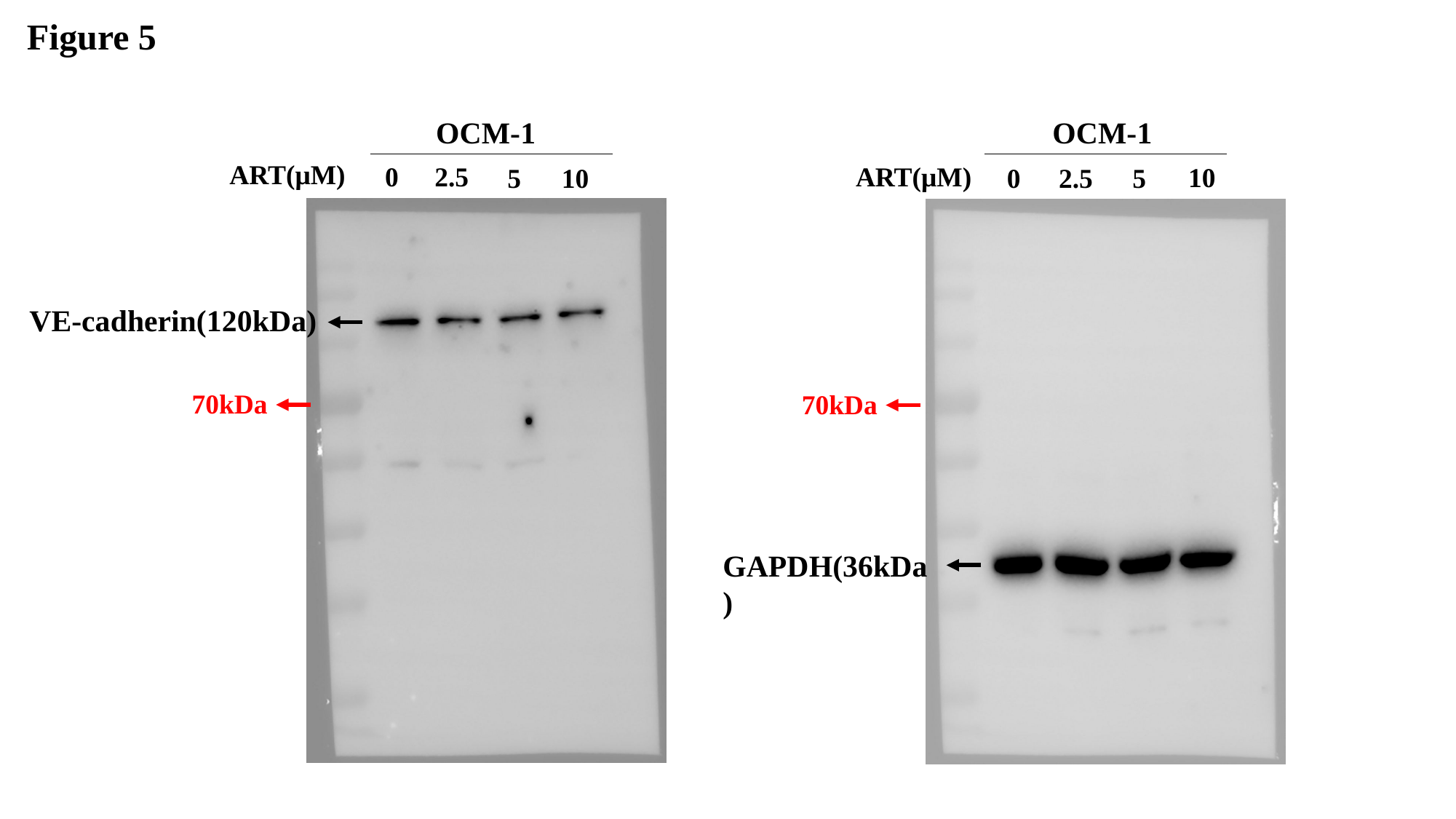

# Figure 5
OCM-1
OCM-1
ART(μM)
2.5
ART(μM)
0
10
2.5
10
5
5
0
VE-cadherin(120kDa)
70kDa
70kDa
GAPDH(36kDa)

## Slide 41
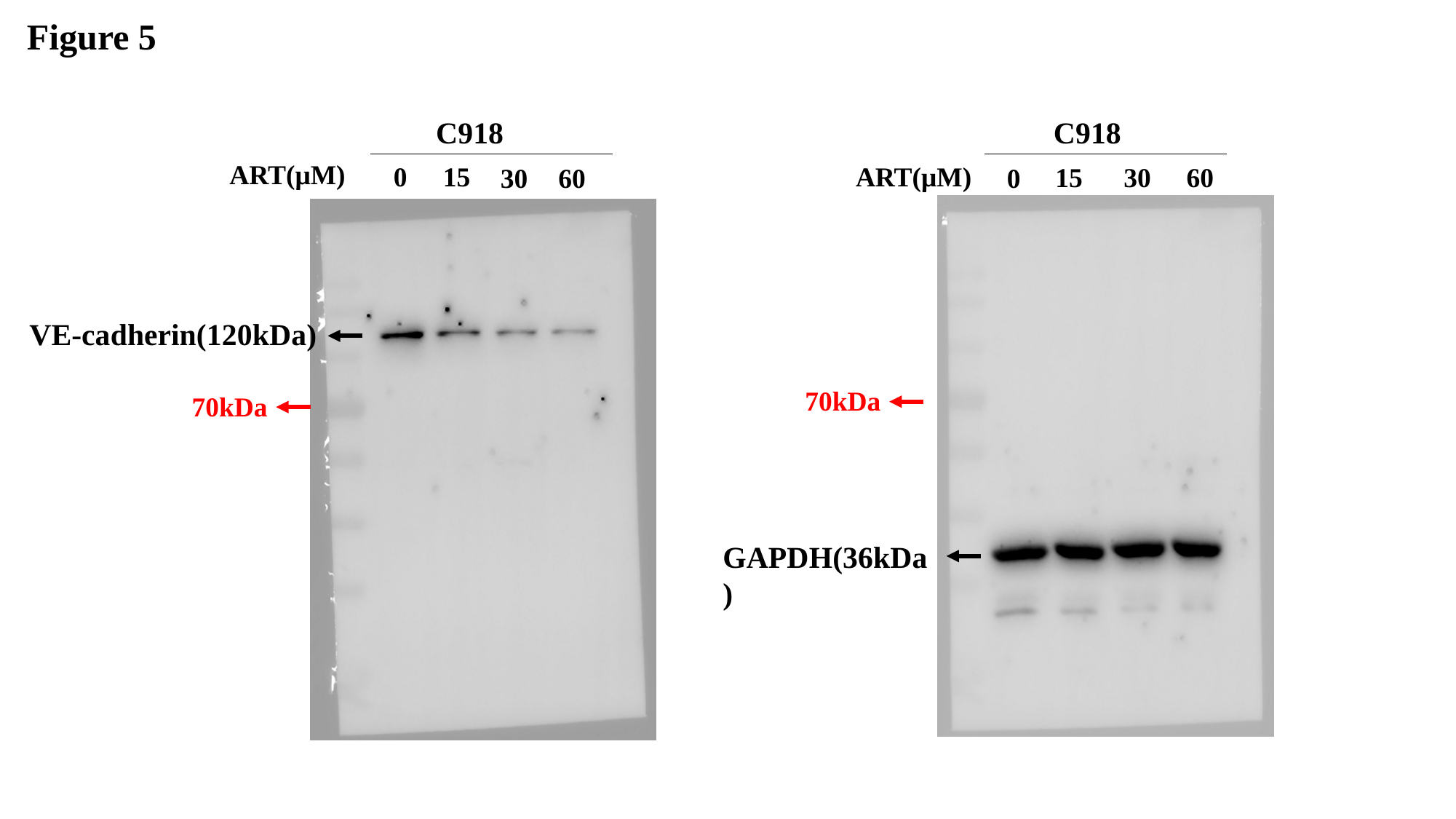

# Figure 5
C918
C918
ART(μM)
15
ART(μM)
0
15
60
30
60
30
0
VE-cadherin(120kDa)
70kDa
70kDa
GAPDH(36kDa)

## Slide 42
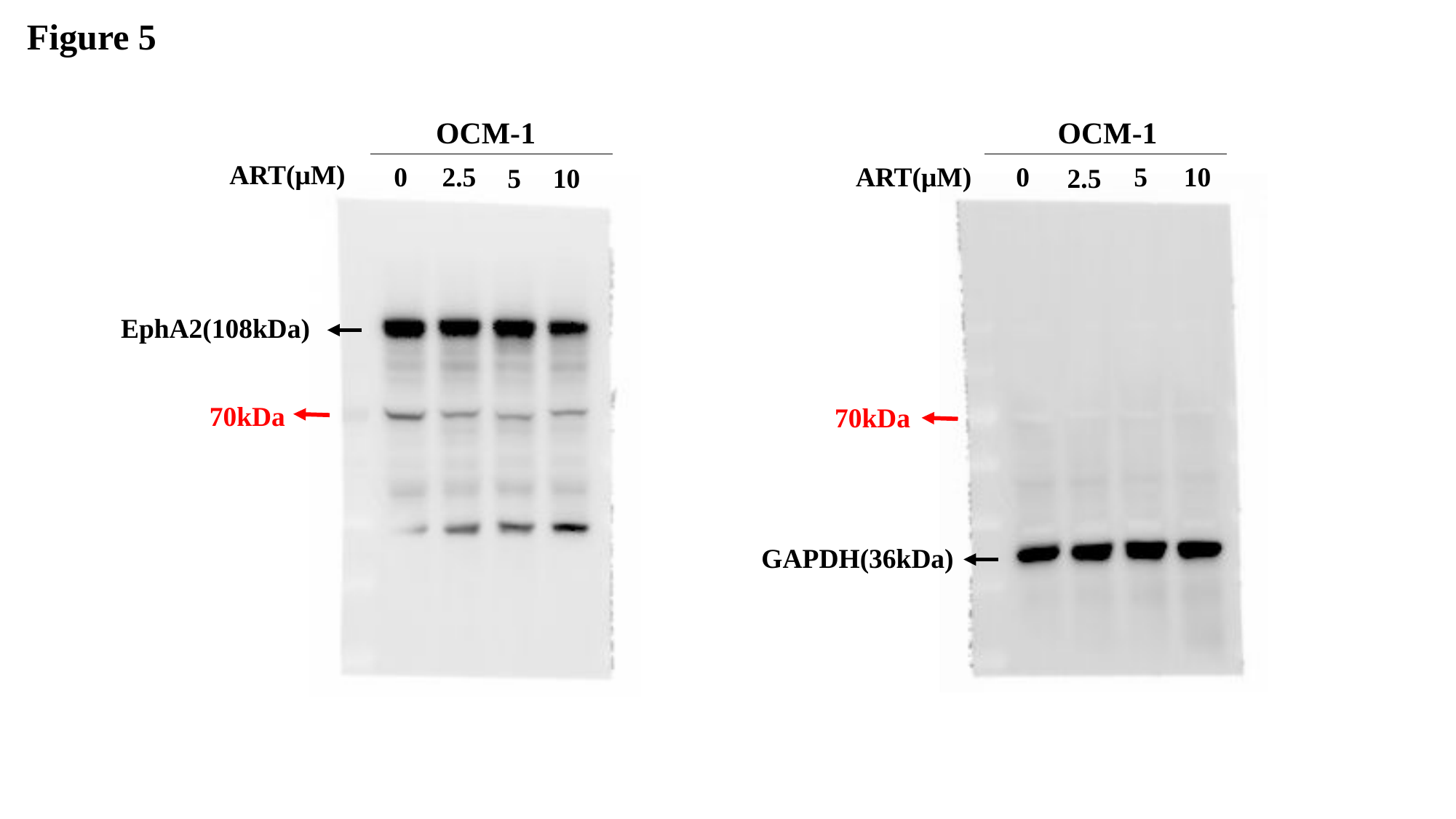

# Figure 5
OCM-1
OCM-1
ART(μM)
0
2.5
ART(μM)
0
5
10
2.5
10
5
EphA2(108kDa)
70kDa
70kDa
GAPDH(36kDa)

## Slide 43
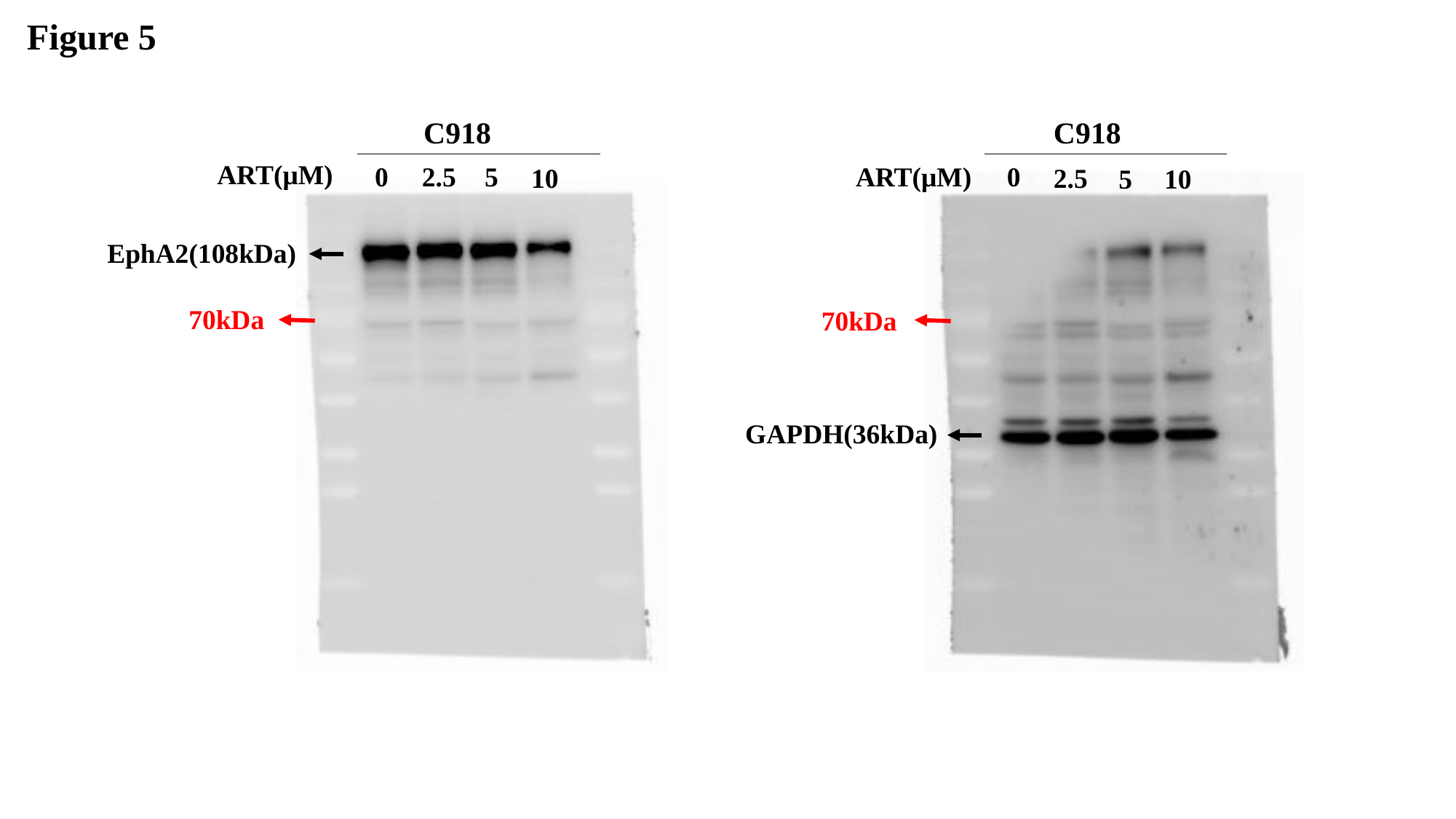

# Figure 5
C918
C918
ART(μM)
5
0
2.5
ART(μM)
0
2.5
10
5
10
EphA2(108kDa)
70kDa
70kDa
GAPDH(36kDa)

## Slide 44
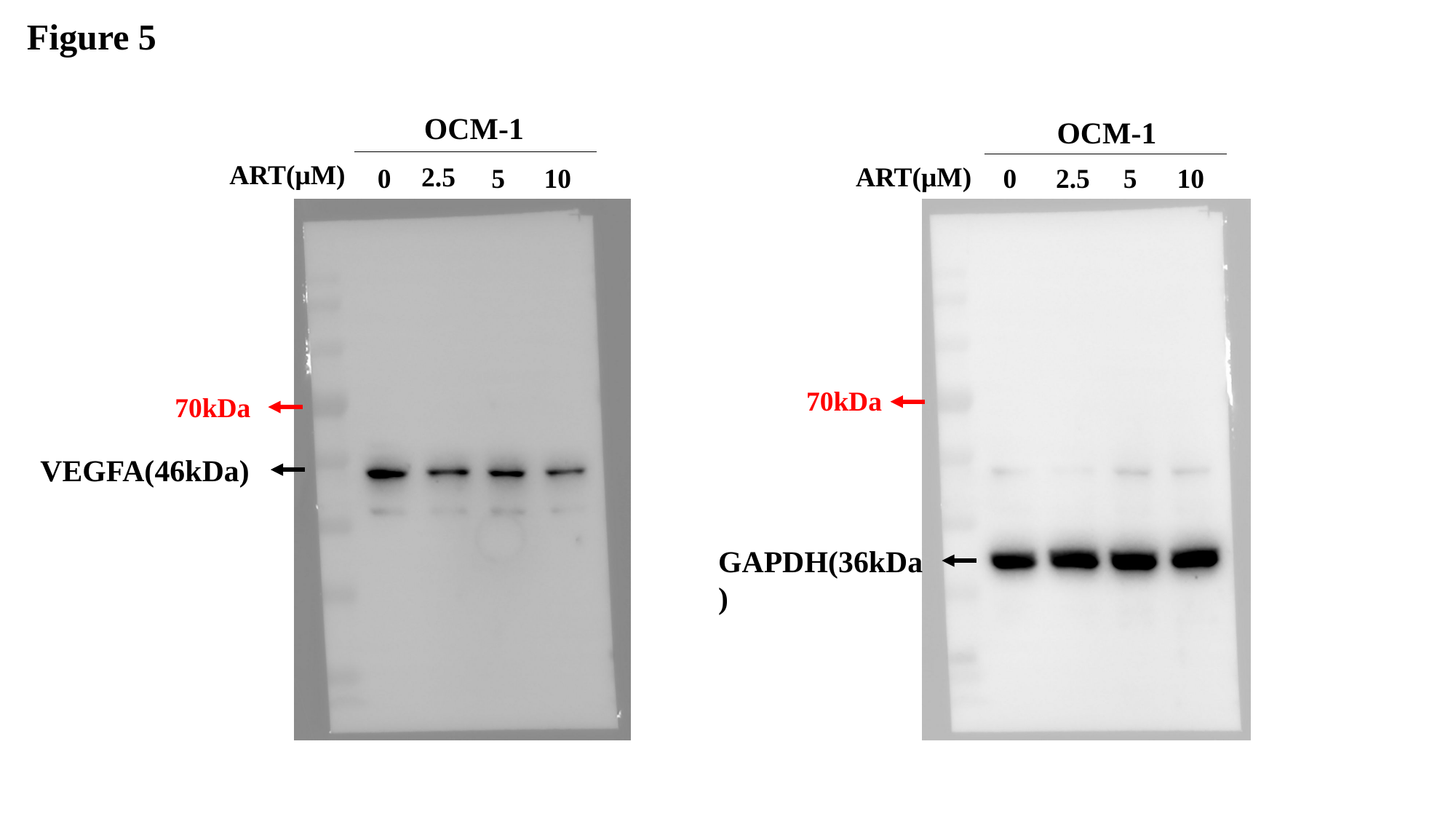

# Figure 5
OCM-1
OCM-1
ART(μM)
2.5
ART(μM)
10
10
0
0
5
2.5
5
70kDa
70kDa
VEGFA(46kDa)
GAPDH(36kDa)

## Slide 45
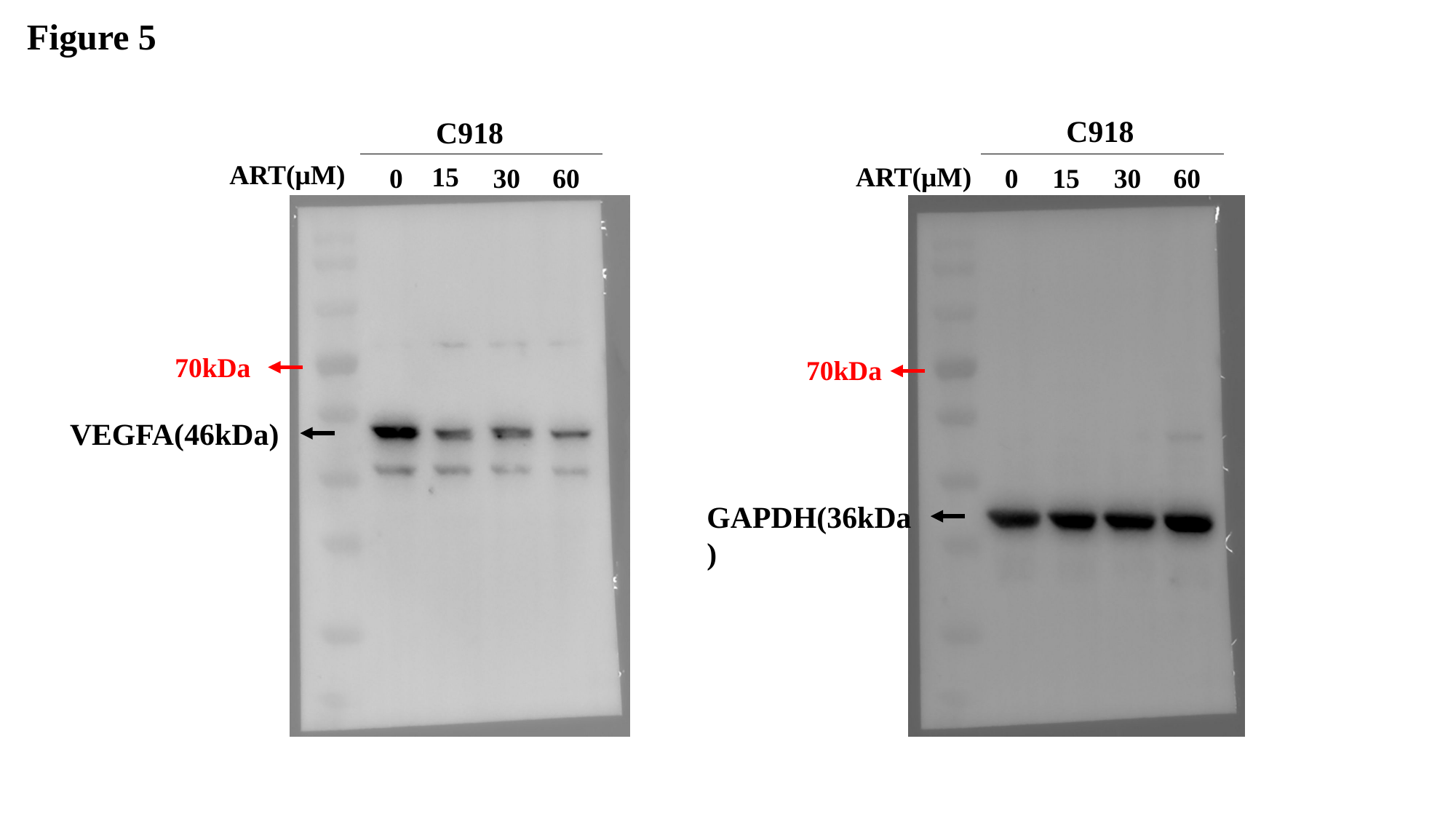

# Figure 5
C918
C918
ART(μM)
15
ART(μM)
60
60
0
0
30
15
30
70kDa
70kDa
VEGFA(46kDa)
GAPDH(36kDa)

## Slide 46
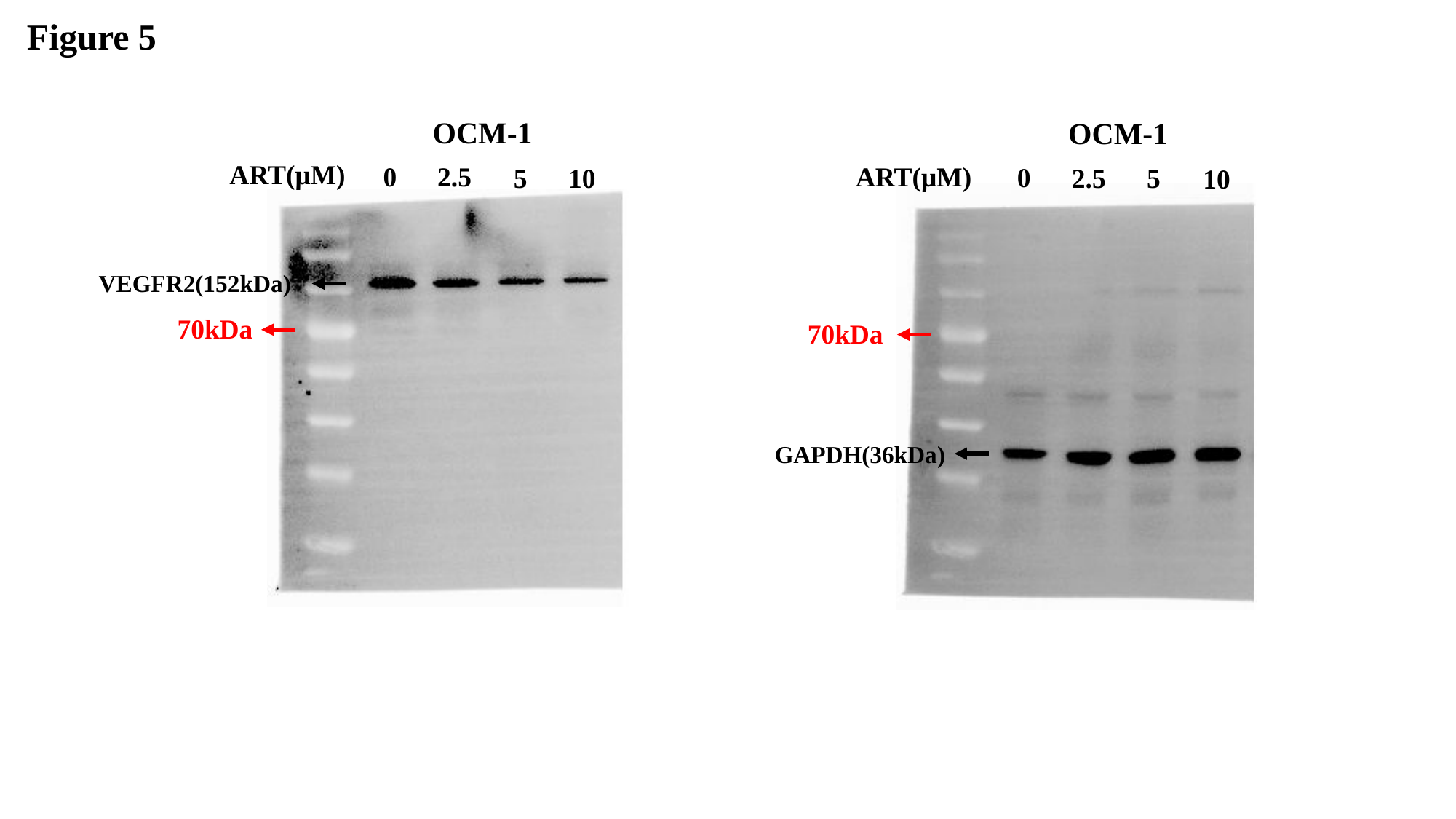

# Figure 5
OCM-1
OCM-1
ART(μM)
2.5
ART(μM)
0
0
5
2.5
10
5
10
VEGFR2(152kDa)
70kDa
70kDa
GAPDH(36kDa)

## Slide 47
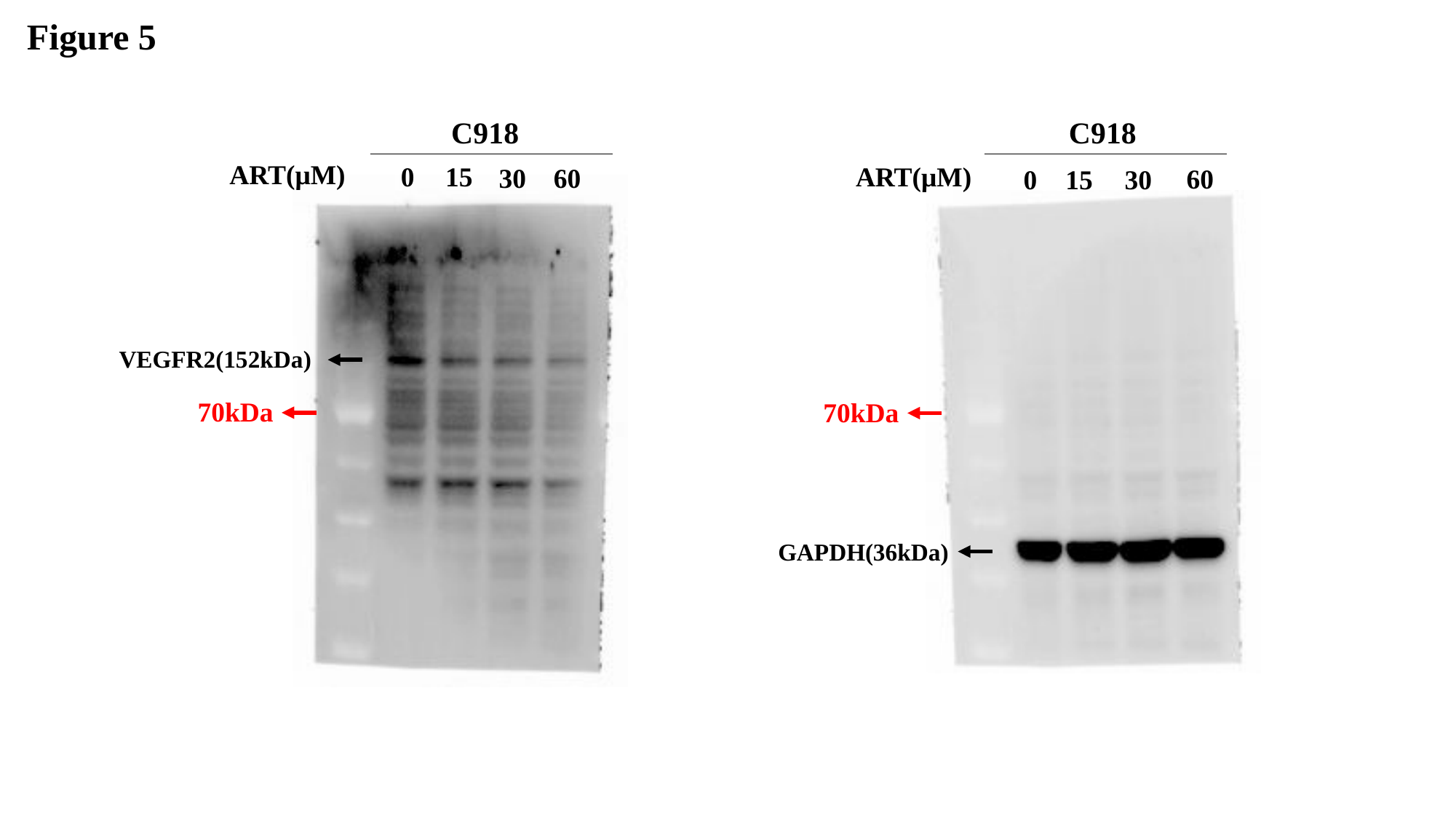

# Figure 5
C918
C918
ART(μM)
15
ART(μM)
0
60
30
60
0
30
15
VEGFR2(152kDa)
70kDa
70kDa
GAPDH(36kDa)

## Slide 48
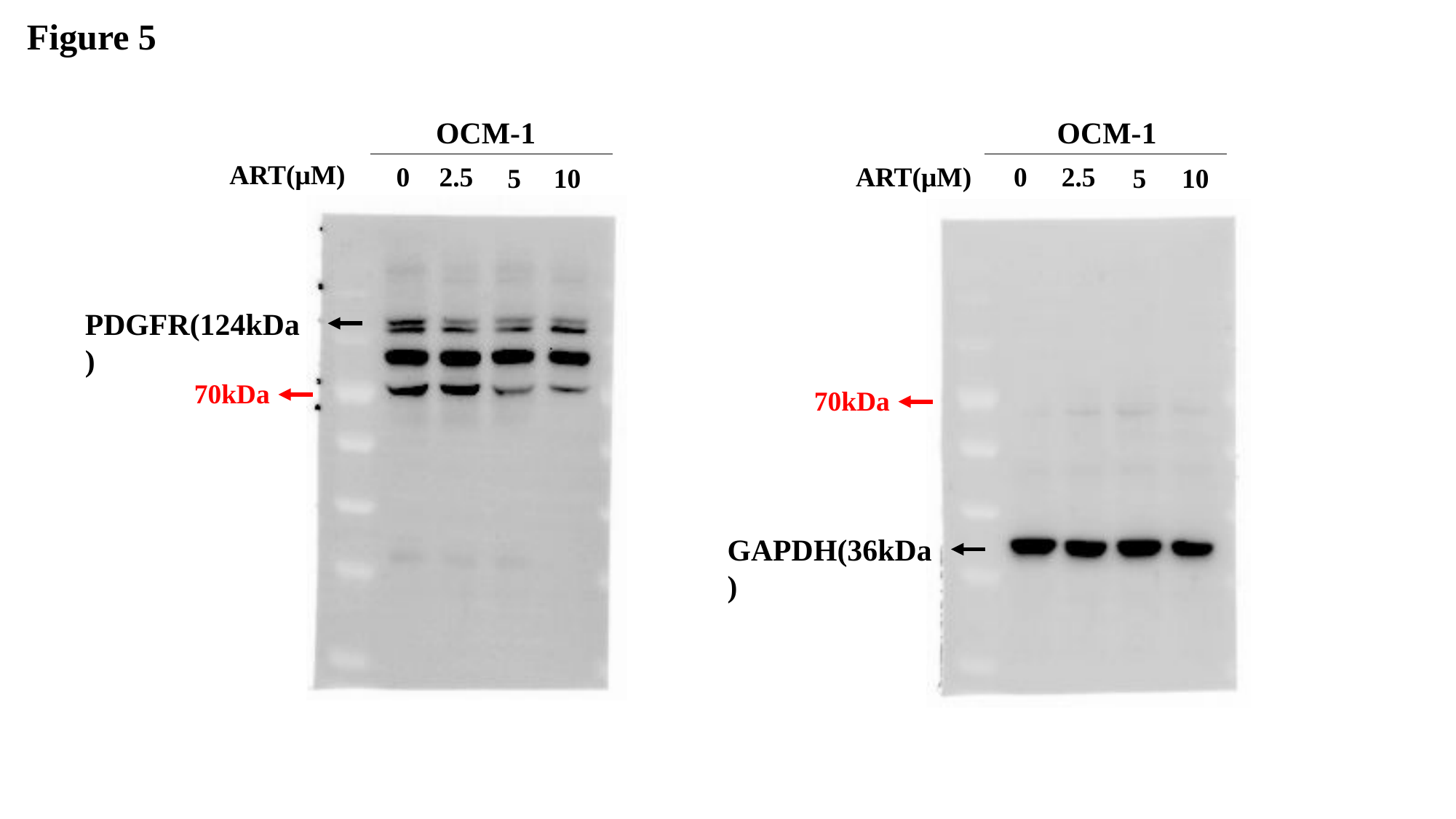

# Figure 5
OCM-1
OCM-1
ART(μM)
0
2.5
2.5
ART(μM)
0
5
10
10
5
PDGFR(124kDa)
70kDa
70kDa
GAPDH(36kDa)

## Slide 49
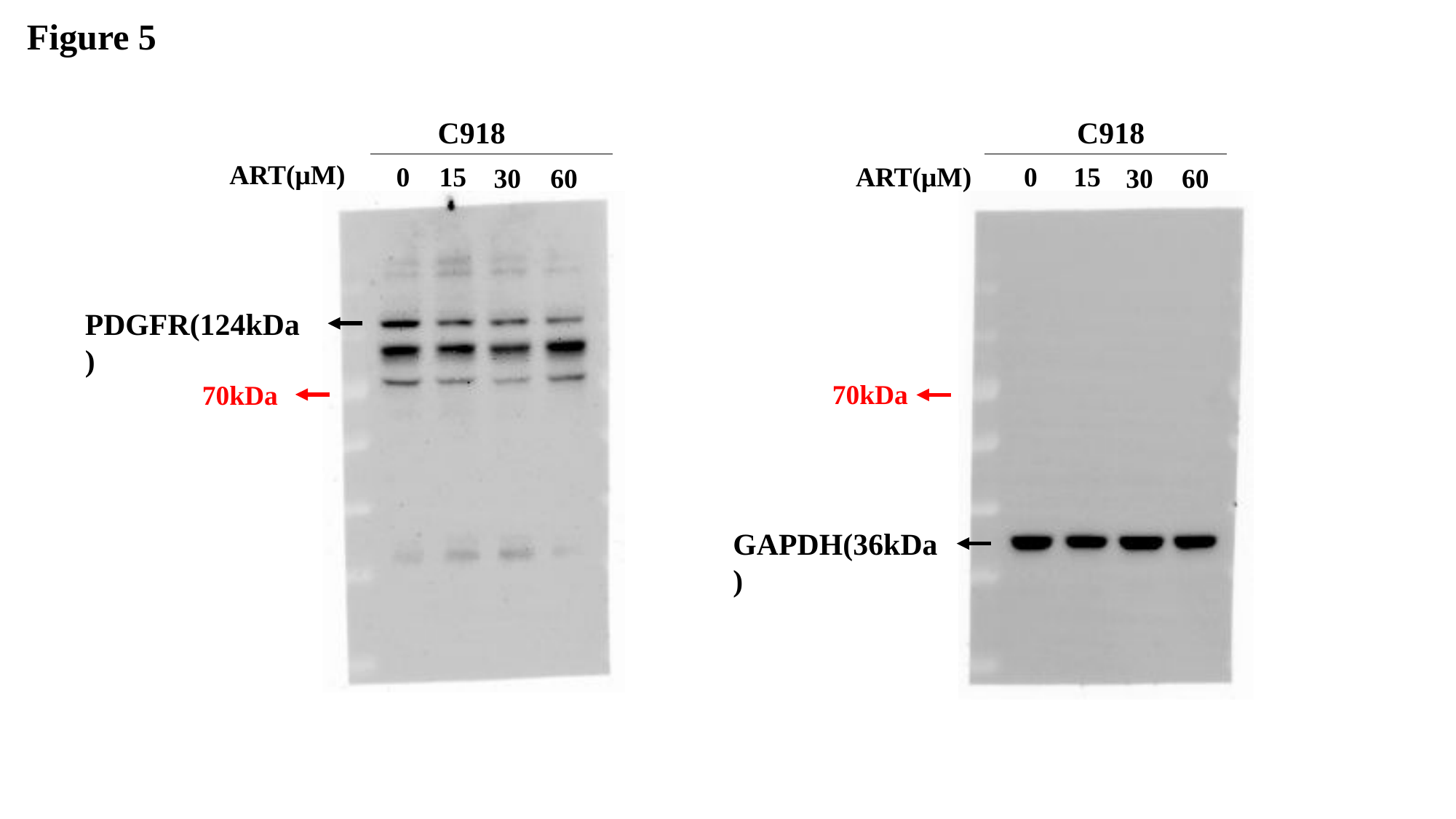

# Figure 5
C918
C918
ART(μM)
0
15
15
ART(μM)
0
30
30
60
60
PDGFR(124kDa)
70kDa
70kDa
GAPDH(36kDa)

## Slide 50
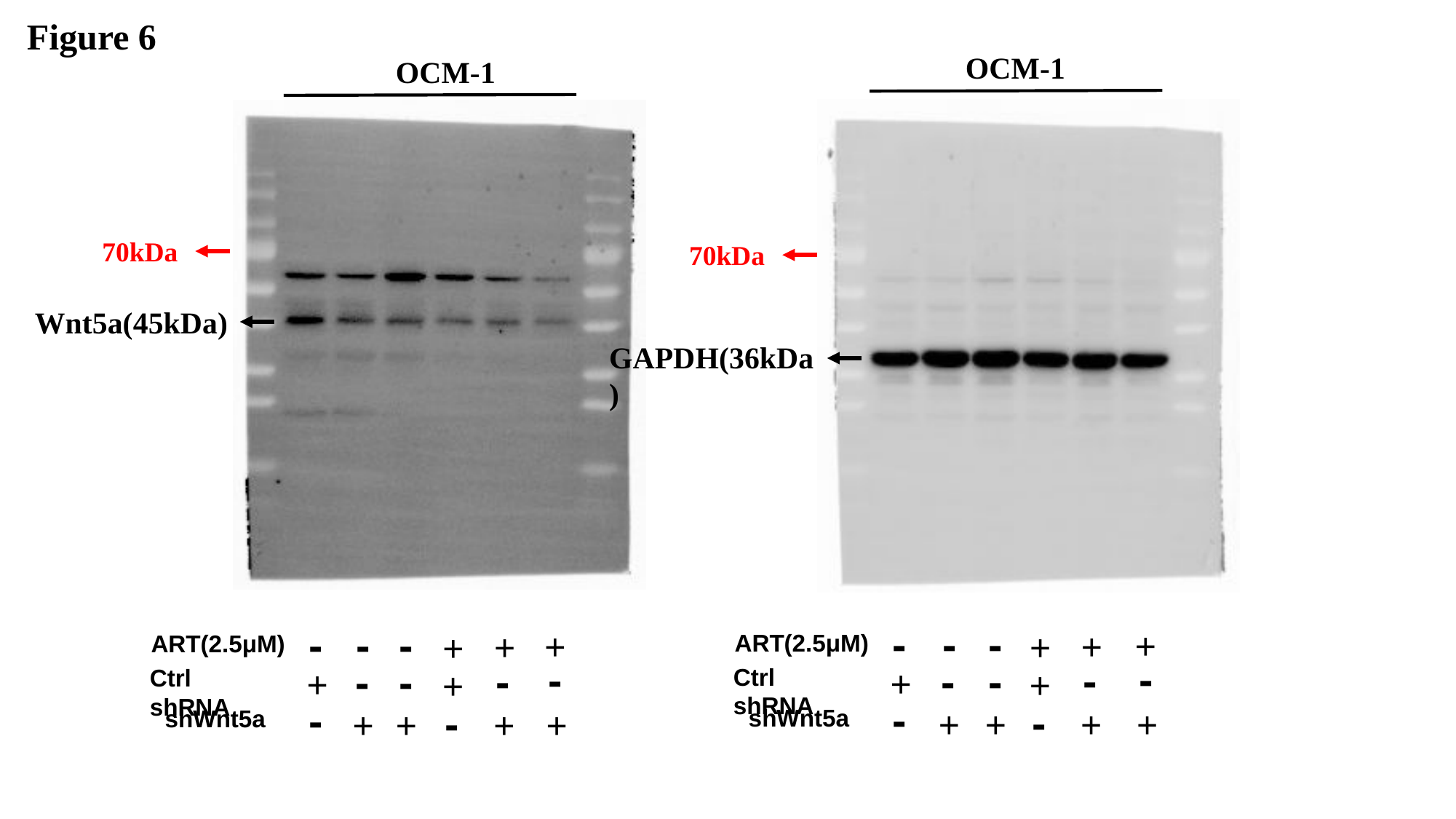

# Figure 6
OCM-1
OCM-1
70kDa
70kDa
Wnt5a(45kDa)
GAPDH(36kDa)
Ctrl shRNA
Ctrl shRNA
-
-
-
-
-
-
+
+
+
+
+
+
ART(2.5μM)
ART(2.5μM)
-
-
-
-
-
-
-
-
+
shWnt5a
+
shWnt5a
+
+
-
-
-
-
+
+
+
+
+
+
+
+

## Slide 51
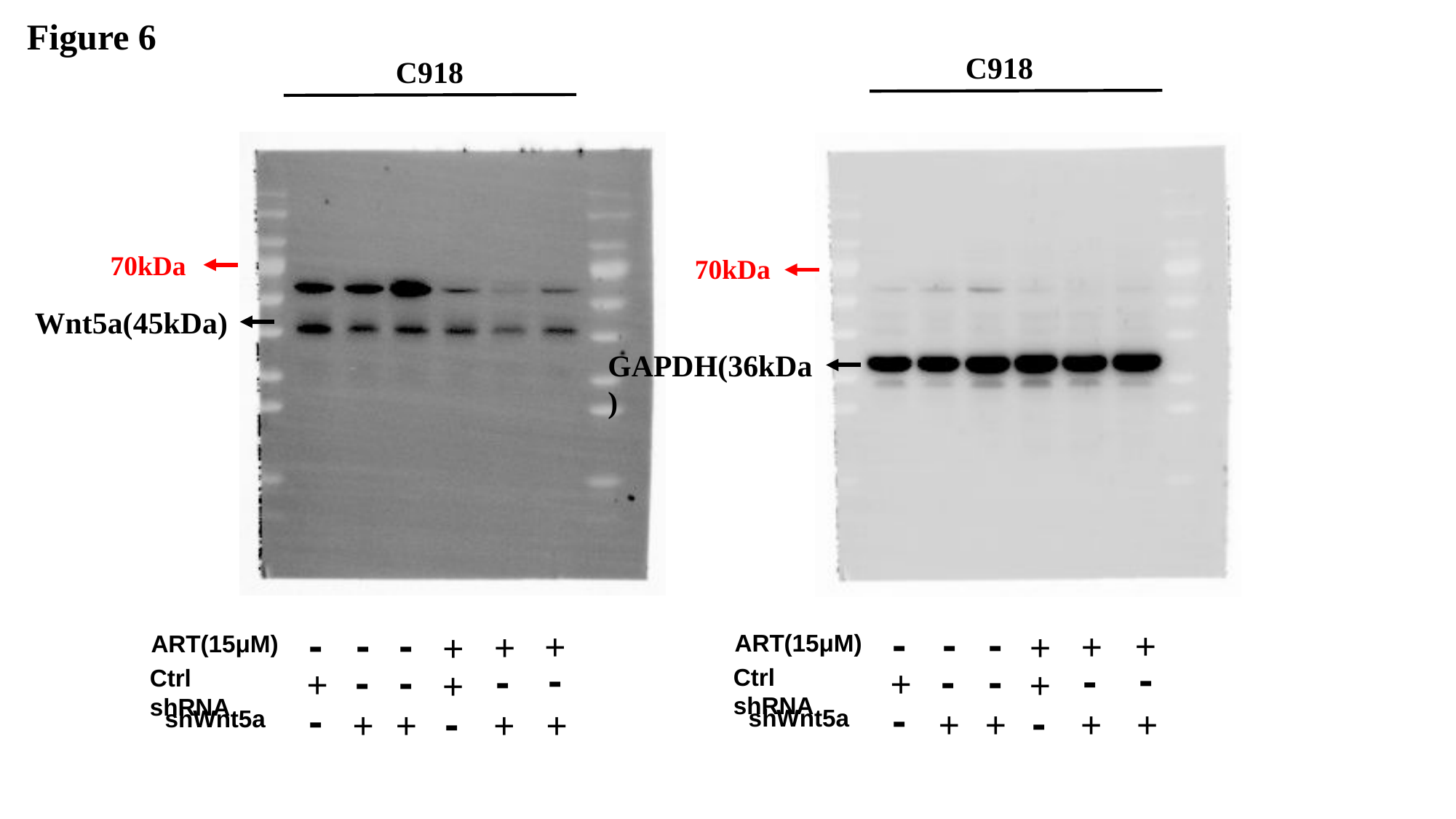

# Figure 6
C918
C918
70kDa
70kDa
Wnt5a(45kDa)
GAPDH(36kDa)
Ctrl shRNA
Ctrl shRNA
-
-
-
-
-
-
+
+
+
+
+
+
ART(15μM)
ART(15μM)
-
-
-
-
-
-
-
-
+
shWnt5a
+
shWnt5a
+
+
-
-
-
-
+
+
+
+
+
+
+
+

## Slide 52
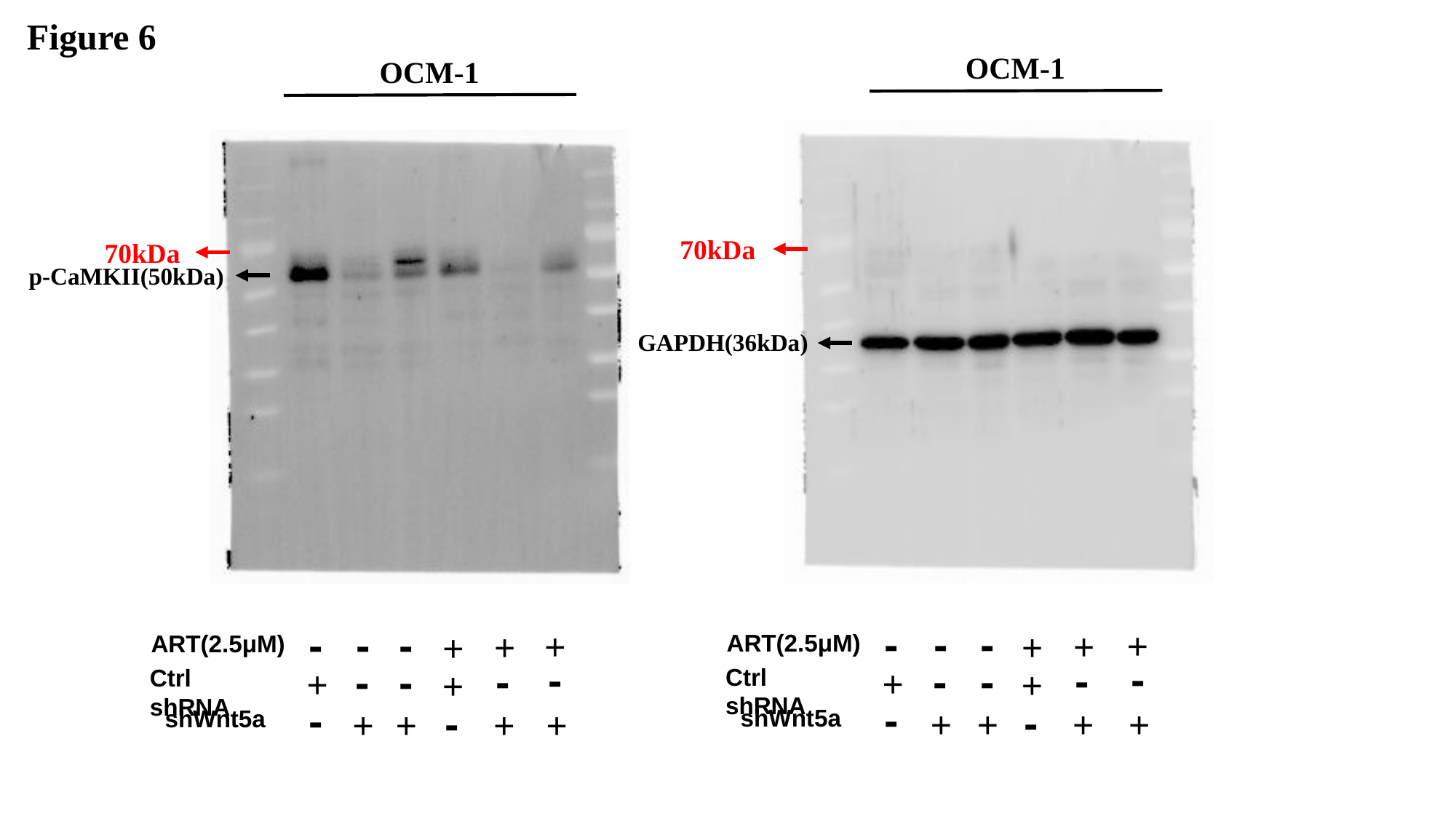

# Figure 6
OCM-1
OCM-1
70kDa
70kDa
p-CaMKII(50kDa)
GAPDH(36kDa)
Ctrl shRNA
Ctrl shRNA
-
-
-
-
-
-
+
+
+
+
+
+
ART(2.5μM)
ART(2.5μM)
-
-
-
-
-
-
-
-
+
shWnt5a
+
shWnt5a
+
+
-
-
-
-
+
+
+
+
+
+
+
+

## Slide 53
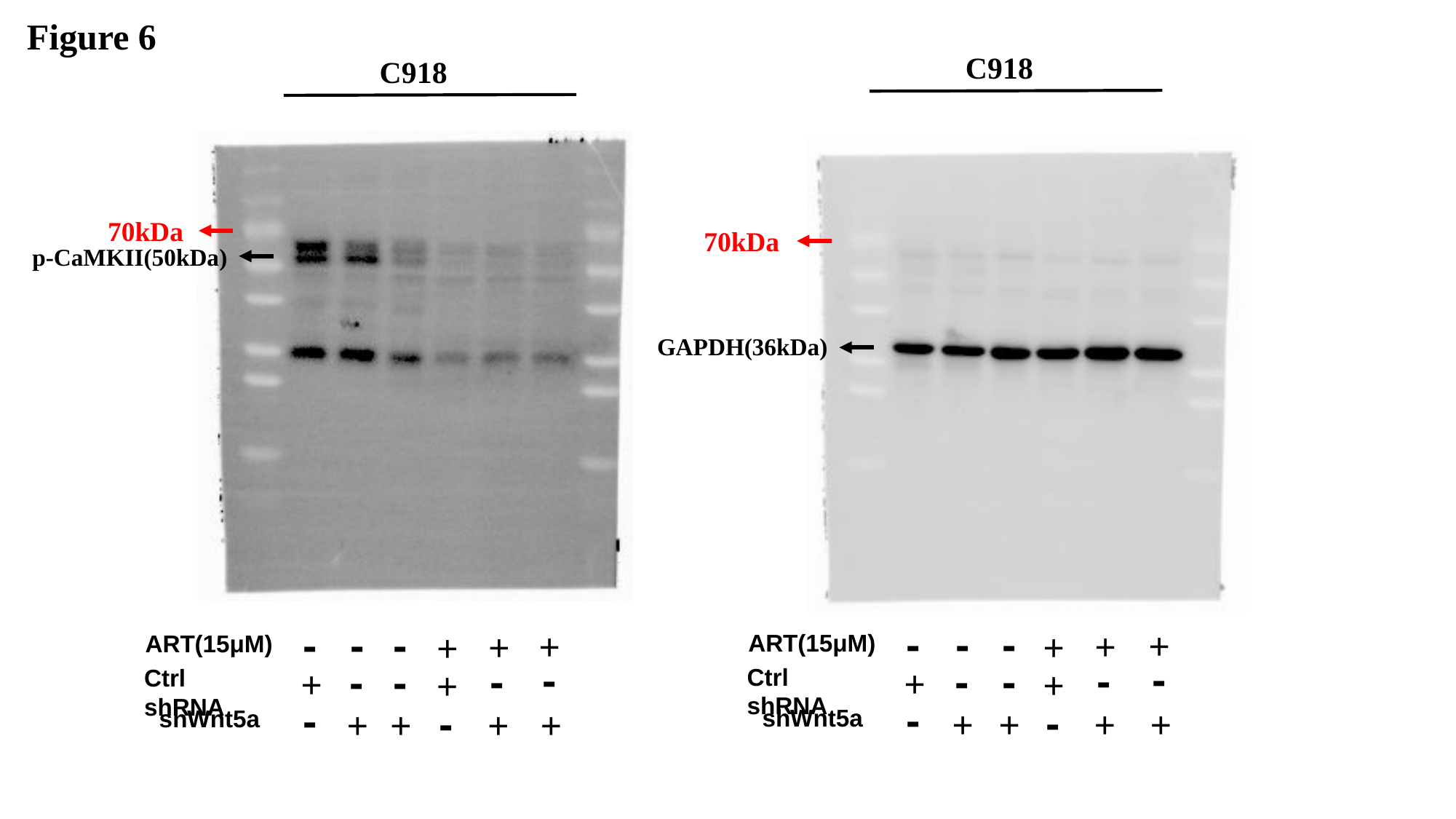

# Figure 6
C918
C918
70kDa
70kDa
p-CaMKII(50kDa)
GAPDH(36kDa)
Ctrl shRNA
Ctrl shRNA
-
-
-
-
-
-
+
+
+
+
+
+
ART(15μM)
ART(15μM)
-
-
-
-
-
-
-
-
+
shWnt5a
+
shWnt5a
+
+
-
-
-
-
+
+
+
+
+
+
+
+

## Slide 54
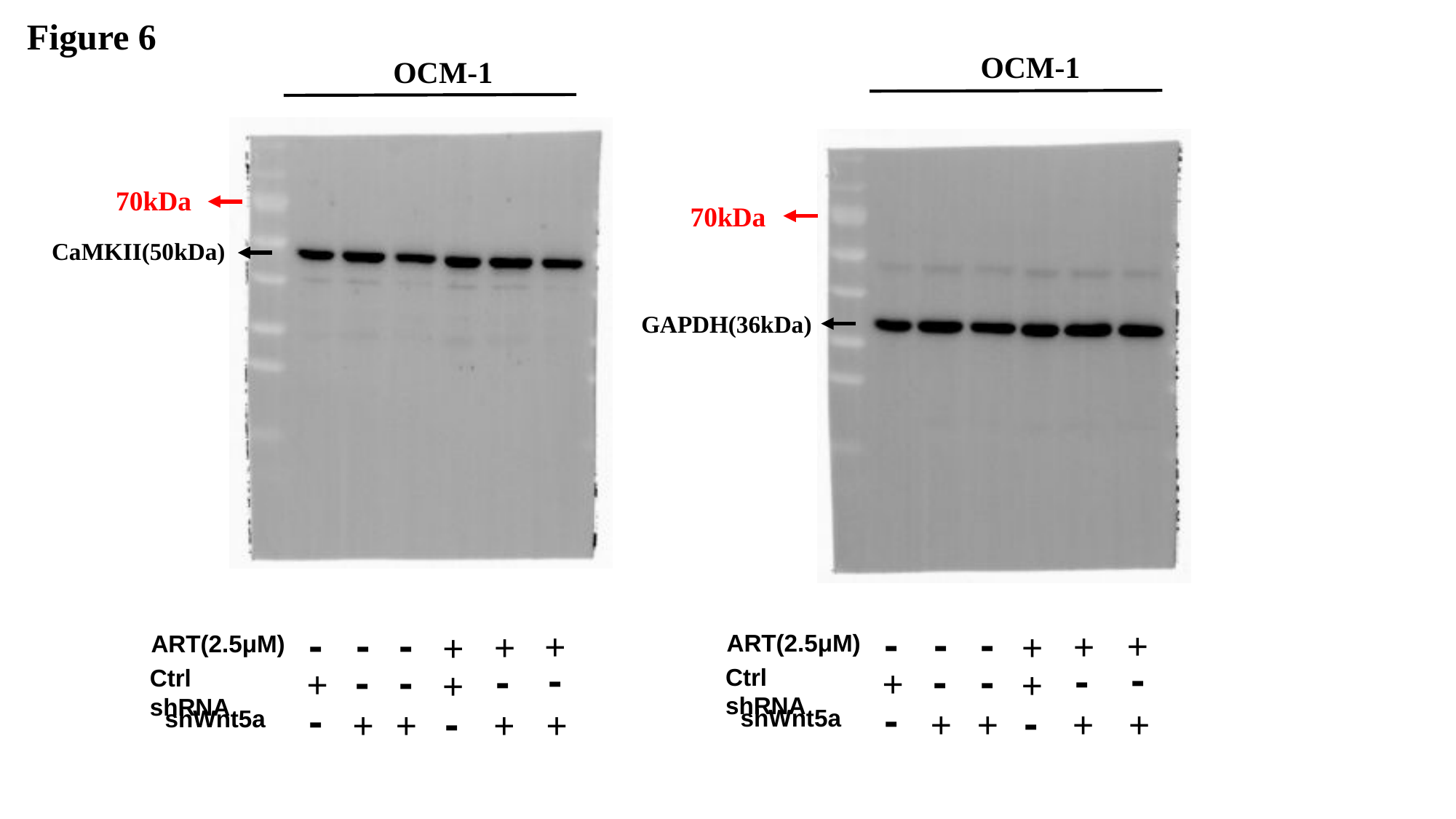

# Figure 6
OCM-1
OCM-1
70kDa
70kDa
CaMKII(50kDa)
GAPDH(36kDa)
Ctrl shRNA
Ctrl shRNA
-
-
-
-
-
-
+
+
+
+
+
+
ART(2.5μM)
ART(2.5μM)
-
-
-
-
-
-
-
-
+
shWnt5a
+
shWnt5a
+
+
-
-
-
-
+
+
+
+
+
+
+
+

## Slide 55
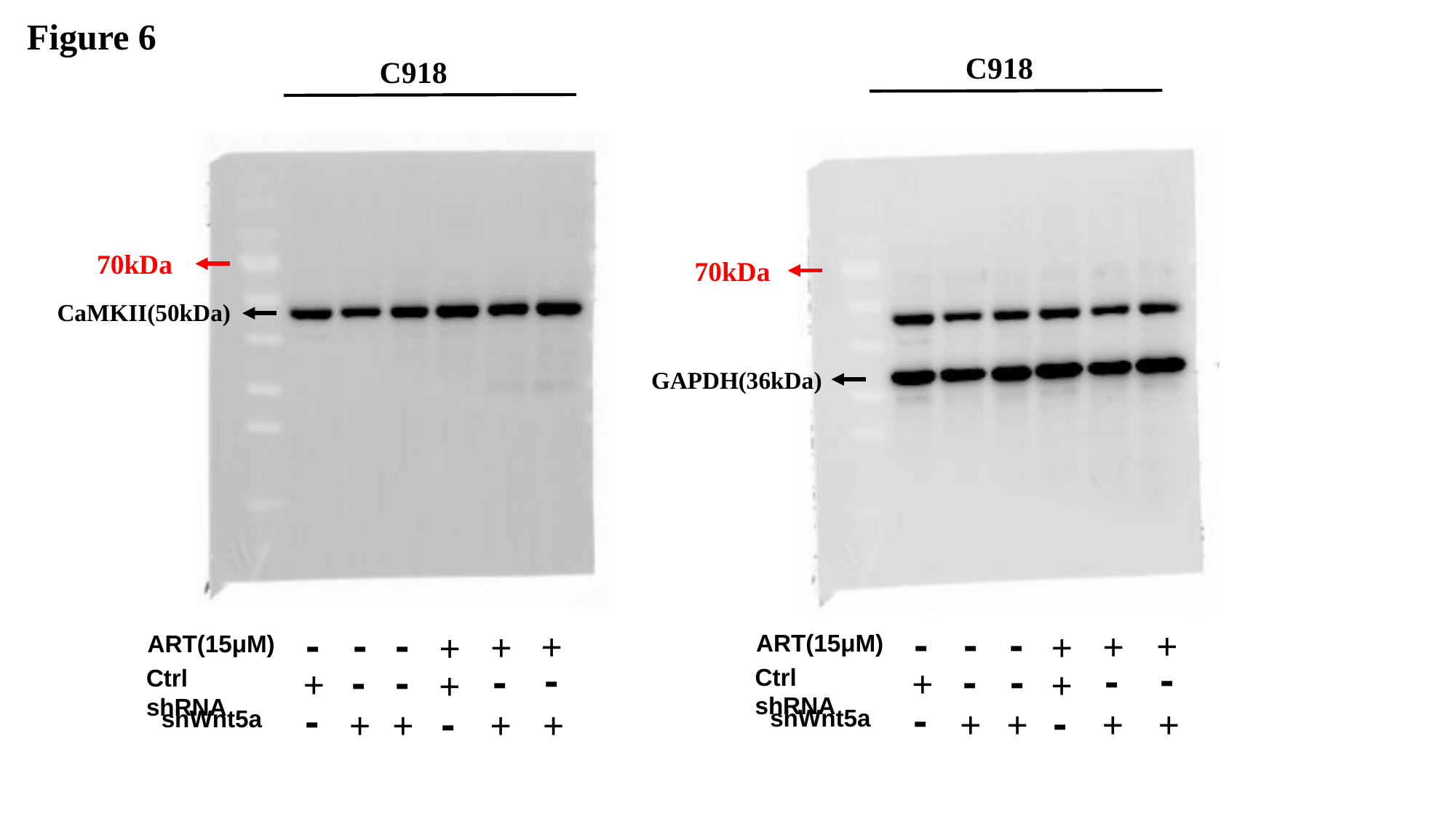

# Figure 6
C918
C918
70kDa
70kDa
CaMKII(50kDa)
GAPDH(36kDa)
Ctrl shRNA
Ctrl shRNA
-
-
-
-
-
-
+
+
+
+
+
+
ART(15μM)
ART(15μM)
-
-
-
-
-
-
-
-
+
shWnt5a
+
shWnt5a
+
+
-
-
-
-
+
+
+
+
+
+
+
+

## Slide 56
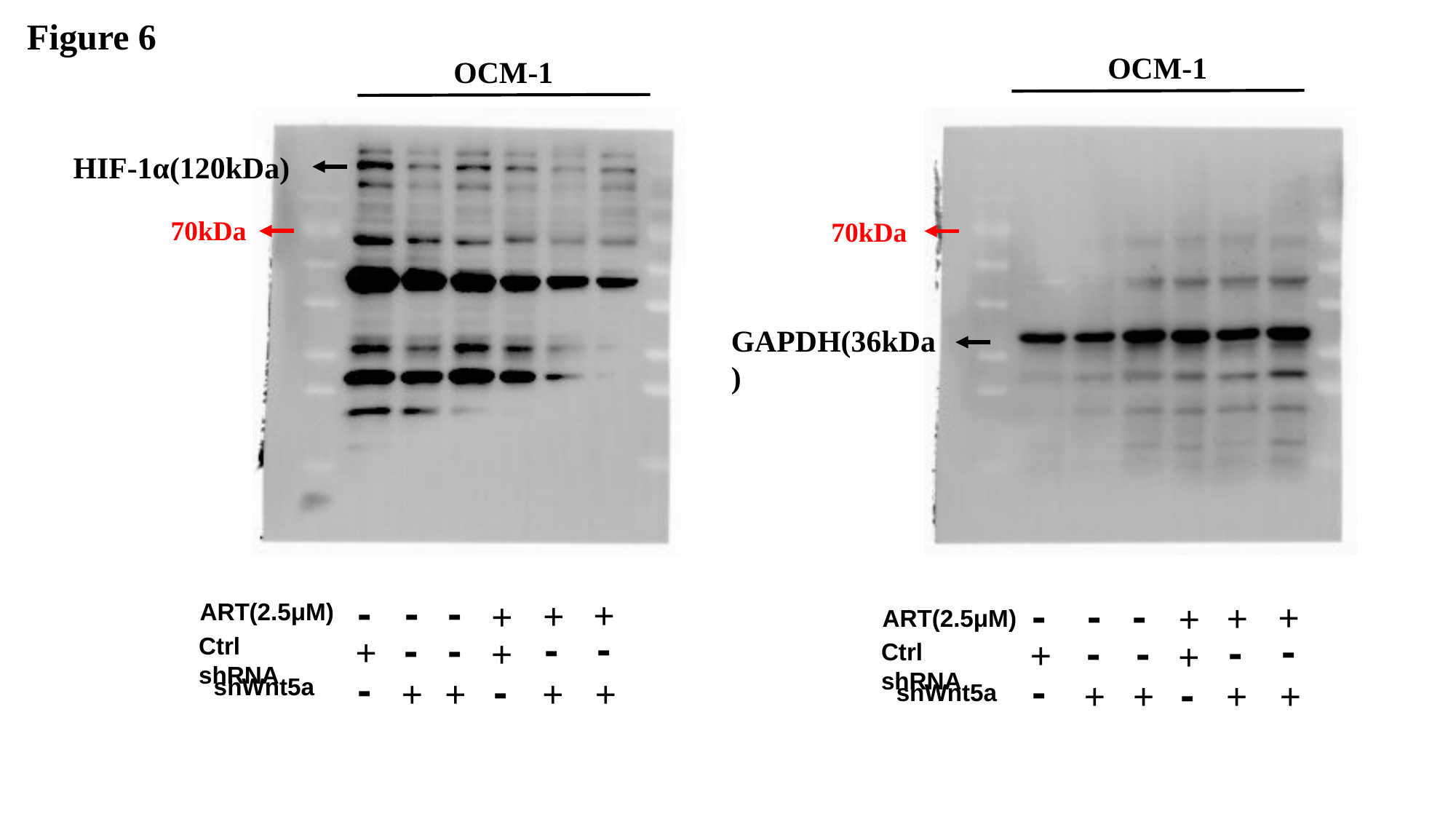

# Figure 6
OCM-1
OCM-1
HIF-1α(120kDa)
70kDa
70kDa
GAPDH(36kDa)
Ctrl shRNA
-
-
-
Ctrl shRNA
-
-
-
+
+
+
+
+
+
ART(2.5μM)
ART(2.5μM)
-
-
-
-
-
-
-
-
+
shWnt5a
+
+
+
shWnt5a
-
-
-
-
+
+
+
+
+
+
+
+

## Slide 57
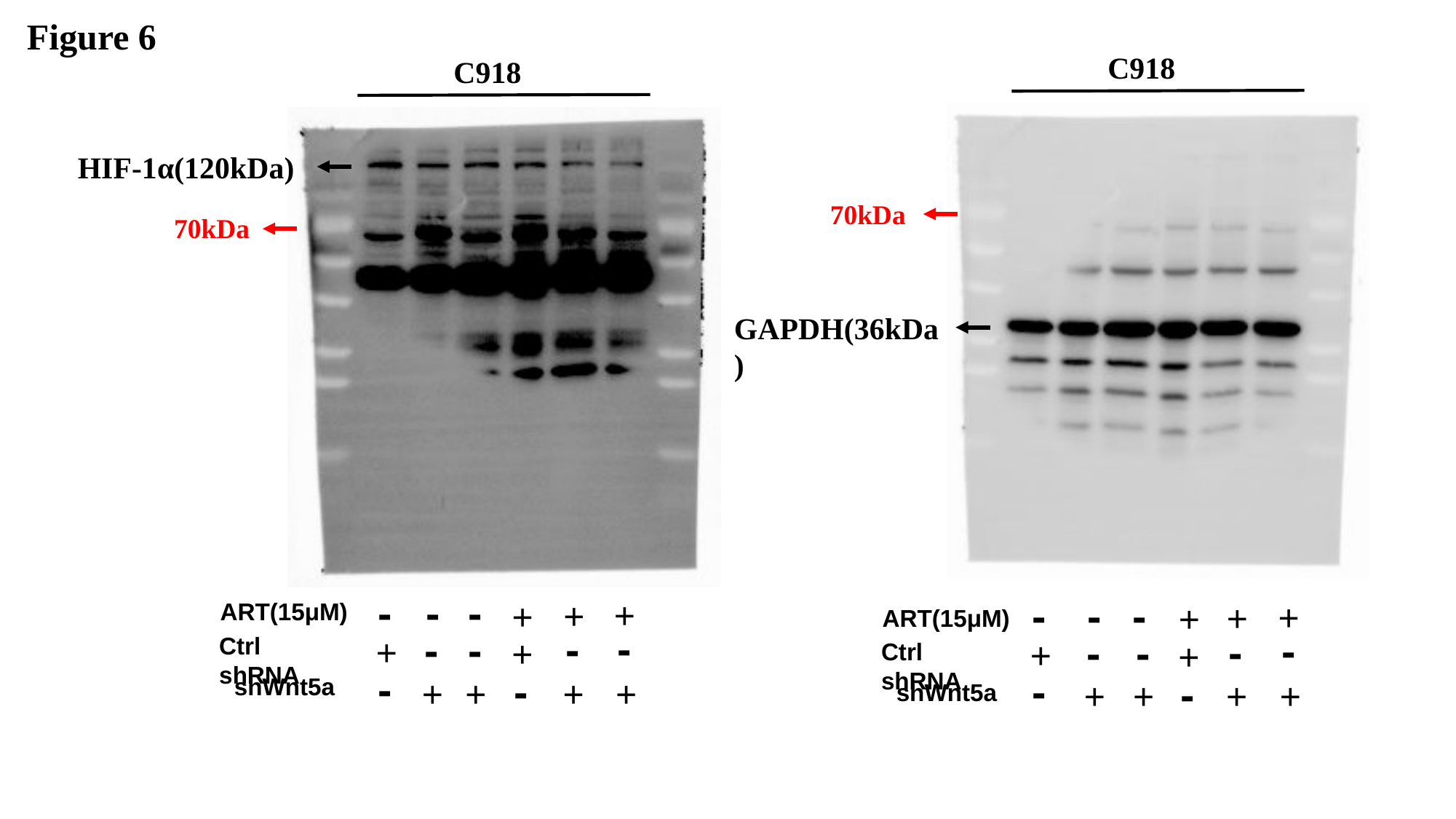

# Figure 6
C918
C918
HIF-1α(120kDa)
70kDa
70kDa
GAPDH(36kDa)
Ctrl shRNA
-
-
-
Ctrl shRNA
-
-
-
+
+
+
+
+
+
ART(15μM)
ART(15μM)
-
-
-
-
-
-
-
-
+
shWnt5a
+
+
+
shWnt5a
-
-
-
-
+
+
+
+
+
+
+
+

## Slide 58
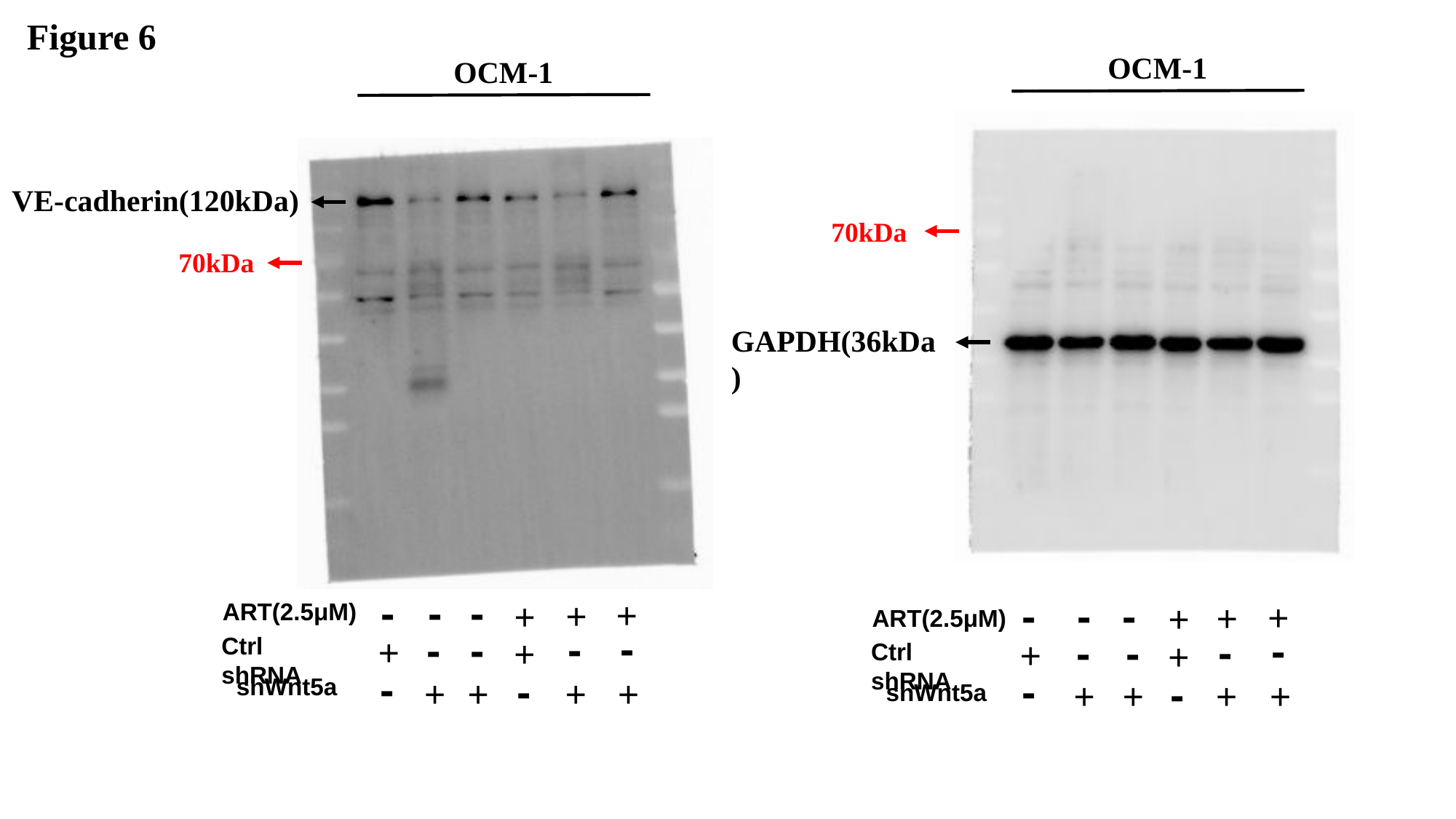

# Figure 6
OCM-1
OCM-1
VE-cadherin(120kDa)
70kDa
70kDa
GAPDH(36kDa)
Ctrl shRNA
-
-
-
Ctrl shRNA
-
-
-
+
+
+
+
+
+
ART(2.5μM)
ART(2.5μM)
-
-
-
-
-
-
-
-
+
shWnt5a
+
+
+
shWnt5a
-
-
-
-
+
+
+
+
+
+
+
+

## Slide 59
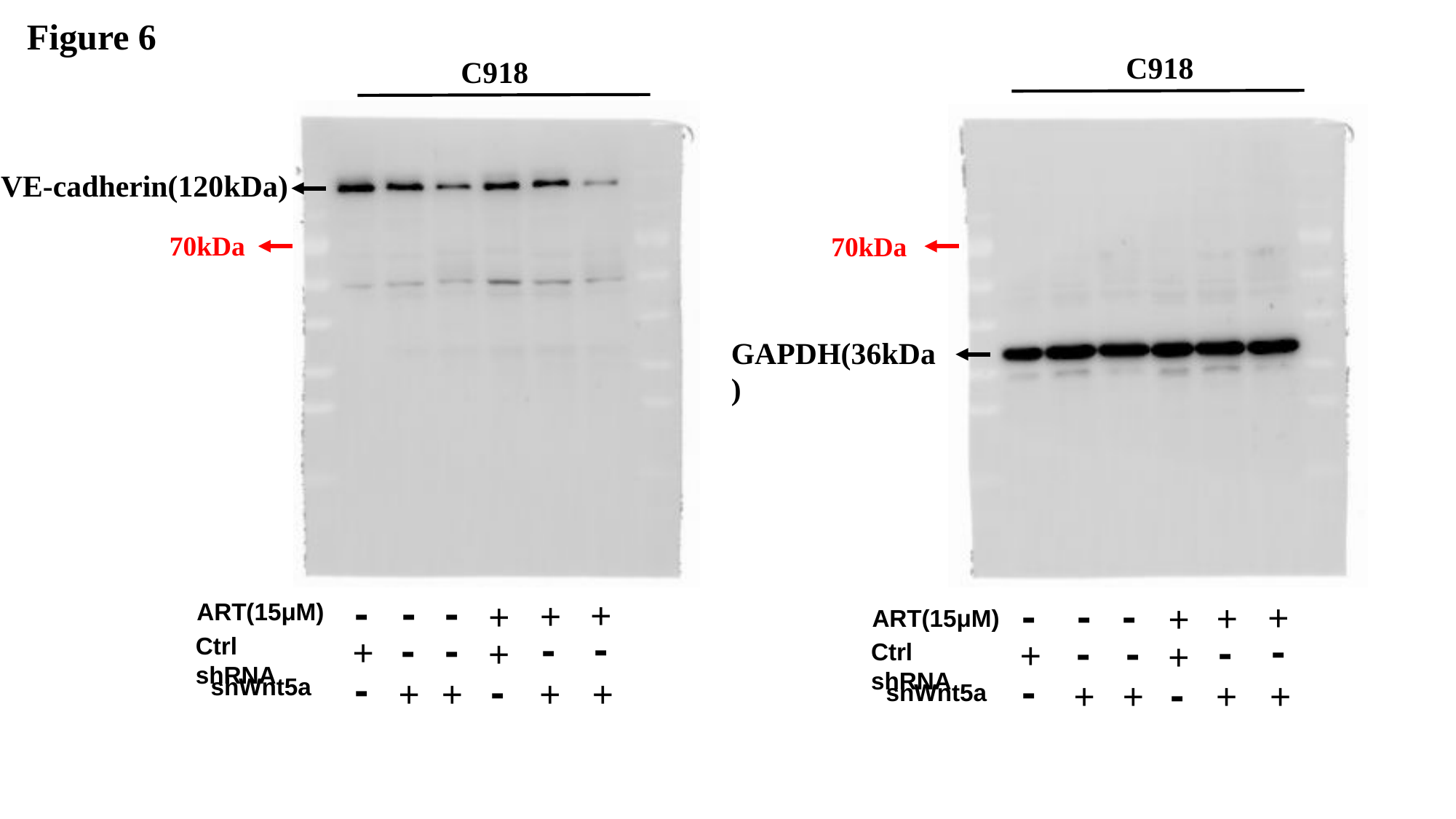

# Figure 6
C918
C918
VE-cadherin(120kDa)
70kDa
70kDa
GAPDH(36kDa)
Ctrl shRNA
-
-
-
Ctrl shRNA
-
-
-
+
+
+
+
+
+
ART(15μM)
ART(15μM)
-
-
-
-
-
-
-
-
+
shWnt5a
+
+
+
shWnt5a
-
-
-
-
+
+
+
+
+
+
+
+

## Slide 60
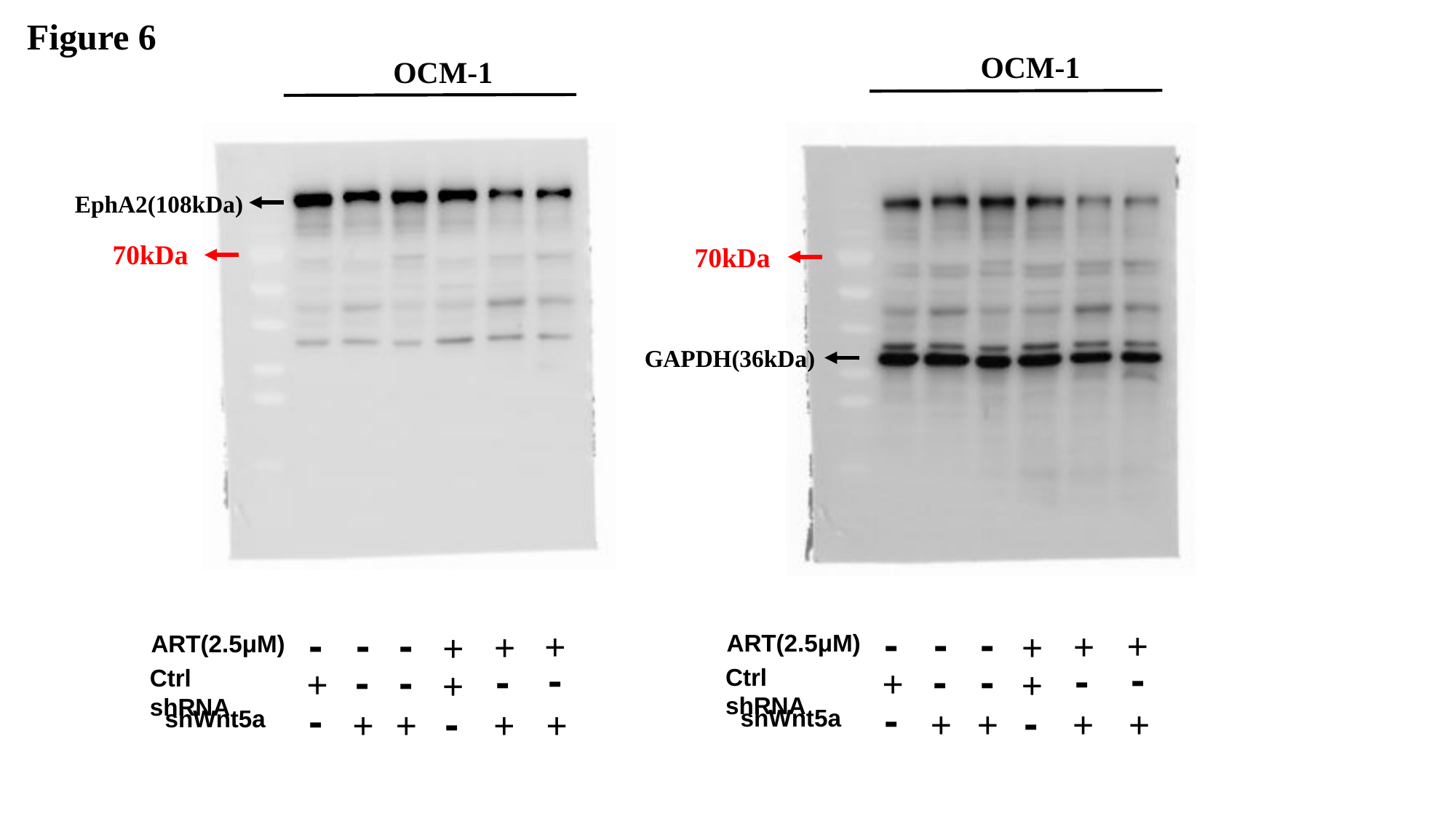

# Figure 6
OCM-1
OCM-1
EphA2(108kDa)
70kDa
70kDa
GAPDH(36kDa)
Ctrl shRNA
Ctrl shRNA
-
-
-
-
-
-
+
+
+
+
+
+
ART(2.5μM)
ART(2.5μM)
-
-
-
-
-
-
-
-
+
shWnt5a
+
shWnt5a
+
+
-
-
-
-
+
+
+
+
+
+
+
+

## Slide 61
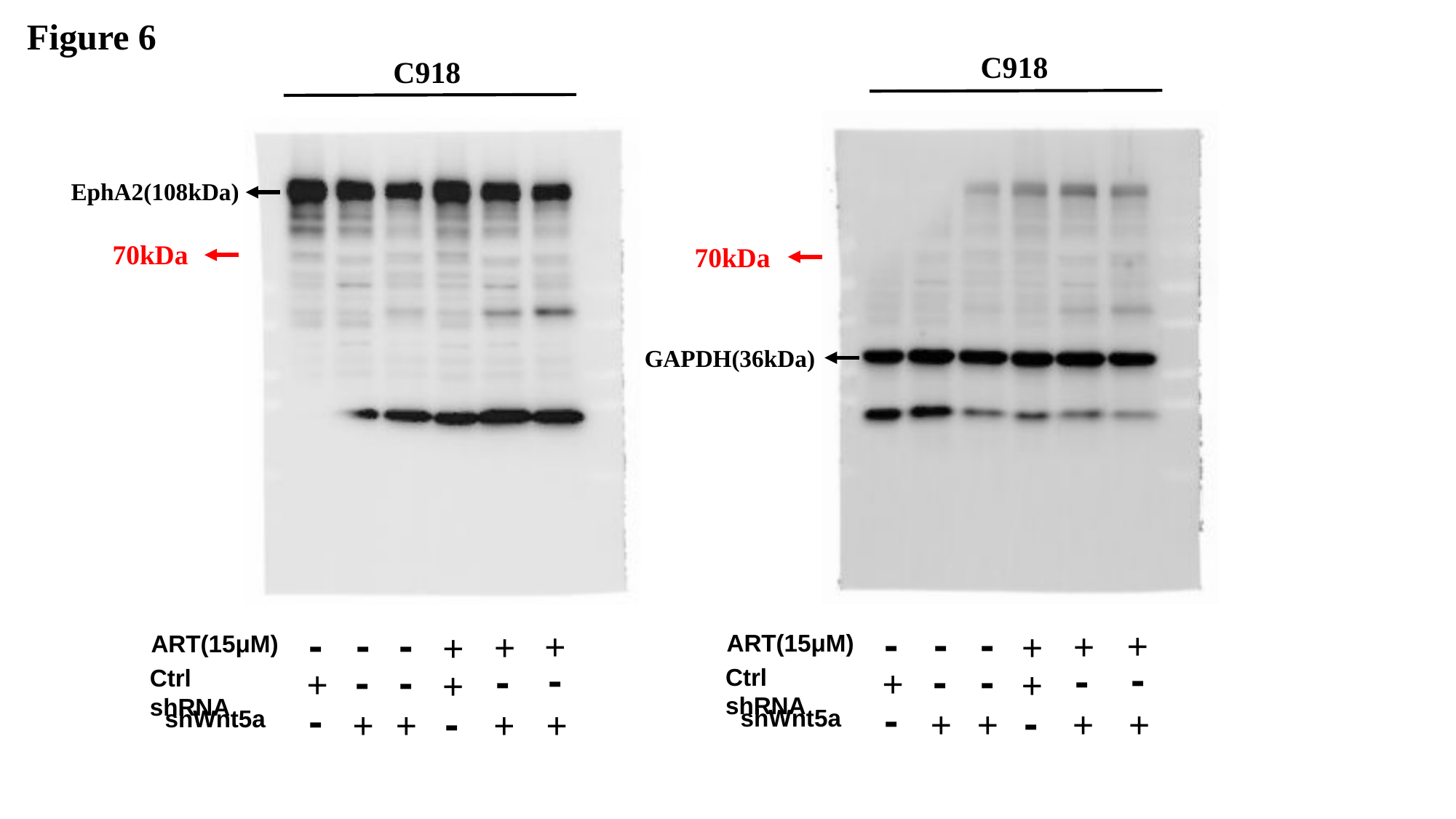

# Figure 6
C918
C918
EphA2(108kDa)
70kDa
70kDa
GAPDH(36kDa)
Ctrl shRNA
Ctrl shRNA
-
-
-
-
-
-
+
+
+
+
+
+
ART(15μM)
ART(15μM)
-
-
-
-
-
-
-
-
+
shWnt5a
+
shWnt5a
+
+
-
-
-
-
+
+
+
+
+
+
+
+

## Slide 62
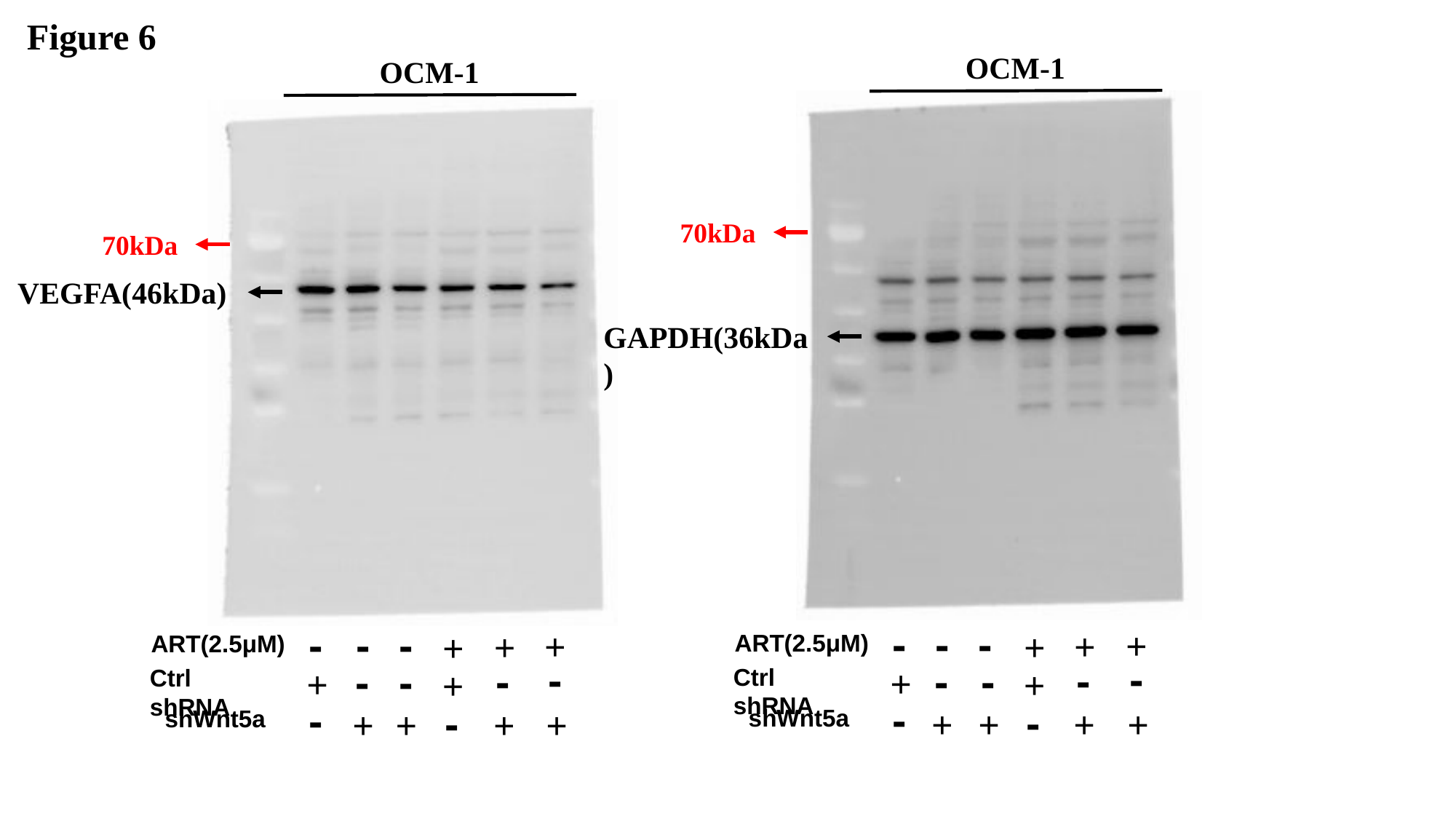

# Figure 6
OCM-1
OCM-1
70kDa
70kDa
VEGFA(46kDa)
GAPDH(36kDa)
Ctrl shRNA
Ctrl shRNA
-
-
-
-
-
-
+
+
+
+
+
+
ART(2.5μM)
ART(2.5μM)
-
-
-
-
-
-
-
-
+
shWnt5a
+
shWnt5a
+
+
-
-
-
-
+
+
+
+
+
+
+
+

## Slide 63
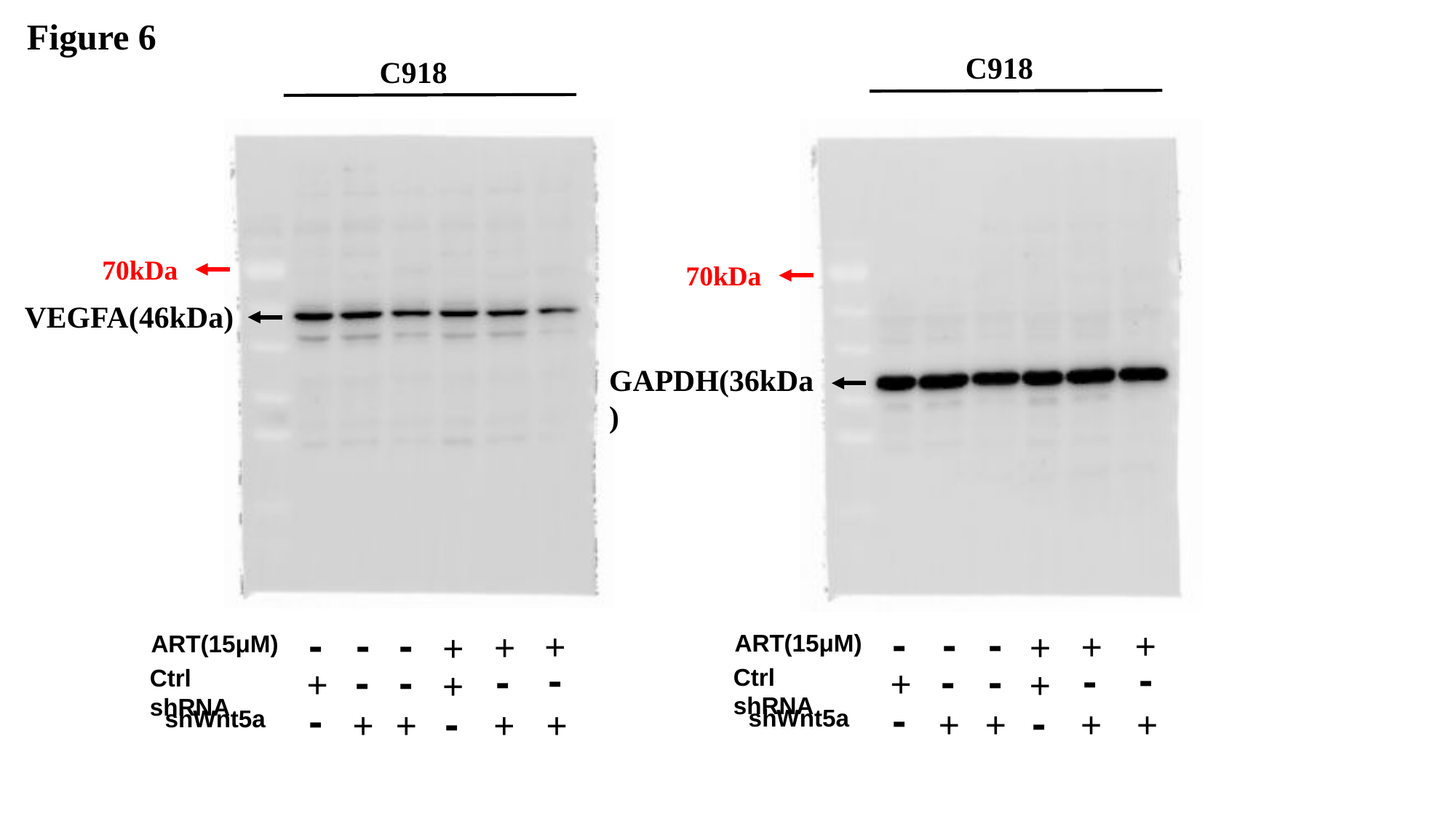

# Figure 6
C918
C918
70kDa
70kDa
VEGFA(46kDa)
GAPDH(36kDa)
Ctrl shRNA
Ctrl shRNA
-
-
-
-
-
-
+
+
+
+
+
+
ART(15μM)
ART(15μM)
-
-
-
-
-
-
-
-
+
shWnt5a
+
shWnt5a
+
+
-
-
-
-
+
+
+
+
+
+
+
+

## Slide 64
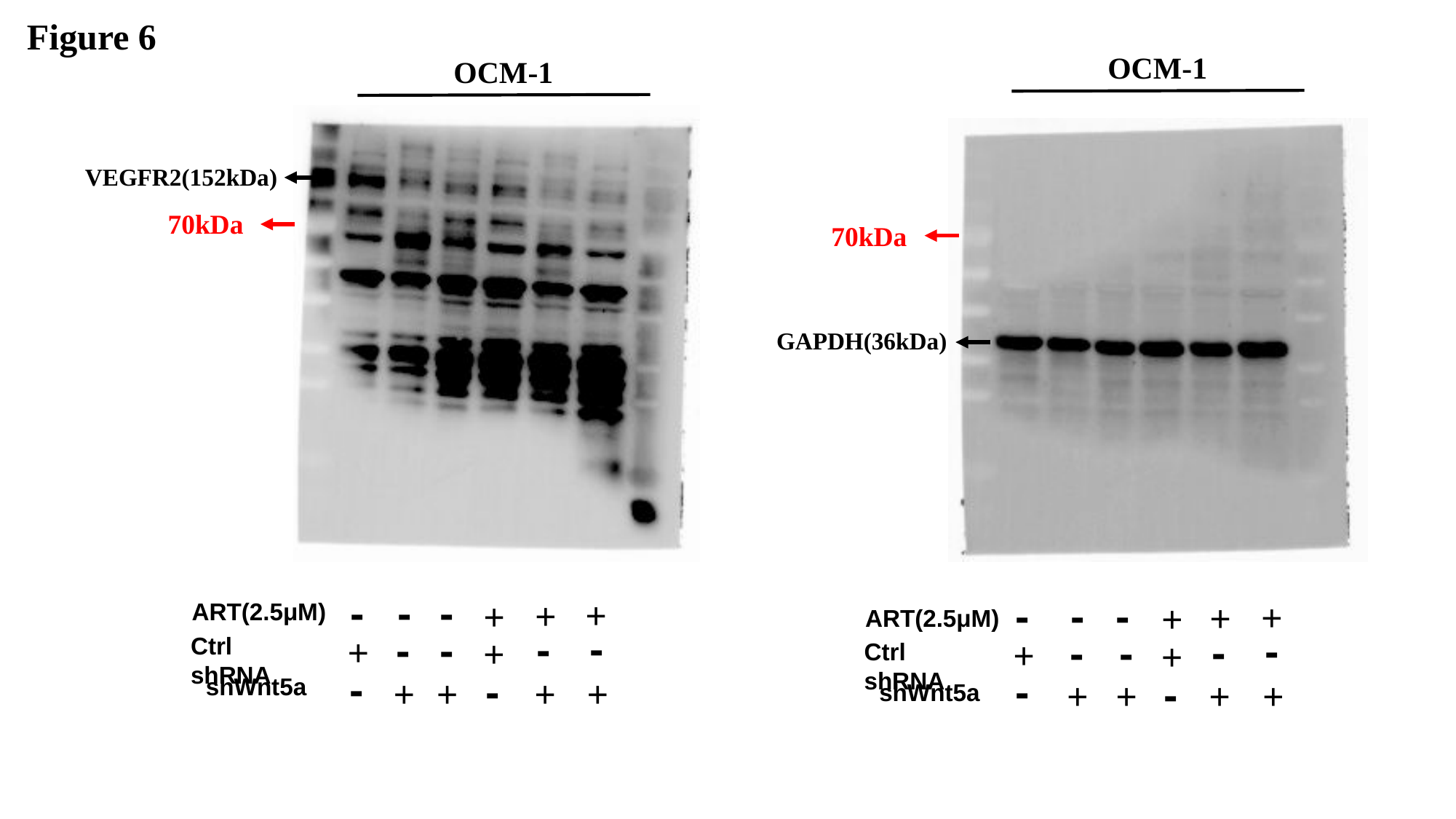

# Figure 6
OCM-1
OCM-1
VEGFR2(152kDa)
70kDa
70kDa
GAPDH(36kDa)
Ctrl shRNA
-
-
-
Ctrl shRNA
-
-
-
+
+
+
+
+
+
ART(2.5μM)
ART(2.5μM)
-
-
-
-
-
-
-
-
+
shWnt5a
+
+
+
shWnt5a
-
-
-
-
+
+
+
+
+
+
+
+

## Slide 65
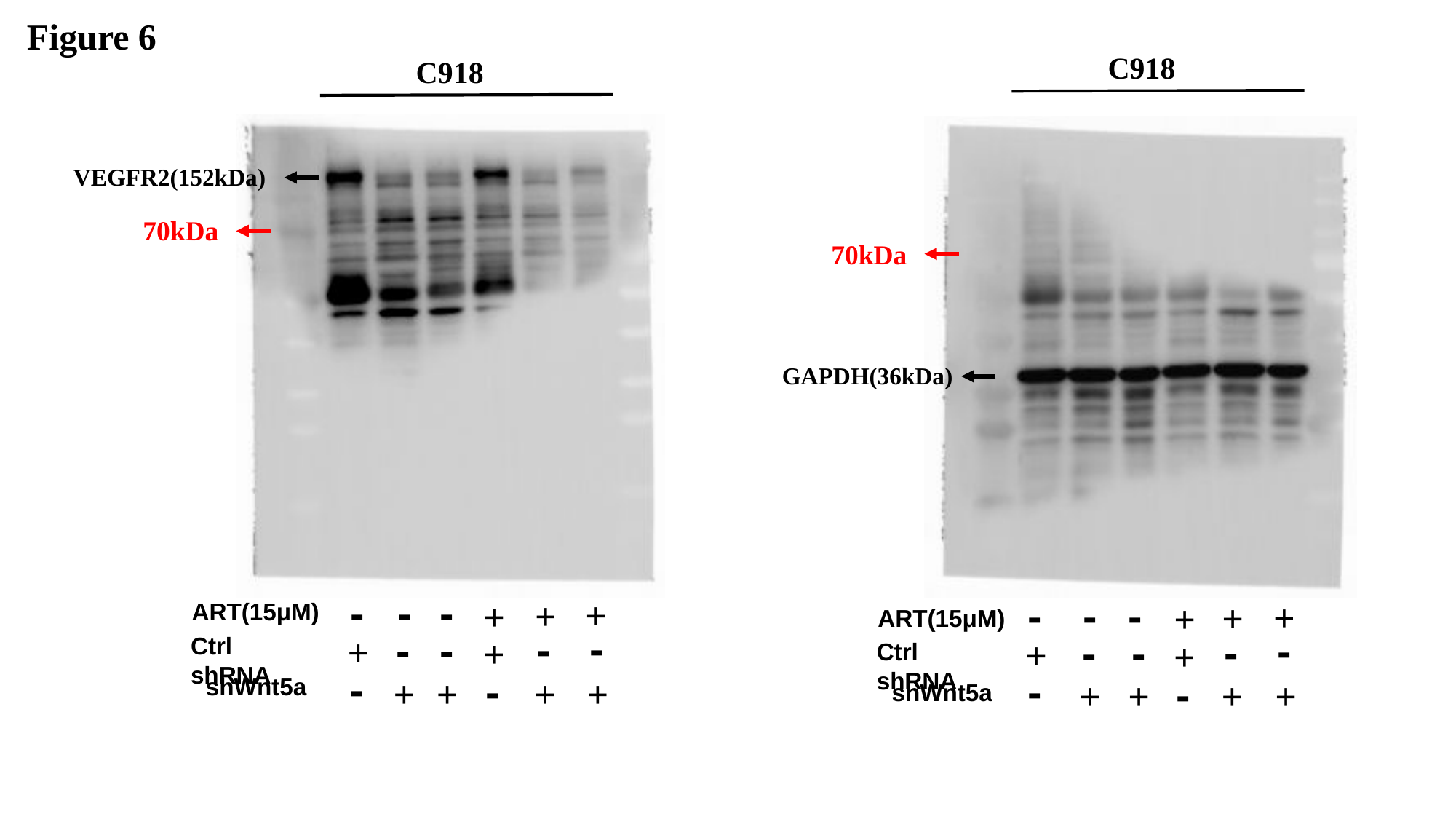

# Figure 6
C918
C918
VEGFR2(152kDa)
70kDa
70kDa
GAPDH(36kDa)
Ctrl shRNA
-
-
-
Ctrl shRNA
-
-
-
+
+
+
+
+
+
ART(15μM)
ART(15μM)
-
-
-
-
-
-
-
-
+
shWnt5a
+
+
+
shWnt5a
-
-
-
-
+
+
+
+
+
+
+
+

## Slide 66
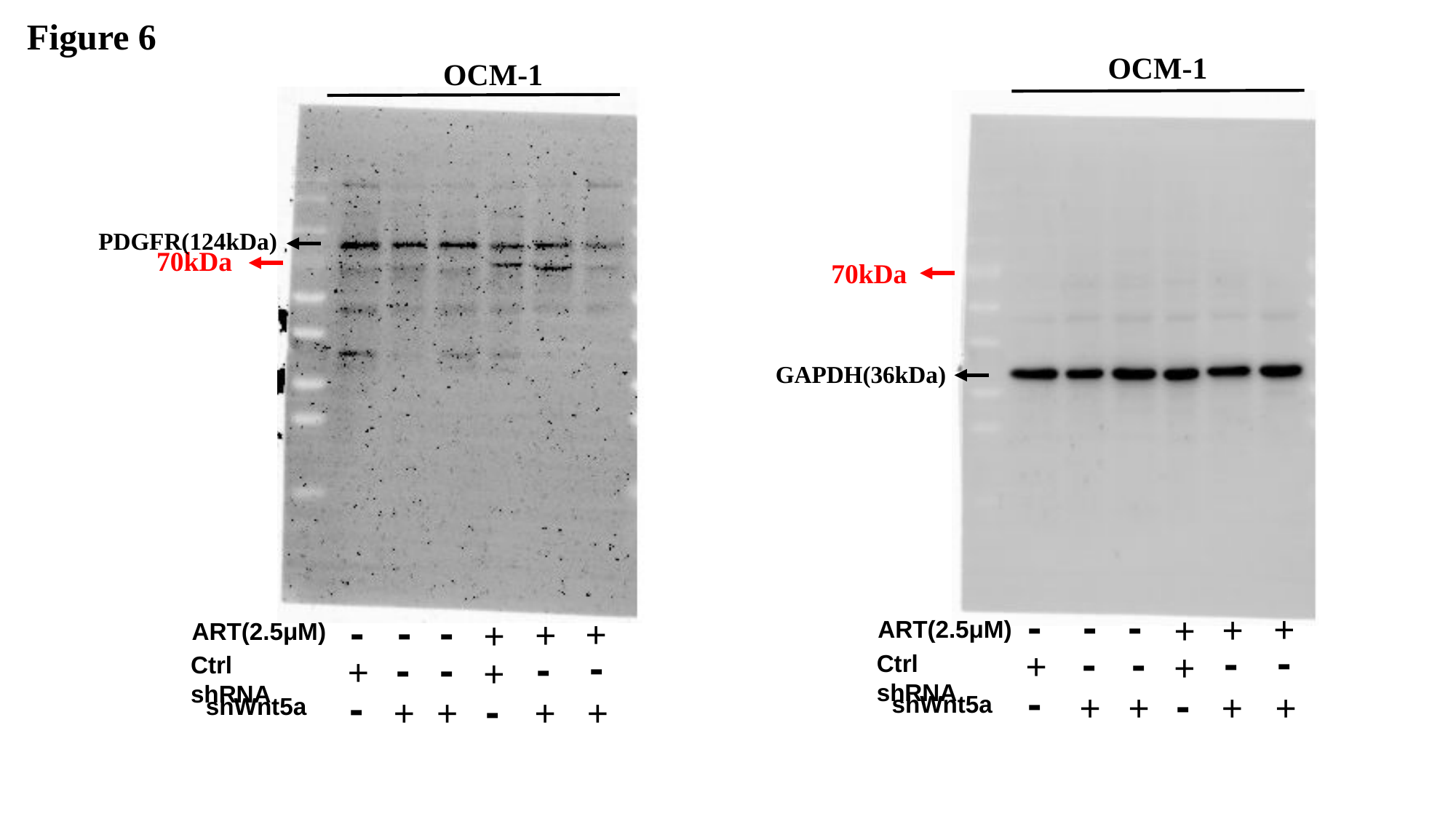

# Figure 6
OCM-1
OCM-1
PDGFR(124kDa)
70kDa
70kDa
GAPDH(36kDa)
Ctrl shRNA
Ctrl shRNA
-
-
-
-
-
-
+
+
+
+
+
+
ART(2.5μM)
ART(2.5μM)
-
-
-
-
-
-
-
-
+
+
shWnt5a
+
shWnt5a
+
-
-
-
-
+
+
+
+
+
+
+
+

## Slide 67
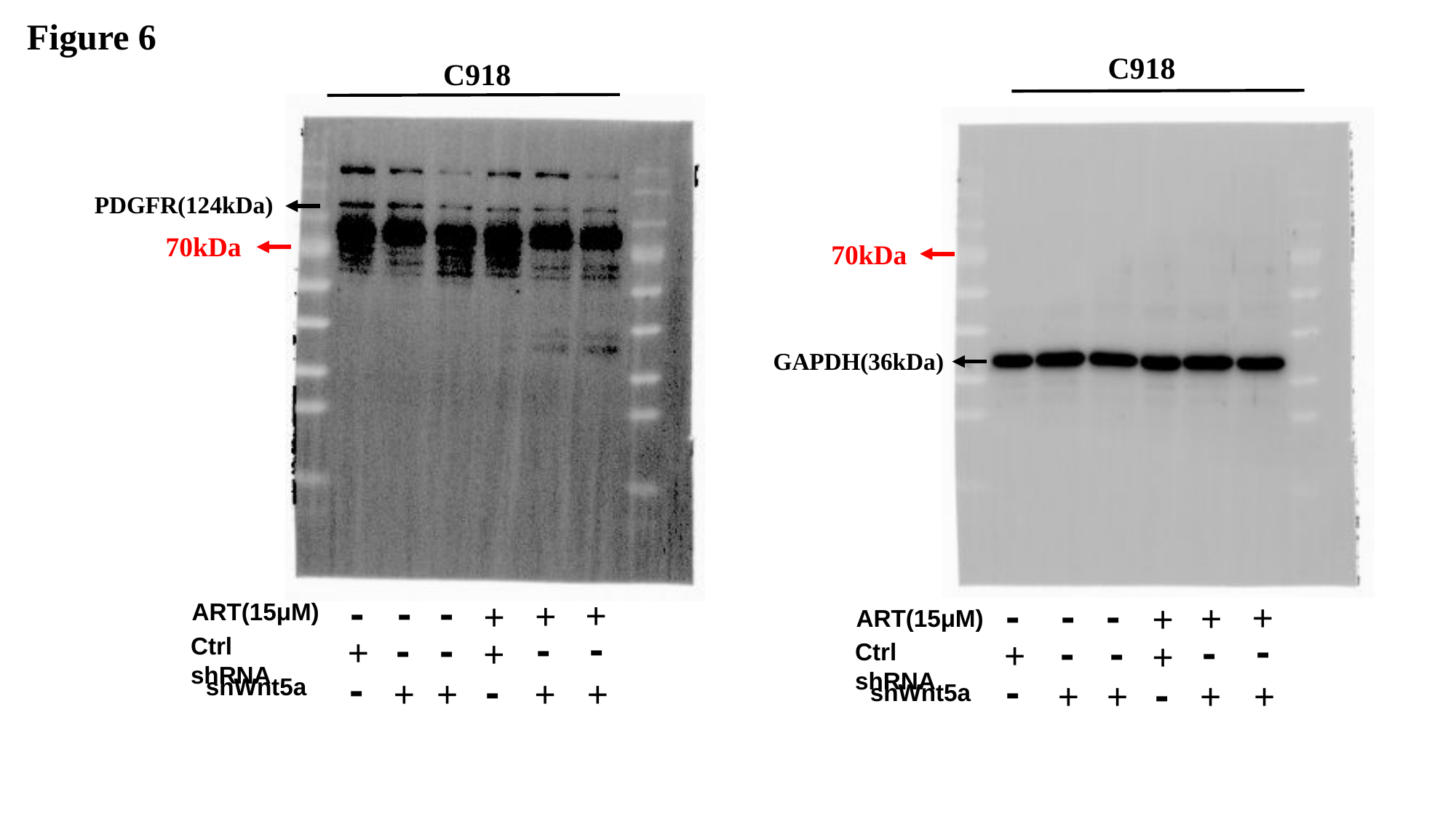

# Figure 6
C918
C918
PDGFR(124kDa)
70kDa
70kDa
GAPDH(36kDa)
Ctrl shRNA
-
-
-
Ctrl shRNA
-
-
-
+
+
+
+
+
+
ART(15μM)
ART(15μM)
-
-
-
-
-
-
-
-
+
shWnt5a
+
+
+
shWnt5a
-
-
-
-
+
+
+
+
+
+
+
+

## Slide 68
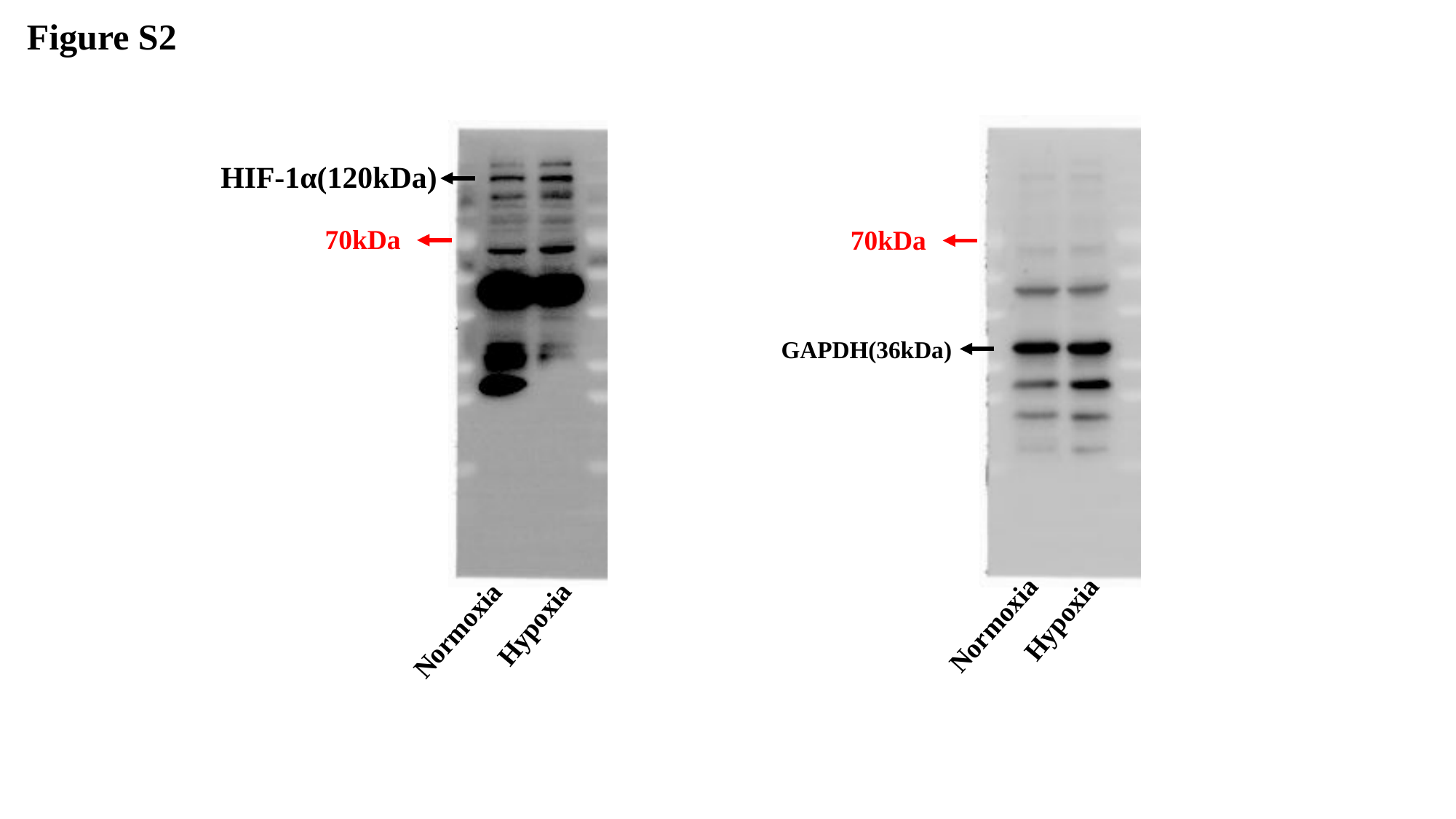

# Figure S2
HIF-1α(120kDa)
70kDa
70kDa
GAPDH(36kDa)
Hypoxia
Hypoxia
Normoxia
Normoxia

## Slide 69
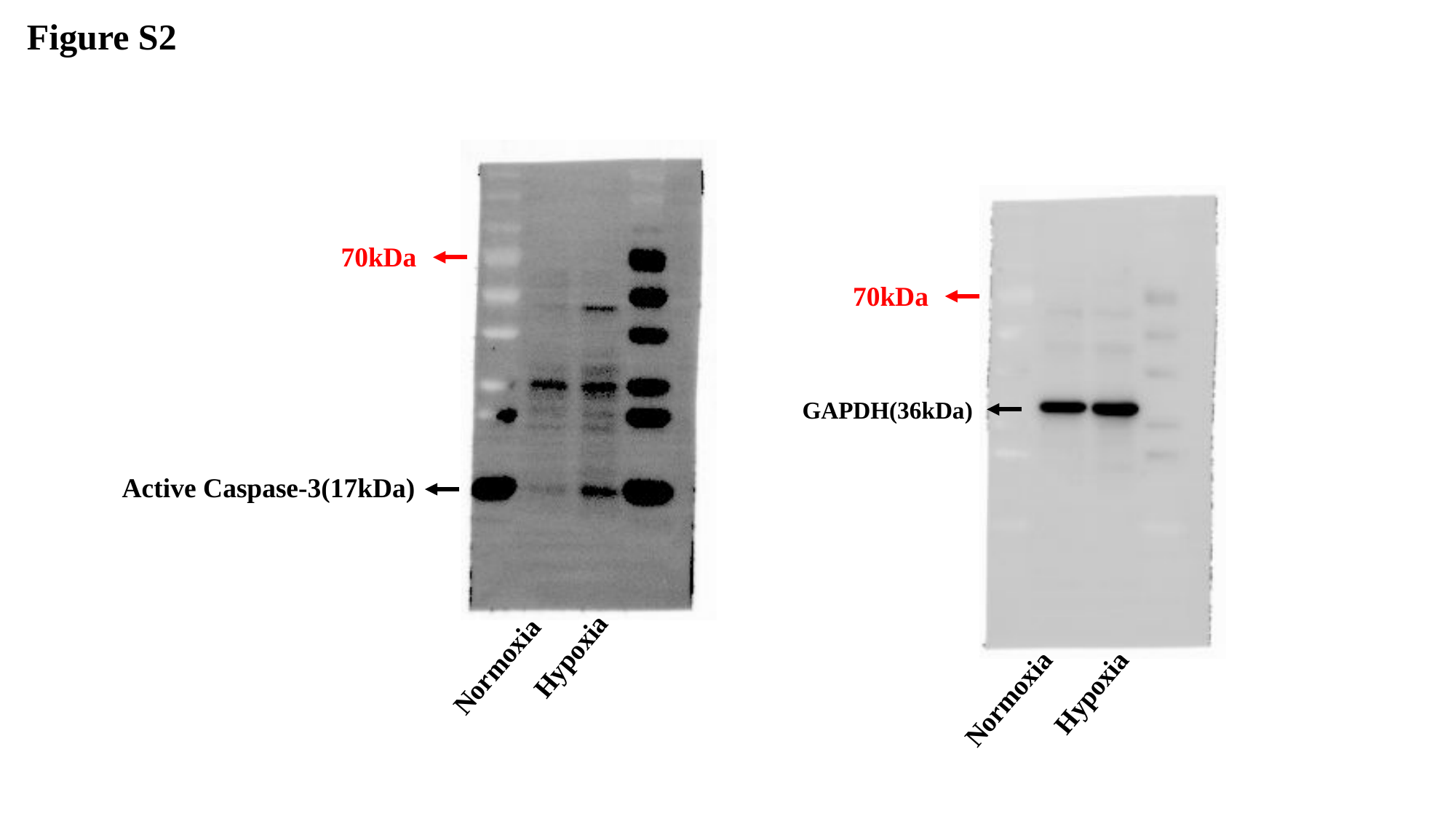

# Figure S2
70kDa
70kDa
GAPDH(36kDa)
Active Caspase-3(17kDa)
Hypoxia
Normoxia
Hypoxia
Normoxia

## Slide 70
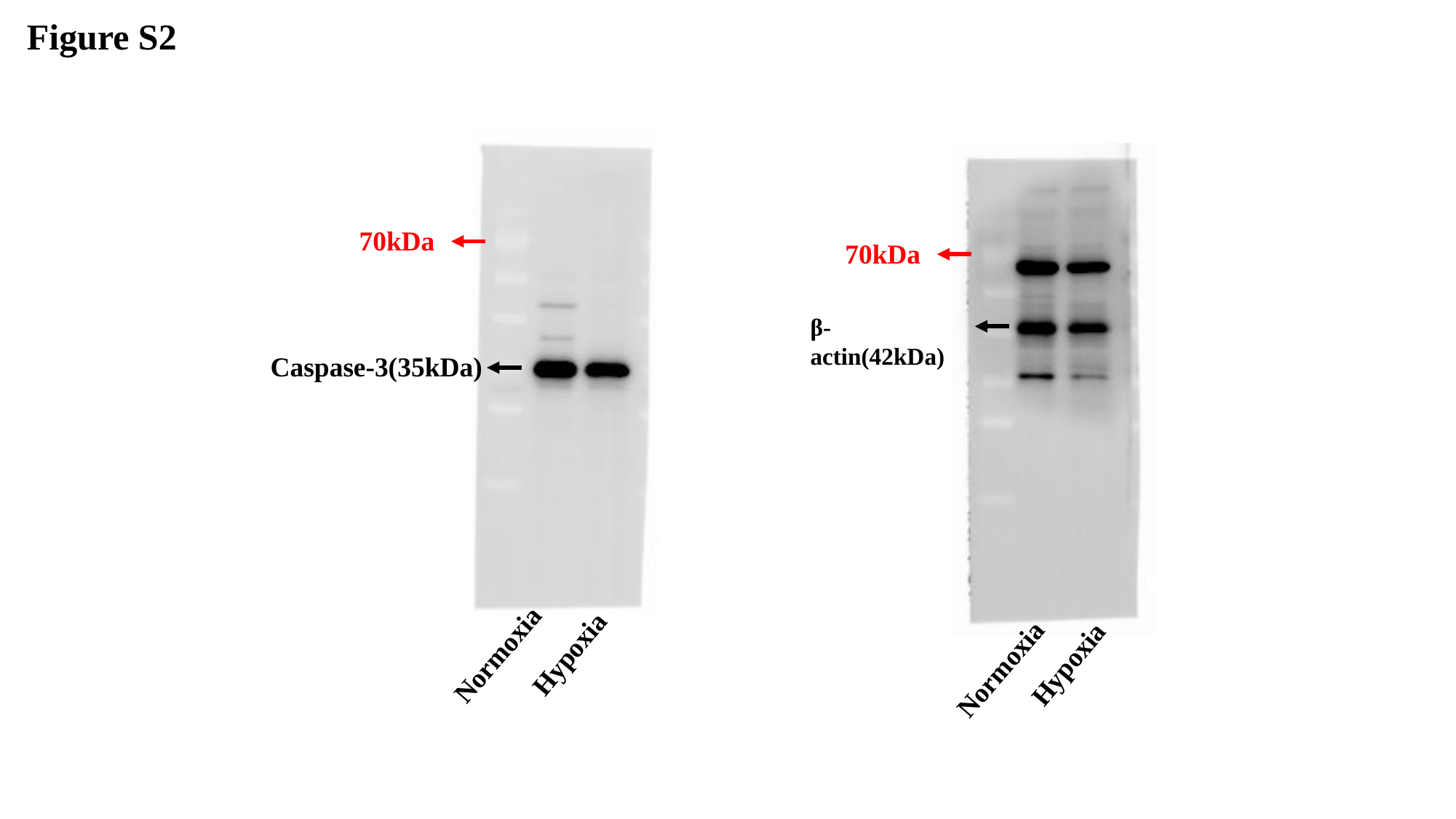

# Figure S2
70kDa
70kDa
β-actin(42kDa)
Caspase-3(35kDa)
Hypoxia
Normoxia
Hypoxia
Normoxia

## Slide 71
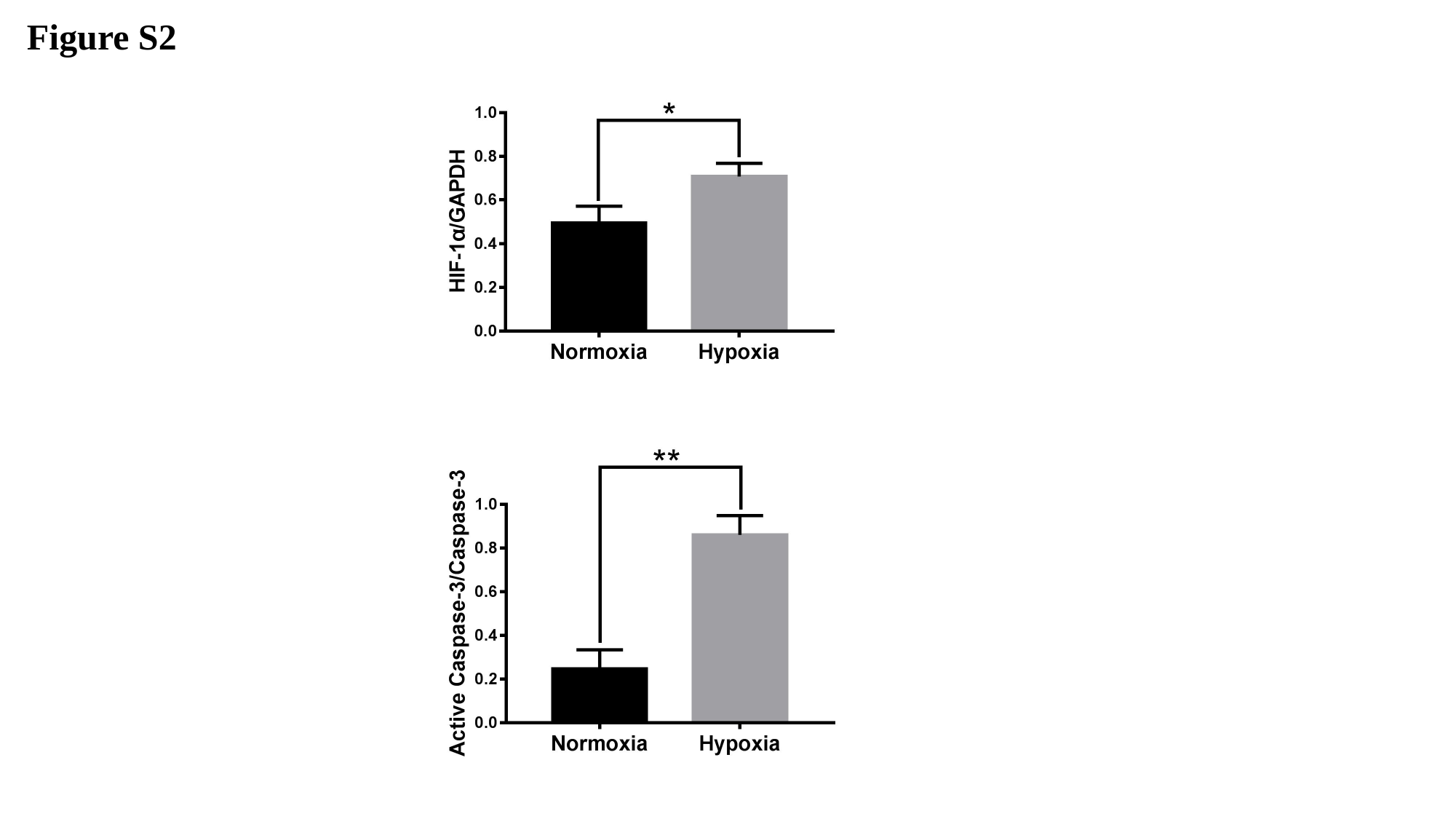

# Figure S2
