# Supplementary figures and images for: Artesunate Suppresses Choroidal Melanoma Vasculogenic Mimicry Formation and Angiogenesis via the Wnt/CaMKII Signaling Axis
Source: Front Oncol. 2021 Aug 12;11:714646. doi: 10.3389/fonc.2021.714646 (PMC8406848; doi:10.3389/fonc.2021.714646)

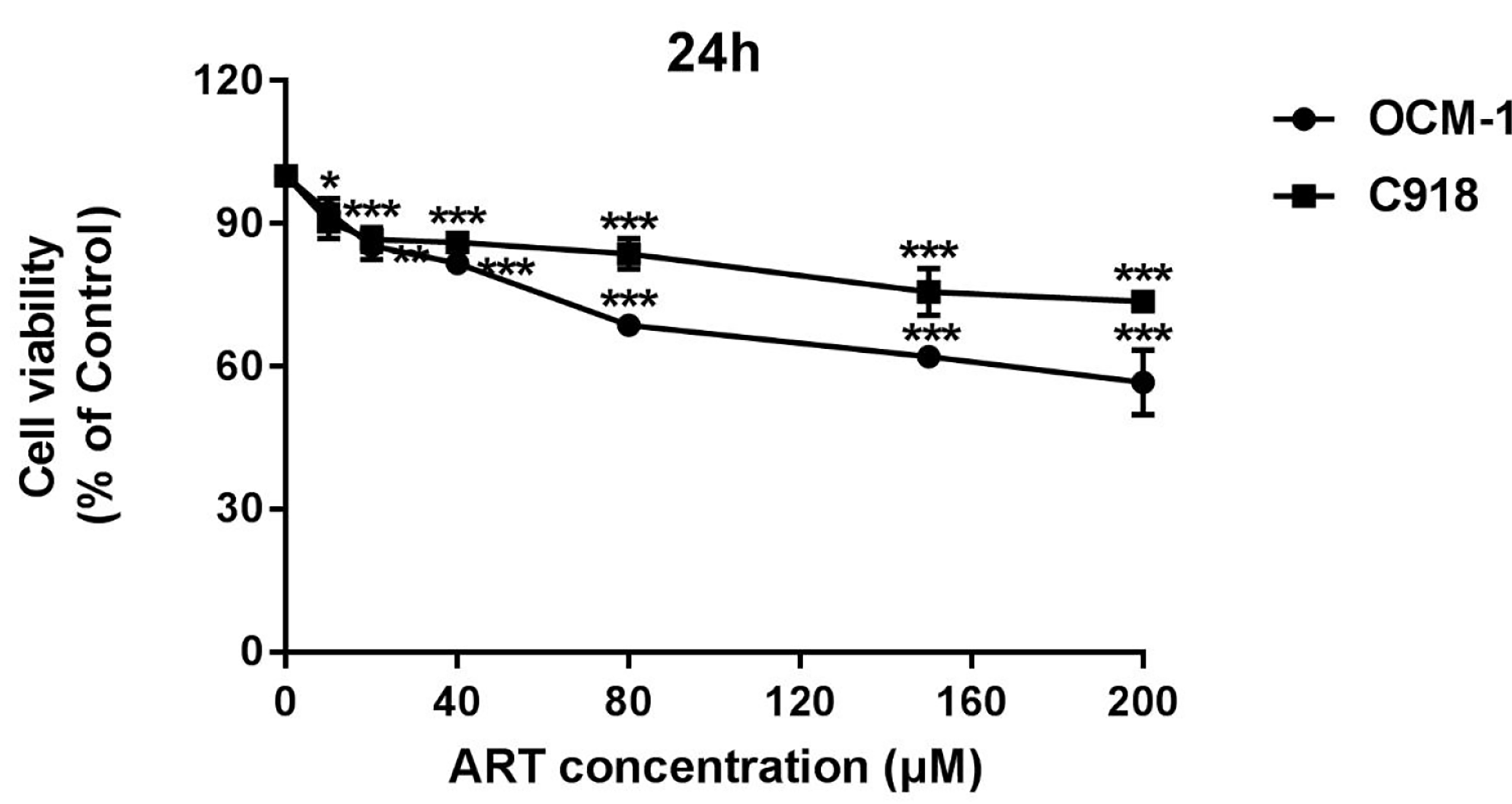

Supplement: Supplementary file 1 [file DataSheet_1.zip › Supplementary materials/Figure S1.tif]

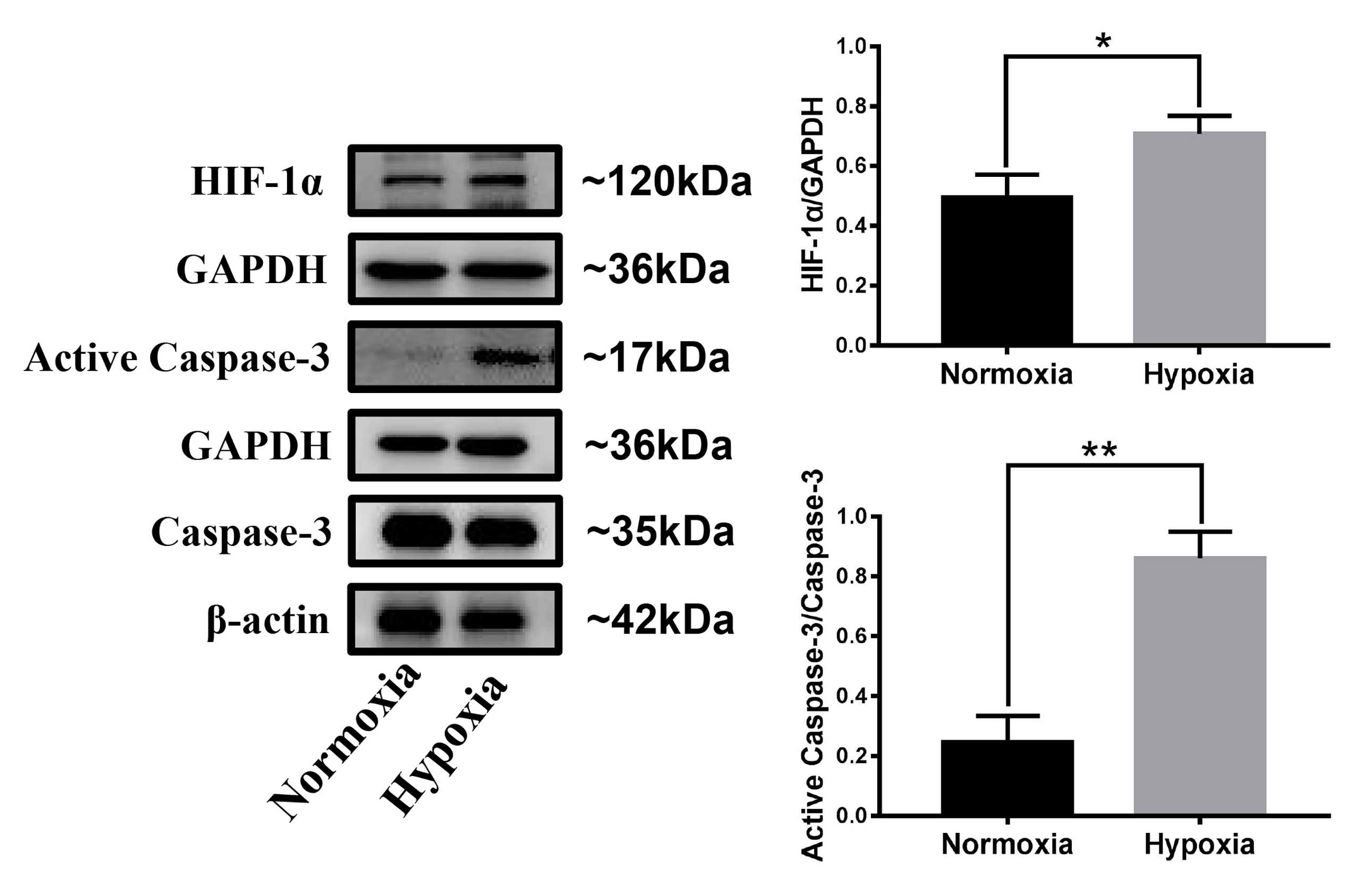

Supplement: Supplementary file 1 [file DataSheet_1.zip › Supplementary materials/Figure S2.tif]
