# Supplementary material for: Artesunate Suppresses Choroidal Melanoma Vasculogenic Mimicry Formation and Angiogenesis via the Wnt/CaMKII Signaling Axis
Source: Front Oncol. 2021 Aug 12;11:714646. doi: 10.3389/fonc.2021.714646 (PMC8406848; doi:10.3389/fonc.2021.714646)
Supplement: Supplementary file 2 [file Table_1.doc]

**Supplementary materials**

**Supplementary Table 1** Human-specific primer sequences of seven pro-angiogenic factors used in the qPCR reaction.

**Supplementary Table 1 Human-Specific Sense and Antisense Primers**

| Gene | Forward primer type（5’→ 3’） | Reverse primer type (5’→ 3’) |
| --- | --- | --- |
| VEGF-A | AGGGCAGAATCATCACGAAGT | AGGGTCTCGATTGGATGGCA |
| PDGF-B | CTCGATCCGCTCCTTTGATGA | CGTTGGTGCGGTCTATGAG |
| CXCL1 | CAGGGAATTCACCCCAAGAACA | GGATGCAGGATTGAGGCAAGC |
| TGFβ | CTAATGGTGGAAACCCACAACG | TATCGCCAGGAATTGTTGCTG |
| SCF | AATCCTCTCGTCAAAACTGAAGG | CCATCTCGCTTATCCAACAATGA |
| IGF1 | GCTCTTCAGTTCGTGTGTGGA | GCCTCCTTAGATCACAGCTCC |
| HGF | GCTATCGGGGTAAAGACCTACA | CGTAGCGTACCTCTGGATTGC |
