# Supplementary material for: Artesunate Suppresses Choroidal Melanoma Vasculogenic Mimicry Formation and Angiogenesis via the Wnt/CaMKII Signaling Axis
Source: Front Oncol. 2021 Aug 12;11:714646. doi: 10.3389/fonc.2021.714646 (PMC8406848; doi:10.3389/fonc.2021.714646)
Supplement: Supplementary file 1 [file DataSheet_1.zip › Supplementary materials/Supplementary materials.doc]

**Supplementary materials**

**Supplementary Table 1** Human-specific primer sequences of seven pro-angiogenic factors used in the RT-PCR reaction.

**Figure S1** Proliferation inhibition by ART in choroidal melanoma cells for 24h. OCM-1 and C918 cells were treated with different concentrations of ART (0, 10, 20, 40, 80, 150, and 200 μM) for 24 h. Cell viability was measured by CCK-8 assay. The data are from three independent experiments (mean ± SEM); *P＜0.05, **P＜0.01, ***P＜0.001.

**Figure S2** Hypoxia led to the activation of caspase-3 in OCM-1 cells. OCM-1 cells were cultured under hypoxic conditions for 6 h. Western blot showed that hypoxia led to activation of caspase-3 and up-regulation of HIF-1α, supporting the occurrence of apoptosis. The results are represented as the mean ± SEM from three independent samples. *P＜0.05, **P＜0.01, ***P＜0.001.

**Supplementary Table 1 Human-Specific Sense (S)and Antisense (AS)Primers**

| Human transcript | Forward primer type（5’→ 3’） | Reverse primer type (5’→ 3’) |
| --- | --- | --- |
| VEGF-A | AGGGCAGAATCATCACGAAGT | AGGGTCTCGATTGGATGGCA |
| PDGF-B | CTCGATCCGCTCCTTTGATGA | CGTTGGTGCGGTCTATGAG |
| CXCL1 | CAGGGAATTCACCCCAAGAACA | GGATGCAGGATTGAGGCAAGC |
| TGFβ | CTAATGGTGGAAACCCACAACG | TATCGCCAGGAATTGTTGCTG |
| SCF | AATCCTCTCGTCAAAACTGAAGG | CCATCTCGCTTATCCAACAATGA |
| IGF1 | GCTCTTCAGTTCGTGTGTGGA | GCCTCCTTAGATCACAGCTCC |
| HGF | GCTATCGGGGTAAAGACCTACA | CGTAGCGTACCTCTGGATTGC |

**Figure S1**

**Figure S2**
